# Natal origin and age-specific egress of Pacific bluefin tuna from coastal nurseries revealed with geochemical markers

**DOI:** 10.1038/s41598-021-93298-2

**Published:** 2021-07-09

**Authors:** Jay R. Rooker, R. J. David Wells, Barbara A. Block, Hui Liu, Hannes Baumann, Wei-Chuan Chiang, Michelle Zapp Sluis, Nathaniel R. Miller, John A. Mohan, Seiji Ohshimo, Yosuke Tanaka, Michael A. Dance, Heidi Dewar, Owyn E. Snodgrass, Jen-Chieh Shiao

**Affiliations:** 1 Department of Marine Biology, Texas A&M University at Galveston, 200 Seawolf Parkway, Galveston, TX 77553 USA; 2 Department of Ecology and Conservation Biology, Texas A&M University, College Station, TX 77843-2258 USA; 3 Hopkins Marine Station, Stanford University, 120 Oceanview Boulevard, Pacific Grove, CA 93950 USA; 4 Department of Marine Sciences, University of Connecticut, 1080 Shennecossett Road, Groton, CT 06340-6048 USA; 5 Council of Agriculture, Fisheries Research Institute TW, 199 Hou-Ih Road, Keelung, 20246 Taiwan; 6 Jackson School of Geosciences, The University of Texas at Austin, 2275 Speedway Stop C9000, Austin, TX 78712 USA; 7 Pelagic Fish Resources Division, Fisheries Stock Assessment Center, Fisheries Resources Institute, Japan Fisheries Research and Education Agency, 1551-8, Taira-machi, Nagasaki, 851-2213 Japan; 8 Highly Migratory Resources Division, Fisheries Stock Assessment Center, Fisheries Resources Institute, Japan Fisheries Research and Education Agency, 5-7-1, Orido, Shimizu, Shizuoka 424-8633 Japan; 9 Department of Oceanography and Coastal Sciences, Louisiana State University, 2255 Energy, Coast and Environment Building, Baton Rouge, LA 70803 USA; 10 Southwest Fisheries Science Center, National Marine Fisheries Service, 8901 La Jolla Shores Drive, La Jolla, CA 92037 USA; 11 Institute of Oceanography, National Taiwan University, Taipei, No. 1, Sec. 4, Roosevelt Rd, Taipei, 10617 Taiwan

**Keywords:** Animal migration, Population dynamics, Ecology, Ecology, Ocean sciences

## Abstract

Geochemical chronologies were constructed from otoliths of adult Pacific bluefin tuna (PBT) to investigate the timing of age-specific egress of juveniles from coastal nurseries in the East China Sea or Sea of Japan to offshore waters of the Pacific Ocean. Element:Ca chronologies were developed for otolith Li, Mg, Mn, Zn, Sr, and Ba, and our assessment focused on the section of the otolith corresponding to the age-0 to age-1 + interval. Next, we applied a common time-series approach to geochemical profiles to identify divergences presumably linked to inshore-offshore migrations. Conspicuous geochemical shifts were detected during the juvenile interval for Mg:Ca, Mn:Ca, and Sr:Ca that were indicative of coastal-offshore transitions or egress generally occurring for individuals approximately 4–6 mo. old, with later departures (6 mo. or older) linked to overwintering being more limited. Changepoints in otolith Ba:Ca profiles were most common in the early age-1 period (ca. 12–16 mo.) and appear associated with entry into upwelling areas such as the California Current Large Marine Ecosystem following trans-Pacific migrations. Natal origin of PBT was also predicted using the early life portion of geochemical profile in relation to a baseline sample comprised of age-0 PBT from the two primary spawning areas in the East China Sea and Sea of Japan. Mixed-stock analysis indicated that the majority (66%) of adult PBT in our sample originated from the East China Sea, but individuals of Sea of Japan origin were also detected in the Ryukyu Archipelago.

## Introduction

Tropical and temperate tunas are essential components of marine ecosystems and play important ecological roles by influencing the structure and dynamics of pelagic food webs^1,2^. Many species of tunas are highly migratory and regularly cross international borders or management boundaries, often traversing oceans to complete their life cycles^3–5^. Migration activities and resulting shifts in spatial distributions of tunas complicate management efforts for targeted populations because fishing pressure varies spatially within an individual’s home range^6^. In response, efforts to better understand movement pathways and the intrinsic drivers that facilitate or initiate spatial shifts are critical, and resource managers readily acknowledge that a species’ migratory history is important to developing reliable population models and management plans^7,8^. While our understanding of the movement ecology of tropical and temperate tunas has increased significantly in the past two decades^9–11^, data on directed migrations during early life when juveniles leave spawning or nursery areas are lacking for many populations and species.

Pacific bluefin tuna (PBT, *Thunnus orientalis*) is distributed throughout the Pacific Ocean^12,13^ and displays wide-ranging movements and population connectivity throughout its range^3,14,15^. Although trans-oceanic movements by PBT are common between eastern and western regions of the Pacific Ocean, spawning is centered in two geographic regions of the western North Pacific Ocean (WNPO): (1) East China Sea between the Philippines, Taiwan, and Ryukyu Archipelago (Nansei Islands) and (2) Sea of Japan. Spawning by PBT occurs earlier in the East China Sea (April–June) relative to the Sea of Japan (July–August)^16,17^. Movement data from electronic tags indicates that a fraction of the age-0 PBT can remain in coastal nurseries until winter, with some individuals overwintering in the East China Sea before heading into offshore waters of the Pacific Ocean^15,18^. While it is well recognized that egress from marginal sea nurseries occurs during the first year of life, the age-specific timing of movements into offshore waters is unresolved.

Geochemical markers in otoliths (ear stones) are increasingly used to retrospectively determine the origin, movement, and population connectivity of tunas^19,20^. In fact, the natal origin for PBT has been predicted based on geochemical signatures in otoliths of sub-adults (corresponds to material deposited during the nursery period) relative to signatures of age-0 individuals (i.e. baseline sample) collected from the East China Sea and Sea of Japan^21^. An extension of this approach is to construct geochemical life history profiles (i.e., chronologies) across the entire otolith (core to margin) to elucidate age-specific patterns of movement between water masses. Although work to date using geochemical chronologies for tunas is limited, findings indicate that the approach shows promise for detecting the timing of spatial shifts or movements into different water masses^22,23^.

Here, geochemical chronologies from otoliths of PBT were developed to retrospectively determine age-specific estimates of egress from coastal to offshore waters of the WNPO. Element:Ca profiles were developed for six markers (Li, Mg, Mn, Zn, Sr, Ba) and our assessment focused on the juvenile stage (age-0 to age-1 + portion of otolith) from adult PBT collected in the waters off the Ryukyu Archipelago in the East China Sea. We then applied changepoint analysis to element:Ca profiles for each individual to identify divergences presumably linked to migrations into offshore waters. Our sample included individuals with a range of predicted birth years to provide a comprehensive assessment of egress into offshore waters in the WNPO from marginal sea nurseries. Given that egress activity results in spatial shifts in the distribution of young PBT in the WNPO and subsequently their exposure to artisanal and commercial fishing pressure, exchanges between coastal and offshore waters are important for understanding the population dynamics of PBT. We also characterized geochemical signatures of age-0 PBT from the two primary spawning areas (East China Sea vs Sea of Japan), and used this baseline signature to determine the natal origin of adult PBT in our sample for assessing contribution rates of migrants from both nurseries.

## Methods

### Sample collection and processing

Adult PBT used in this study (n = 56) were collected from April-June 2017 by the Taiwanese longline fleet operating on spawning grounds in the Ryukyu Archipelago. Specimens were collected from a sampling corridor between 23.0°–25.8° N and 122.0°–125.9° E, and all individuals were deemed to be spawning adults based on their length (mean ± 1 SD: 227.9 ± 21.2 cm FL) and weight (230.8 ± 68.0 kg). Collections of PBT were made under Project 106AS.10.2-AI-A1 from the Council of Agriculture in Taiwan. Age-0 PBT (< 50 cm fork length) used as our baseline sample for predicting natal origin were collected from the two primary spawning areas (East China Sea, Sea of Japan) in 2011–2012. Collections of age-0 PBT from both spawning areas were collected from the commercial fishery by the National Research Institute for the Far Sea Fisheries (Table S1). All collections were performed in accordance with relevant guidelines and regulations of institutional animal care and use committees (IACUC) at Texas A&M University (TAMU). No official animal use license was required by TAMU because biological sampling was performed on harvested (non-living) specimens from commercial fishing operations.

Sagittal otoliths were extracted from both fresh and frozen specimens, cleaned of biological tissue, and stored dry. One sagittal otolith from each PBT in our sample was embedded in Struers EpoFix resin (Struers A/S, Ballerup, Denmark) and sectioned using a low speed ISOMET saw (Buehler, Lake Bluff, Illinois) to obtain 1.5 mm transverse section that included the otolith core following protocols described previously^24^. Otolith thin sections were then attached to petrographic slides using Crystalbond™ thermoplastic glue (SPI Supplies/Structure Probe Inc., West Chester, Pennsylvania). Thin sections were then polished on one side until the center of the primordium was visible while attempting not to reduce thickness below 1 mm.

### Elemental analysis

**Figure 1:**
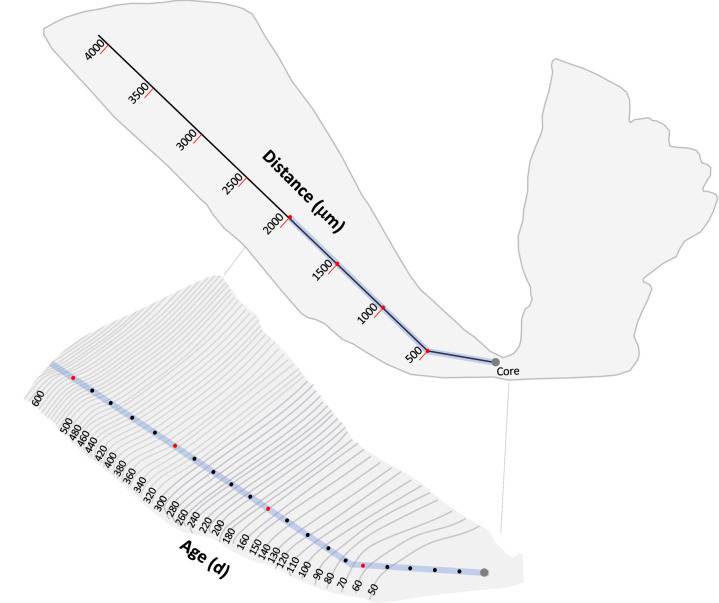
Thin section of Pacific bluefin tuna sagittal otolith displaying location of laser path with green highlighted section of line indicating the portion of the laser scan used to create geochemical profiles (top). Isolated region of otolith thin section corresponding to the geochemical profile along the 2000 µm path was matched to predicted age or days post hatch (dph) from otolith microstructure analysis (bottom). Position of daily increments along the laser scan were estimated for a series of otoliths and grey lines represent estimated age contours at specific intervals (dph).

Elemental concentrations in otoliths of PBT were quantified using an Elemental Scientific NWR193 UC laser ablation (LA) system coupled to an Agilent 7500ce inductively coupled plasma mass spectrometer (ICP-MS) at the University of Texas at Austin. All otoliths and standards processed on the LA-ICP-MS were loaded into a large format cell with fast washout times (< 1 s). Geochemical life history profiles were accomplished by running laser scans from the otolith core or primordium (start of life) outwards along the longest growth axis to the otolith margin (end of life) (Fig. 1). Preliminary scans indicated that optimal ion counts were obtained using gas flows of 850 mL min^−1^ for argon and 800 mL min^−1^ for helium. Prior to analysis, the area of the otolith included in each life history profile (otolith core to edge transect) was pre-ablated to remove surface contamination using an 100 µm spot moving at 50 µm s^−1^, with the laser at 20 Hz and 60% power. Laser parameters during data acquisition was 50% power, 10 Hz with a 50 µm spot moving at 10 µm s^−1^. Elements were quantified on the LA-ICP-MS using the following integration times: 10 ms (^24^Mg, ^43^Ca, ^88^Sr), 20 ms (^25^Mg, ^55^Mn), and 50 ms (^7^Li, ^66^Zn, ^137^Ba).

Time-resolved intensities from the ICP-MS were converted to elemental concentrations (ppm) equivalents using Iolite software with ^43^Ca as the internal standard and a Ca index value of 38.3% weight. Standard baselines were determined from 30 s gas blank intervals measured while the LA system was off, and all masses were scanned by the ICP-MS. The primary calibration standard used was USGS MACS-3, with the accuracy and precision assessed using replicates of NIST 612 as an unknown. NIST 612 analyte recoveries were typically within 2% of GeoREM preferred values (http://georem.mpch-mainz.gwdg.de). Concentrations were then converted to element:Ca molar ratios (R_E_, µmol mol^−1^) based on the molar mass of each element (M_E_, g mol^−1^) and calcium (M_Ca_ = 43 g mol^−1^):

$$R_{E} = \frac{{\left[ E \right]}}{{1000}}\left( {M_{E} \frac{{0.38}}{{M_{{Ca}} }}} \right)^{{ - 1}}$$

### Microstructure analysis

The relationship between LA transect distance from the otolith core and inferred age in days post hatch (dph) was determined using a subset of 25 juvenile (~ age-1 +) PBT collected over several months in 2012 by NOAA’s biological sampling program for recreational tuna caught in the California Current Large Marine Ecosystem^22^. For microstructure analysis of daily increments, thin sections required polishing from both sides to a final thickness of approximately 100–200 μm. Daily increments and 15–20 days isolines were marked with ImagePro (Premier) in calibrated images taken at 400 × magnification with a Nikon Eclipse E400 compound microscope. Age contours were then derived via Kriging (Golden Software Surfer 8.0)^22^ and later used to infer the likely ages along the core-to-edge laser ablation axis (Fig. 1).

**Figure 2:**
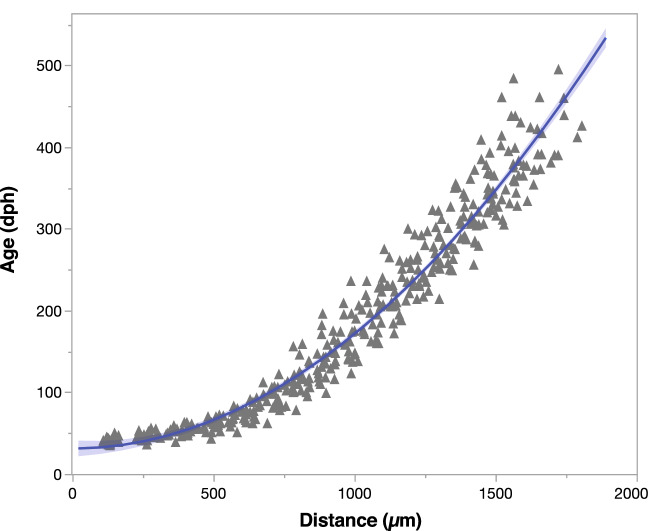
Relationship between distance from otolith core along laser scan and inferred age (days post hatch, dph) developed for 25 yearling Pacific bluefin tuna. Ages were predicted from calibrated composite images of transevers otolith sections from an earlier investigation^22^ with interpolated age contours matched to distance measures at several distances along the laser scan path used in the current study.

Age-specific timing of geochemical shifts along the profile from 0 to 2000 µm (i.e., 0 to ~ 600 + dph) was predicted using a distance from core and age relationship developed from otolith microstructure analysis (Fig. 2). Ages (dph) were estimated along the laser scan path using age contours and matched to distance measures at several distances (~ 15–20) for each individual PBT (Fig. 1). These data were pooled from all individuals to develop the final otolith distance-age relationship used to convert distance along the LA transect to dph and to assign ages to geochemical changepoints. Assigned dph for each distance measure was based on the fitted polynomial quadratic equation:

$${\text{Age}} = 31.2{-}0.001044*{\text{Distance}} + 0.000141*{\text{Distance}}^{2} \left( {{\text{R}}^{2} = 0.954} \right)$$

### Data analysis

Element:Ca ratios used to construct geochemical profiles were smoothed by calculating a moving median derived from a 7-point moving average. We then applied a changepoint analysis, a common time-series approach, to each geochemical life history profile (six element:Ca profiles per individual). Changepoint analysis is a statistical tool for estimating the point at which the statistical property of a time series changes^25^, which we used to objectively identify the location of conspicuous changes in element:Ca ratios along the otolith profile of PBT during the first year of life, when transitions from coastal to offshore water masses occur. The method detected changing points based on mean and variance of each element:Ca profile^26^. Geochemical profiles were treated as time series and constructed from a series of steps, with each step 4.15 µm in distance along the laser scan from the core to 2000 µm (Fig. 1). Although deviations can be detected for single or multiple points in a time series^27^, a constrained approach (singe changepoint) was used here to detect the initial divergence in element:Ca profiles of PBT. This was performed under the assumption that the first significant change in each element:Ca profile measured out from the otolith core (termed as time zero of measurement) represented the starting point of egress (transition from inshore to offshore). Total distance (= total steps) to the point at which the statistical properties of the geochemical profile changed was used to predict the timing (age) of each individual making the inshore-offshore transition. Changepoints were reported as distances from core along geochemical profiles (i.e., laser path), and changepoints were determined separately for each element:Ca ratio for each individual. To meet the assumption of stationary time series under the null hypothesis for the changepoint analysis, Kwiatkowski-Phillips-Schmidt-Shin stationary (KPSS) test was conducted for each time series available in an R package (tseries). KPSS tests showed that all time series were stationary at a *p*-value of 0.05. Analysis of variance (ANOVA) and Tukey’s honestly significant difference (HSD) tests were used to determine whether age at egress estimates for PBT from changepoint analysis differed among the six element:Ca ratios.

Natal origin of adult PBT from the Ryukyu Archipelago in the East China Sea was determined with a maximum likelihood estimator from a widely applied mixed-stock technique (HISEA)^28^. Canonical discriminant analysis (CDA) was used to display multivariate means of otolith element:Ca ratios of age-0 PBT, and discriminant function coefficients were incorporated into CDA plots as vectors from a grand mean to show the discriminatory influence of each marker on discrimination to East China Sea and Sea of Japan spawning/nursery areas. Multivariate analysis of variance (MANOVA) was used to test whether element:Ca signatures of age-0 PBT differed between the East China Sea and Sea of Japan. In addition, univariate contrasts (ANOVAs) were performed on individual element:Ca ratios to assist in the identification of the most influential markers for discrimination between the two regions. Quadratic discriminant function analysis (QDFA) was then used to determine the classification accuracy of otolith element:Ca signatures for assigning individuals to the two spawning/nursery areas. To align otolith geochemical signatures of adults during the age-0 interval to signatures of age-0 specimens used in the baseline sample, we limited element:Ca data to the first 500 µm of the geochemical profile (~ first two months of life). Age-class matching (birth year of age-0 fish in baseline with birth year of adults) is preferred due to interannual variation in baseline signatures of tuna in the Pacific Ocean^29,30^. An established age-length key for PBT indicated that only about 40% of adults in our sample were from birth years within 0–3 years of the baseline (Table S2), and therefore, many adults were from birth years several years before 2011. The lack of early baseline data is a common logistical constraint for mixed-stock analysis performed on adults of longer-lived fishes and clearly increases the uncertainty of assignments to spawning areas or stocks^11,19^. Moreover, there is the potential for unsampled sources to influence mixed-stock prediction; however, element:Ca ratios of age-0 (baseline) and adult PBT were plotted in ordination space and the high degree of overlap, suggesting that adult PBT in our sample were likely derived from either the East China Sea and Sea of Japan. The baseline applied here combined two years of age-0 PBT from both spawning/nursery areas and therefore mixed-stock predictions are based on a reference sample that incorporates some degree of interannual variability. Mixed-stock analysis included bootstrapping with 1000 simulations to obtain estimates of uncertainty around estimated proportions.

## Results

### Geochemical life history profiles

**Figure 3:**
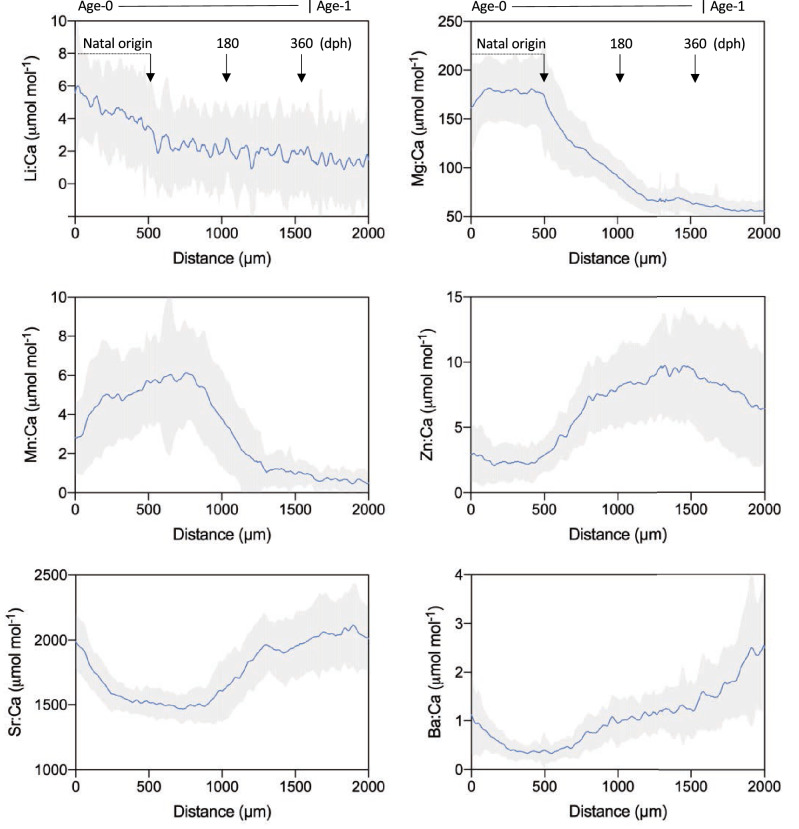
Mean geochemical profiles developed using element:Ca data from all Pacific bluefin tuna collected from the Ryukyu Archipelago in the East China Sea (N = 56, Table S2). Mean profiles shown separately for each of the six element:Ca ratios (Li:Ca, Mg:Ca,m Mg:Ca, Zn:Ca, Sr:Ca, Ba:Ca) along the laser path from the otolith core to 2000 µm. Blue lines represent mean values and shading denotes 1 SD. Area of laser path used to estimate natal origin (0–500 µm) is denoted; location on geochemical profile corresponding to 180 and 360 days post hatch (dph) is also provided.

Otolith element:Ca profiles based on data from all PBT (data pooled, n = 56) displayed noticeable ontogenetic shifts for several of the elemental markers analyzed over the first 2000 µm of the laser transect (region corresponds to age-0 to age-1 period of otolith). Mean otolith Mg:Ca and Mn:Ca ratios were highest during the early age-0 period (< 800 µm distance) for both element:Ca ratios, then decreased until approximately 1000 µm; both maintained relatively low values for the remainder of the geochemical profile (Fig. 3). In contrast, otolith Zn:Ca, Sr:Ca and Ba:Ca ratios of PBT increased as distance from the core increased with the lowest values generally observed during the first 1000 µm of the transect. Marked increases in otolith Zn:Ca and Sr:Ca ratios of PBT were present at distance of approximately 800 µm and 1200 µm from the core, respectively, while Ba:Ca ratios started rapidly increasing later at a distance of approximately 1500 µm or more from the core (Fig. 3); both otolith Sr:Ca and Ba:Ca ratios remained elevated during the remainder of the geochemical profile to 2000 µm or well into the age-1 period (up to ~ 600 dph) (Fig. 1). Mean otolith Li:Ca ratios decreased with distance from core, although the trend was not distinct.

**Figure 4:**
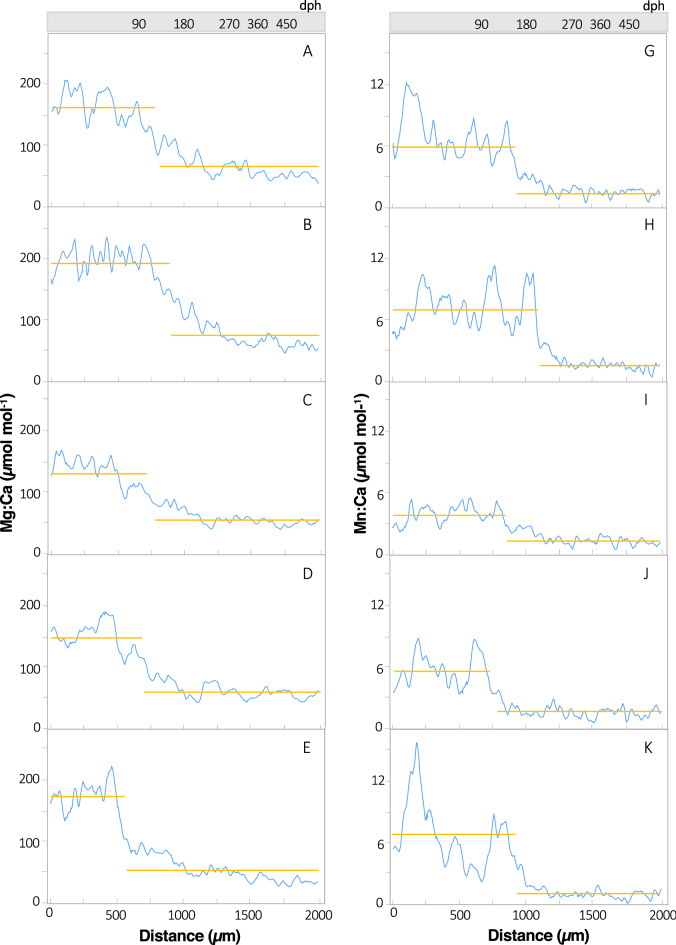
Otolith Mg:Ca (left, panels **A**–**E**) and otolith Mn:Ca (right, panels **G**–**K**) profiles for five individual Pacific bluefin tuna. Identification numbers from top to bottom: 103 (**A**, **G**), 210 (**B**, **H**), 221 (**C**, **I**), 271 (**D**, **J**), 463 (**E**, **K**); Table S2. Blue lines represent 7-point moving average and the location of geochemical changepoints along the laser path from otolith core to 2000 µm are shown as breaks in the horizontal lines (gold). Estimates of age isolines corresponding to distance along the laser path are also provided.

**Figure 5:**
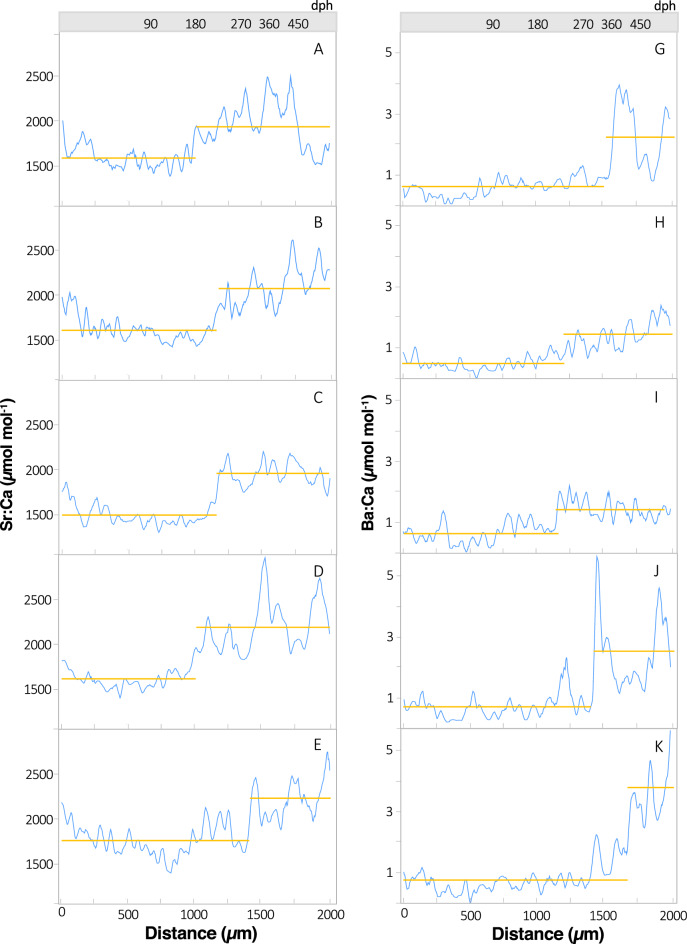
Otolith Sr:Ca (left, panels **A**–**E**)) and otolith Ba:Ca (right, panels **G**–**K**) profiles for five individual Pacific bluefin tuna. Identification numbers from top to bottom: 103 (**A**, **G**), 210 (**B**, **H**), 221 (**C**, **I**), 271 (**D**, **J**), 463 (**E**, **K**); Table S2. Blue lines represent 7-point moving average and the location of geochemical changepoints along the laser path from otolith core to 2000 µm are shown as breaks in the horizontal lines (gold). Estimates of age isolines corresponding to distance along the laser path are also provided.

Individual variation in otolith distance or predicted age of geochemical shifts for PBT (n = 56; Table S2) was present within each of the four element:Ca profiles evaluated; nevertheless, element-specific trends were also clearly evident (Fig. 4, Fig. S1). Mean changepoints across individuals differed among Mg:Ca, Mn:Ca, Sr:Ca, and Ba:Ca (ANOVA, *p* < 0.01), with changepoints detected earlier in Mg:Ca (mean ± 1 SD: 728 ± 158 µ distance, 109 ± 34 dph) and Mn:Ca (1020 ± 158 µ distance, 183 ± 46 dph) profiles (Fig. 4, Fig. S1). Ontogenetic shifts in otolith Sr:Ca profiles were detected later during the age-0 period of PBT and displayed more variability (mean: 1207 ± 297 µ distance, age 252 ± 90 dph) than both Mg:Ca and Mn:Ca profiles (Fig. 5, Fig. S1). Changepoints detected for otolith Ba:Ca profiles of PBT occurred at significantly greater distances from the core (i.e. older ages; Tukey HSD, *p* < 0.05) and were the most variable (mean: 1452 ± 352 µ distance, 344 ± 137 dph). In fact, changepoints of several individuals were beyond 1700 µm distance along the transect or well into the age-1 period (> 400 dph) (Fig. 5, Fig. S1). Coefficent of varation (CV) for changepoints derived from Li:Ca and Zn:Ca profiles were highly variable (CVs 94% and 66%, respectively) relative to the other markers (CVs ~ 20–30%) and not used for multielemental estimates of changepoints (Fig. S1).

**Figure 6:**
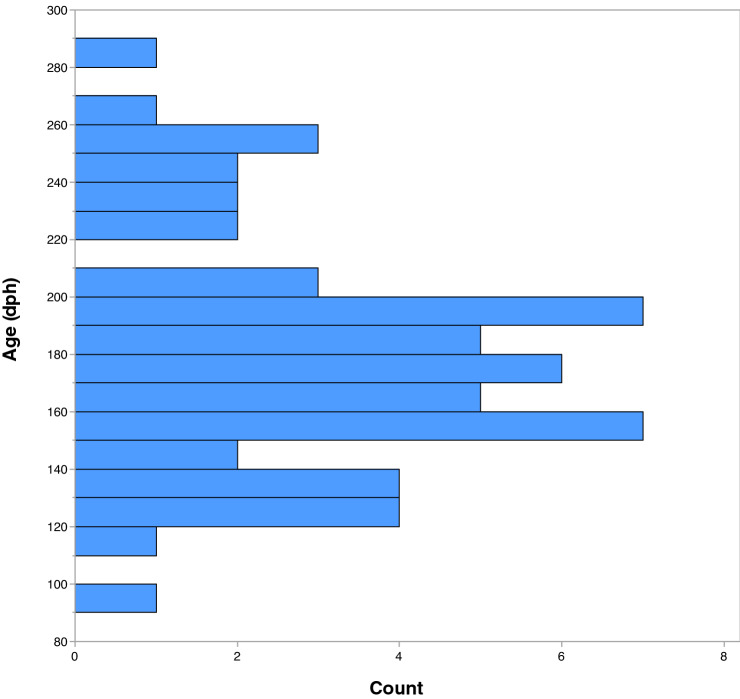
Age-frequency distribution of geochemical changepoints for Pacific bluefin tuna detected using otolith Mg:Ca, Mg:Ca, and Sr:Ba. Estimated changepoint age of each individual was based on average from all three markers.

Given that otolith Mg:Ca, Mn:Ca, and Sr:Ca profiles may reflect environmental or physiological drivers differently and all three markers displayed shifts during the age-0 period of PBT, distance to multielemental changepoints was determined for each individual. Average age of changepoints using these three markers was 180 ± 42 dph, and the distribution of ages based on averaged changepoints ranged from 93 to 281 dph (Fig. 6). The majority of multielemental changepoints for PBT in our sample were between approximately 120–200 dph, with only a few individuals in our sample displaying shifts (i.e. egress) earlier than 120 dph. We also observed that changepoints for about 20% of our sample occurred well beyond six months (~ 220–290 dph), suggesting that a fraction of PBT in our sample remained in the marginal sea nursery into the late winter or early spring (based on presumed spawning times for this species).

### Natal origin and mixing

**Figure 7:**
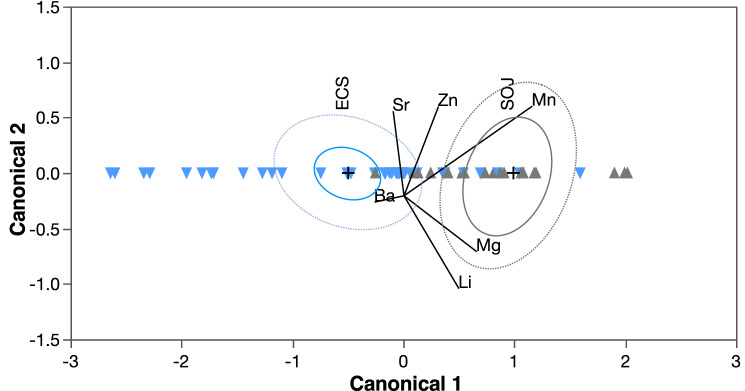
Canonical discriminant analysis based on otolith element:Ca ratios of age-0 Pacific bluefin tuna collected from the East China Sea (ECS = blue) and Sea of Japan (SOJ = grey). 95% confidence ellipse (solid line) around each centroid denoted with + symbol and normal 50% ellipse (dashed line) shown. Since classification variable has only two levels, points are plotted against the single canonical variable (Canonical 1) and canonical weights for each covariate relate to this variable only. Biplot vectors from each centroid indicate the influence of each element:Ca ratio (Li:Ca, Mg:Ca,m Mg:Ca, Zn:Ca, Sr:Ca, Ba:Ca) on regional discrimination. Rays shown with a vertical component only for improved separation but all are relative to Canonical 1 axis.

Quadratic discriminant function analysis parameterized with otolith Li:Ca, Mg:Ca, Mn:Ca, Zn:Ca, Sr:Ca, Ba:Ca ratios from age-0 PBT collected in 2011 and 2012 showed that geochemical signatures for individuals collected in the East China Sea and Sea of Japan were statistically distinct (MANOVA, *p* < 0.01). Univariate contrasts of three element:Ca ratios (Li:Ca, Mg:Ca, Mn:Ca) were significantly different between the two regions (ANOVAs, *p* < 0.01), and CDA also indicated that Li:Ca, Mg:Ca and Mn:Ca were the most discerning markers for predicting natal origin (Fig. 7). Mean values for all three element:Ca ratios were higher for age-0 PBT from the Sea of Japan compared to the East China Sea. QDFA cross-validated classification success of age-0 PBT to the two nurseries in 2011–2012 was 87%, indicating the approach was suitable for predicting natal origin of adult PBT. Maximum likelihood estimation of natal origin predicted that most of the adult PBT in our sample (66.1% ± 10.2%) originated from the same area; however, individuals of Sea of Japan origin (33.9% ± 10.2%) were also detected in this region.

## Discussion

Conspicuous changes in geochemical chronologies were present for PBT during the first year of life, and changepoint analysis served as a novel approach for identifying divergences along otolith-based life history profiles. Abrupt shifts in otolith element:Ca ratios are often associated with movements between water masses with different physicochemical properties^31,32^. Given that physicochemical conditions such as salinity and temperature as well as metal loads in coastal waters are often distinct relative to offshore waters of the WNPO and associated Kuroshio Current^33,34^, divergences observed along element:Ca profiles likely predict the timing of egress or ontogenetic changes linked to the movement by young PBT from inshore nurseries to offshore waters. In addition to extrinsic influences, shifts in otolith element:Ca ratios of PBT will also be related to intrinsic (e.g., physiological, genetics) factors that may be directly or indirectly linked to ontogenetic transitions from inshore to offshore habitats, suggesting that geochemical changepoints observed for PBT may reflect both environmental and physiological changes^35^.

Observed patterns of decreasing otolith Mg:Ca and Mn:Ca ratios present for PBT during the first year of life are in accord with expected timing of migrations from coastal nurseries to offshore waters of the Pacific Ocean^17^. Seawater Mg:Ca and Mn:Ca ratios in marginal seas and coastal areas influenced by rivers are semi-conservative to non-conservative, and upper ranges of observed ratios and associated variability (range) are higher in coastal or river-influence margins compared to offshore waters, potentially leading to inshore-offshore differences^36,37^. In the WNPO, surface waters are impacted by the western boundary current (Kuroshio Current) and mesoscale features that are reduced in metals^38^, further supporting this aforementioned inshore-offshore depletion gradient of both markers. Still, it is important to note that otolith geochemistry cannot always be consistently linked to ambient Mg:Ca and Mn:Ca in seawater^39,40^. Apart from ambient seawater chemistry, salinity and temperature may play a role in observed shifts in otolith Mg:Ca and Mn:Ca, but salient shifts in these markers appear to be driven primarily by changes in somatic growth rather than salinity and/or temperature^41,42^. While the contribution of each driver to observed patterns in otolith Mg:Ca and Mn:Ca is unresolved and may be partially linked to other factors associated with the inshore-offshore transition (e.g., metabolism, diet, growth), observed divergences in geochemical profiles and the timing of such changes align well with the expected migrations of young PBT from coastal nurseries to foraging areas in offshore waters.

In contrast to otolith Mg:Ca and Mn:Ca, a pronounced increase in otolith Sr:Ca occurred during the age-0 period. Positive relationships between otolith Sr:Ca and salinity have been reported for a wide range of species, with otolith Sr:Ca commonly occurring at proportions relative to ambient levels^42,43^. This element:Ca ratio has proven useful for documenting shifts—both ingress and egress—between waters masses with distinct salinity values^41,44^. Although Sr and Ca in seawater are conserved at higher salinity, resulting in less Sr: Ca variation in water across marine salinity gradients^45,46^, this marker has been used to effectively delineate inshore-offshore transitions for several species^47,48^. Observed Sr:Ca profiles for PBT during the first year of life displayed marked increases in otolith Sr:Ca later during the age-0 period. This finding is in agreement with anticipated egress of young PBT from inshore to offshore waters of the WNPO, which are characterized by higher salinity relative to coastal waters in the East China Sea or Sea of Japan. Physiological influences on otolith composition are particularly evident for certain element:Ca ratios (Sr:Ca)^46,49,50^, and physiological changes associated with inshore-offshore ontogenetic transitions of young PBT may have contributed to observed shifts in otolith Sr:Ca. Moreover, physiological controls may be moderated by changes in ambient water temperature also occurring during the transition, which can also have a positive influence on otolith Sr:Ca^51,52^ and movement into the warmer, oligotrophic waters in the Kuroshio Current^53^ or farther offshore into the WNPO may also be responsible for pronounced increases in otolith Sr:Ca often observed after the first 6–8 months of life.

The timing (age) of geochemical changepoints used to predict departures of age-0 PBT from coastal nurseries often varied among markers (Mg:Ca, Mn:Ca, and Sr:Ca) for individual PBT. Disparities in mean ages associated with shifts in element:Ca profiles were evident with geochemical changepoints presumably linked to egress and detected about three months earlier in otolith Mg:Ca relative to Sr:Ca, with Mn:Ca being intermediate to the these two markers. Despite the disparity of presumed egress ages, the earliest estimated departure times were generally greater than 90 dph for all three markers, suggesting that most recruits remain in coastal nurseries for at least the first three to four months of life. Given that otolith Mg:Ca, Mn:Ca, and Sr:Ca ratios may reflect and/or respond (i.e., lag effect) differently to changing environmental or physiological conditions^38,46^, combining multiple markers often leads to more conservative estimates of age-specific egress or ingress^22^. Changepoints derived from all three of these markers integrate sensitivities of each and were used to produce a more robust indicator of presumed egress or geochemical shifts by PBT. Using this approach, the majority of geochemical changepoints were detected at 150–200 dph, suggesting that age-0 PBT in our sample inhabited coastal waters throughout the summer and into the fall, taking advantage of elevated primary productivity found here relative to more depleted conditions in offshore waters of the Kuroshio Current^54^. Our findings are in accord with an archival tagging study that also observed young PBT inhabiting coastal waters inshore of the Kuroshio Current during the summer and fall^17^. Changepoints in element:Ca profiles for nearly a quarter of the PBT in our sample did not occur until after 200 dph, suggesting these individuals may overwinter in coastal nurseries before moving offshore in the early spring. Overwintering behavior by age-0 PBT from both the East China Sea and Sea of Japan is known to occur in the East China Sea^55,56^, supporting our finding of a delayed geochemical shift or late egress for some individuals.

Observed variability in the age-at-egress among individual PBT in our sample using average estimates from Mg:Ca, Mn:Ca, and Sr:Ca (93–281 dph) is not entirely unexpected given that the birth year (age-0 period) of individuals in our samples spanned many years (Table S1; using age-length relationship derived from previous study^57^). Interannual variation in coastal and oceanographic conditions are common in the WNPO and strongly influenced by the dynamics of the Kuroshio Current^52^. The path and coastal intrusion of the Kuroshio Current varies seasonally and annually in the Ryukyu Archipelago and off the east coast of Japan^34^. Inshore-offshore fluctuations in the Kuroshio Current and associated mesoscale features (e.g., large meander) commonly occur and are known to influence the spatial distribution of prey^58,59^. Moreover, the position of the Kuroshio Current relative the coastline also influences the habitat use and movement of age-0 PBT between inshore and offshore waters^17,56,60^. More specifically, advection of waters away from the coast due to the current and associated eddies, often in the winter and spring, results in a wider distribution of age-0 PBT and may facilitate fish moving to offshore waters in the Kuroshio-Oyashi Transition Zone (KOTZ)^17^. Interannual variation in the pathway and westward penetration of the Kuroshio Current and its subsequent influence on habitat use (inshore vs. offshore water) by young PBT combined with the fact that birth years of adult PBT in our sample spanned more than five years contributed to observed variation in the timing of predicted egress using geochemical changepoints.

Otolith Ba:Ca profiles of PBT were relatively consistent during the first year of life, and changepoints for individuals in our sample were often not detected until that age-1 period. This geochemical marker was presumably uninformative for detecting inshore-offshore transitions by age-0 PBT; nevertheless, conspicuous shifts were detected in the late age-0 to early age-1 period for PBT in our sample, and these geochemical divergences may be related to a different life history transition. Because Ba:Ca in seawater is enriched at depth, upwelling elevates Ba:Ca ratios in surface waters^60^, which in turn is often reflected in elevated otolith Ba:Ca^22,31^. Consequently, this marker shows promise for signifying entry into upwelling zones by PBT^22^ and other taxa (e.g., sharks^61^). Highly productive waters of both the KOTZ in the WNPO and the California Current Large Marine Ecosystem (CCLME) in the eastern North Pacific Ocean represent two areas with strong upwelling and subsequently elevated seawater Ba:Ca^62^. Elevated primary and secondary productivity occurs along frontal boundaries of both features, which represent critical foraging habitat of young PBT^16,18^. As a result, entry into associated upwelling zones of the KOTZ or CCLME by PBT is expected to result in conspicuous increases in otolith Ba:Ca. The majority (70%) of changepoints detected in otolith Ba:Ca profiles of individual PBT were between 10 and 18 months (mean: 344 dph). Interestingly, archival tagging showed that the timing of departures by juveniles from the WNPO in the vicinity of the KOTZ begins at around 12 months with average transit times about 2.5 months^15^. Therefore, elevated otolith Ba:Ca first observed during the late age-0 or early age-1 period may be linked to their occurrence in highly productive waters of the KOTZ or associated upwelling areas along the margin of the Kuroshio Current^63^ while elevated values and resulting changepoints detected later in the age-1 period (~ 14–18 months) may signify movement into the highly productive waters of the CCLME^22^.

Element:Ca signatures in the otoliths of age-0 PBT in the 2011–2012 sample from East China Sea and Sea of Japan were significantly different, leading to relatively high (87%) classification success. Influential element:Ca ratios in otoliths for discriminating PBT from both regions (Mg:Ca, Mn:Ca, Li:Ca) were consistent with earlier studies^21,29^, suggesting that long-term differences in physicochemical conditions may persist between both spawning areas. While the promise of using these geochemical markers for mixed-stock analysis of PBT is evident, it is important to note that insufficient matching of birth years of adults to our age-0 baseline as well as the potential for additional spawning and/or nursery areas, including regions near or within the Kuroshio–Oyashio transition area^64^, are potential sources of uncertainty in our mixed-stock assignments. Still, predictions of natal origin reported here shed important light on the origin of adult PBT collected in the East China Sea, and our findings indicated that spawning adults caught in Taiwan fisheries off the Ryukyu Islands were predominantly from the East China Sea spawning area (~ 2/3), suggestive of site fidelity to this region. The presence of adult PBT with core signatures matching the Sea of Japan were also observed in our sample, implying that waters in the Ryukyu Archipelago also represent a potential mixing zone for individuals produced from both spawning areas^65^. Similarly, the presence of adults from both spawning areas (i.e., mixing) was recently reported in collections from the Sea of Japan and Nansei Islands using natal origin estimates derived from widths of the first vertebral annulus^66^. Daily geolocation estimates from electronic tags have also shown individual tracks of PBT moving between the East China Sea and Sea of Japan^67^, suggesting that the Ryukyu Archipelago may be an important foraging area that serves as a mixing zone for migrants from both spawning areas.

Changepoint analysis of geochemical profiles represents a promising methodology for elucidating habitat shifts or movements of juvenile PBT, including coastal-offshore transitions that occur during the first year of life. Our investigation sheds important light on age-specific migrations of juvenile PBT between coastal and offshore waters, and this research suggests that changepoint analysis of element:Ca chronologies may prove useful for detecting habitat transitions by older, larger PBT as well as other species. A distinct advantage of this approach over other widely used techniques to investigate animal movements (e.g., acoustic and satellite telemetry) is that it allows for retrospective determination of natal origin as well as age-specific estimate of movement by coupling highly resolved (interval ~ daily) otolith microstructure and geochemical data. Moving forward, synthesis of data from multiple techniques, including otolith geochemical chronologies, will be required to fully understand the complex nature of migrations by PBT.

## Supplementary information

Supplementary Information 1.

Supplementary Information 2.
