# Supplementary material for: Natal origin and age-specific egress of Pacific bluefin tuna from coastal nurseries revealed with geochemical markers
Source: Sci Rep. 2021 Jul 9;11:14216. doi: 10.1038/s41598-021-93298-2 (PMC8270904; doi:10.1038/s41598-021-93298-2)
Supplement: Supplementary file 1 — Supplementary Information 1. [file 41598_2021_93298_MOESM1_ESM.pdf]

Natal origin and age-specific egress of Pacific bluefin tuna from coastal nurseries revealed with geochemical markers

Jay R. Rooker[1,2*], R. J. David Wells[1,2], Barbara A. Block[3], Hui Liu[1], Hannes Baumann[4], Wei-Chuan Chiang[5], Michelle Sluis Zapp[1], Nathaniel R. Miller[6], John A. Mohan[1], Seiji Ohshimo[7], Yosuke Tanaka[8], Michael A. Dance[9], Heidi Dewar[10], Owyn E. Snodgrass[10], Jen-Chieh Shiao[11]

[1]Department of Marine Biology, Texas A&M University at Galveston, 200 Seawolf Parkway, Galveston, TX 77553, USA

[2]Department of Ecology and Conservation Biology, Texas A&M University, College Station, TX 77843-2258

[3]Hopkins Marine Station, Stanford University, 120 Oceanview Boulevard, Pacific Grove, CA 93950 USA

[4]Department of Marine Sciences, University of Connecticut, 1080 Shennecossett Road, Groton, CT 06340-6048, USA

[5]Council of Agriculture, Fisheries Research Institute TW, 199 Hou-Ih Road, Keelung, 20246, Taiwan

[6]Jackson School of Geosciences, The University of Texas at Austin, 2275 Speedway Stop C9000, Austin, TX 78712, USA

[7]Pelagic Fish Resources Division, Fisheries Stock Assessment Center, Fisheries Resources Institute, Japan Fisheries Research and Education Agency 1551-8, Taira-machi, Nagasaki, 851-2213 Japan

[8]Highly Migratory Resources Division, Fisheries Stock Assessment Center, Fisheries Resources Institute, Japan Fisheries Research and Education Agency 5-7-1, Orido, Shimizu, Shizuoka, 424-8633, Japan

[9]Department of Oceanography and Coastal Sciences, Louisiana State University, 2255 Energy, Coast and Environment Building Baton Rouge, Louisiana 70803, USA

[10]Southwest Fisheries Science Center, National Marine Fisheries Service, 8901 La Jolla Shores Drive, La Jolla, CA 92037, USA

[11]Institute of Oceanography, National Taiwan University, Taipei, No. 1, Sec. 4, Roosevelt Rd, Taipei 10617, Taiwan

Figure S1. Otolith Li:Ca, Mg:Ca, Mn:Ca, Zn:Ca, Sr:Ca, and Ba:Ca profiles for 56 adult Pacific bluefin tuna (Identification numbers displayed). Blue lines represent 7-point moving average and the location of geochemical changepoints along the laser path from otolith core to 2000 μm are shown as breaks in the horizontal lines.

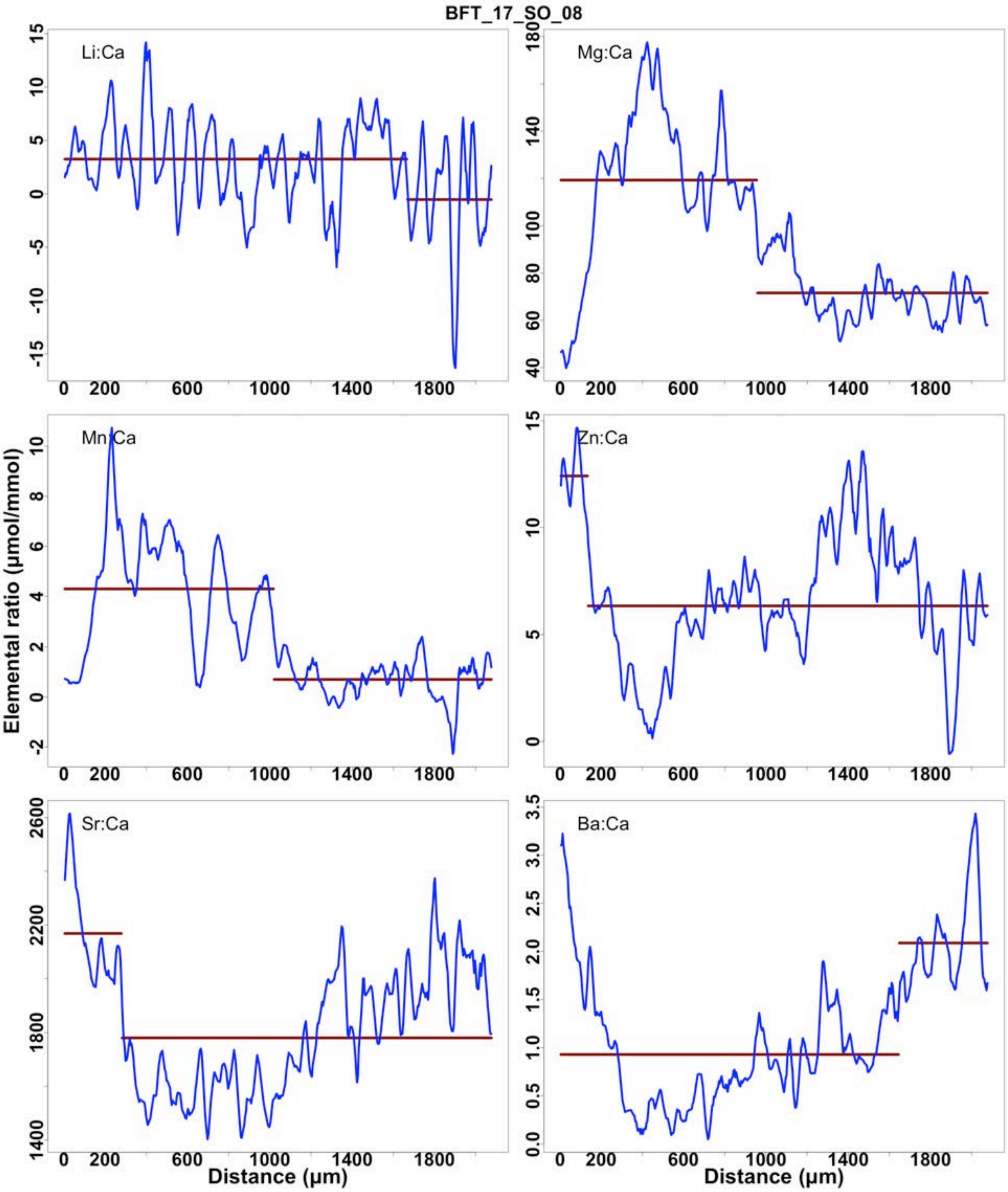

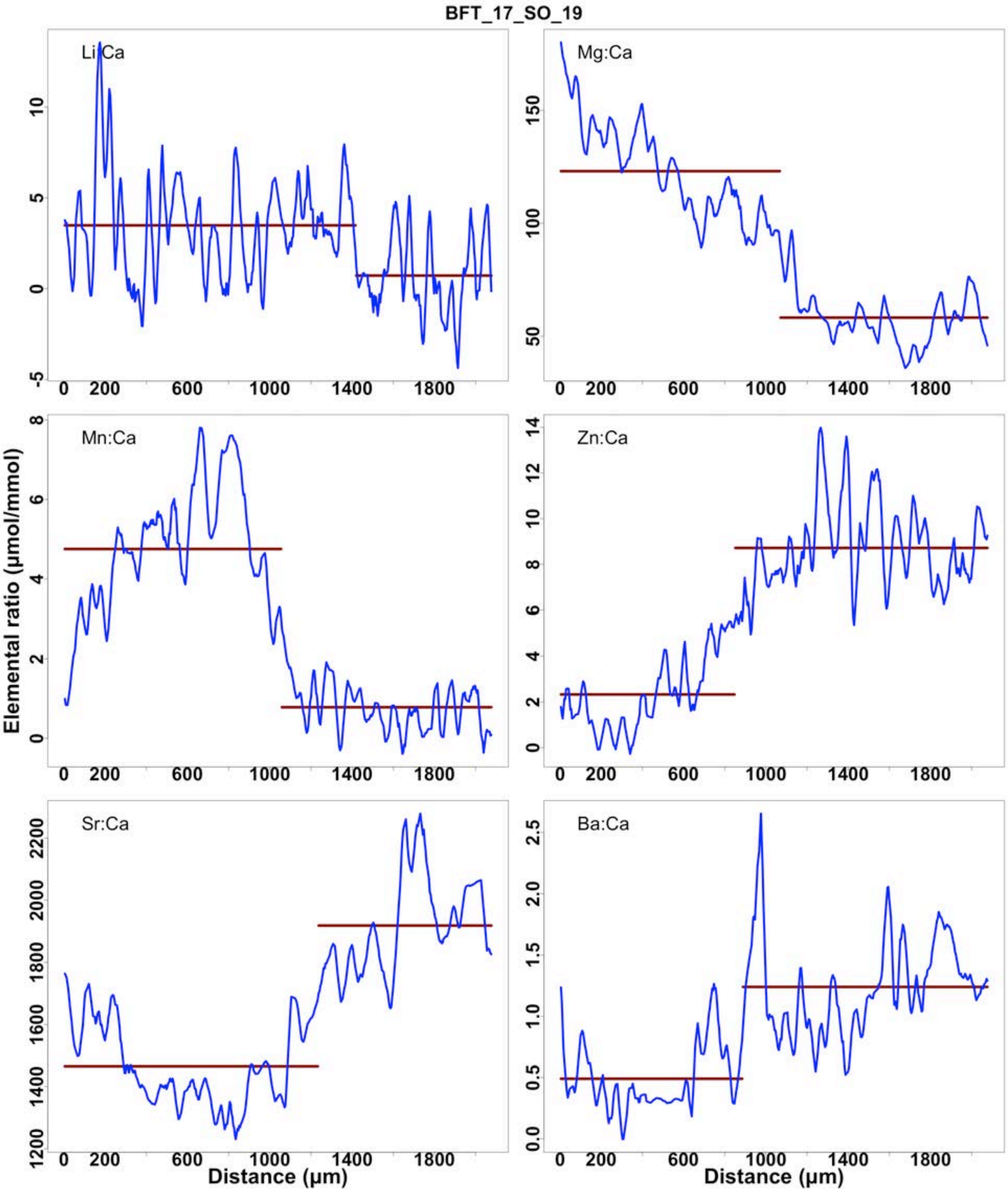

BFT_17_SO_19
Li:Ca
Mg:Ca
Mn:Ca
Zn:Ca
Sr:Ca
Ba:Ca
Elemental ratio (μmol/mmol)
Distance (μm)
Distance (μm)

BFT_17_SO_27

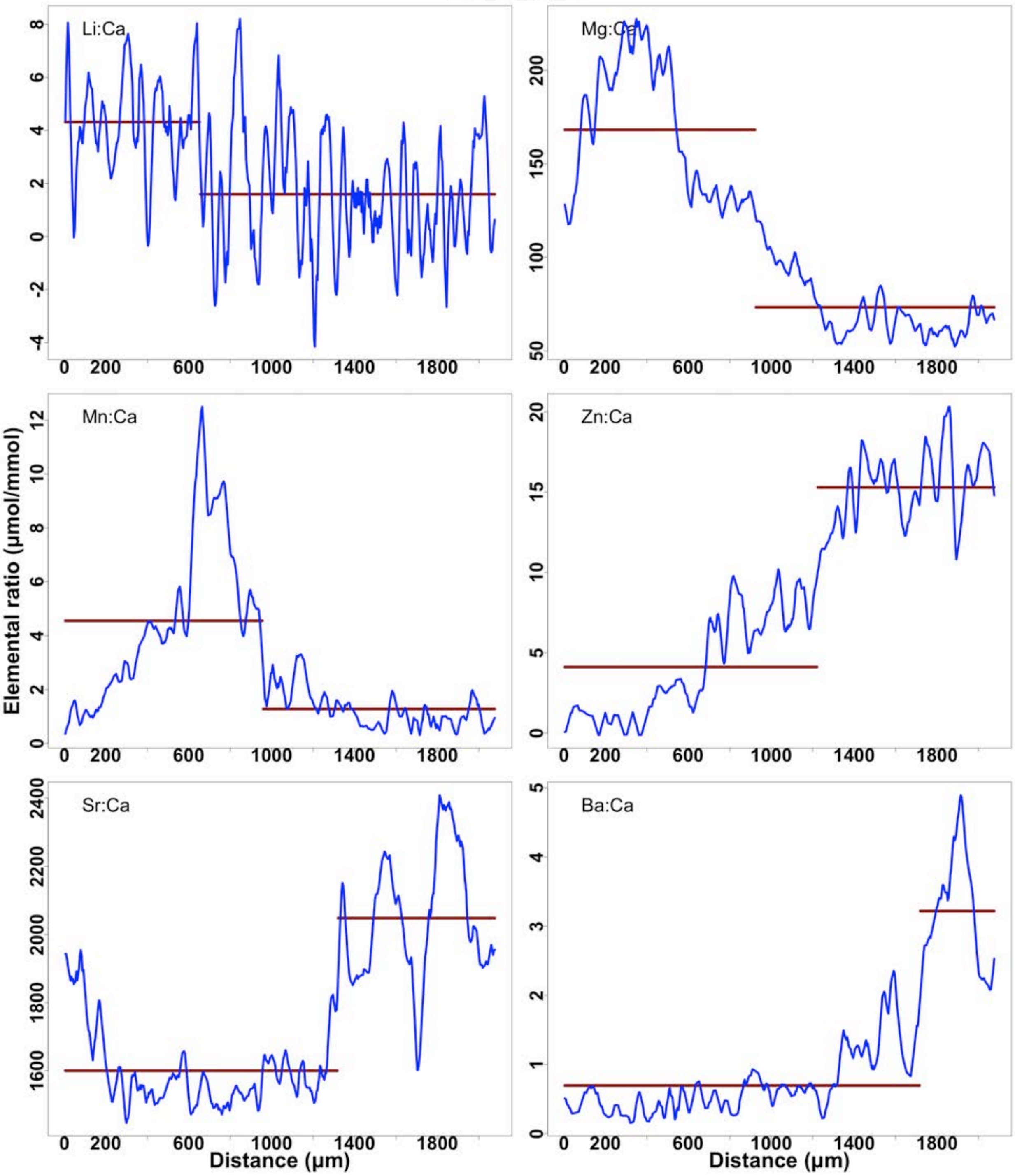
Li:Ca
Mg:Ca
Mn:Ca
Zn:Ca
Sr:Ca
Ba:Ca
Elemental ratio (μmol/mmol)
Distance (μm)
Distance (μm)

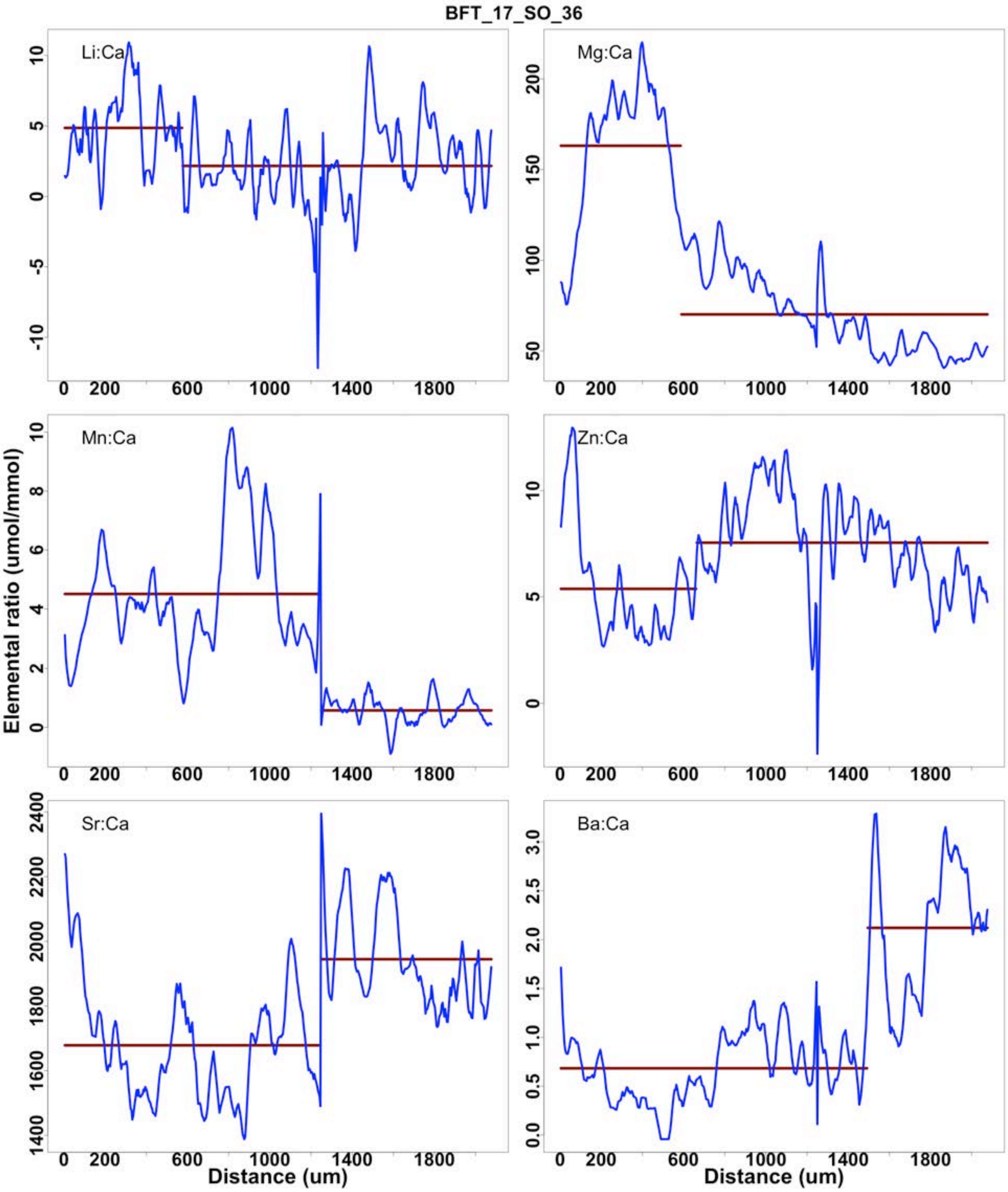
BFT_17_SO_36
Li:Ca
Mg:Ca
Mn:Ca
Zn:Ca
Sr:Ca
Ba:Ca
Elemental ratio (umol/mmol)
Distance (um)
Distance (um)

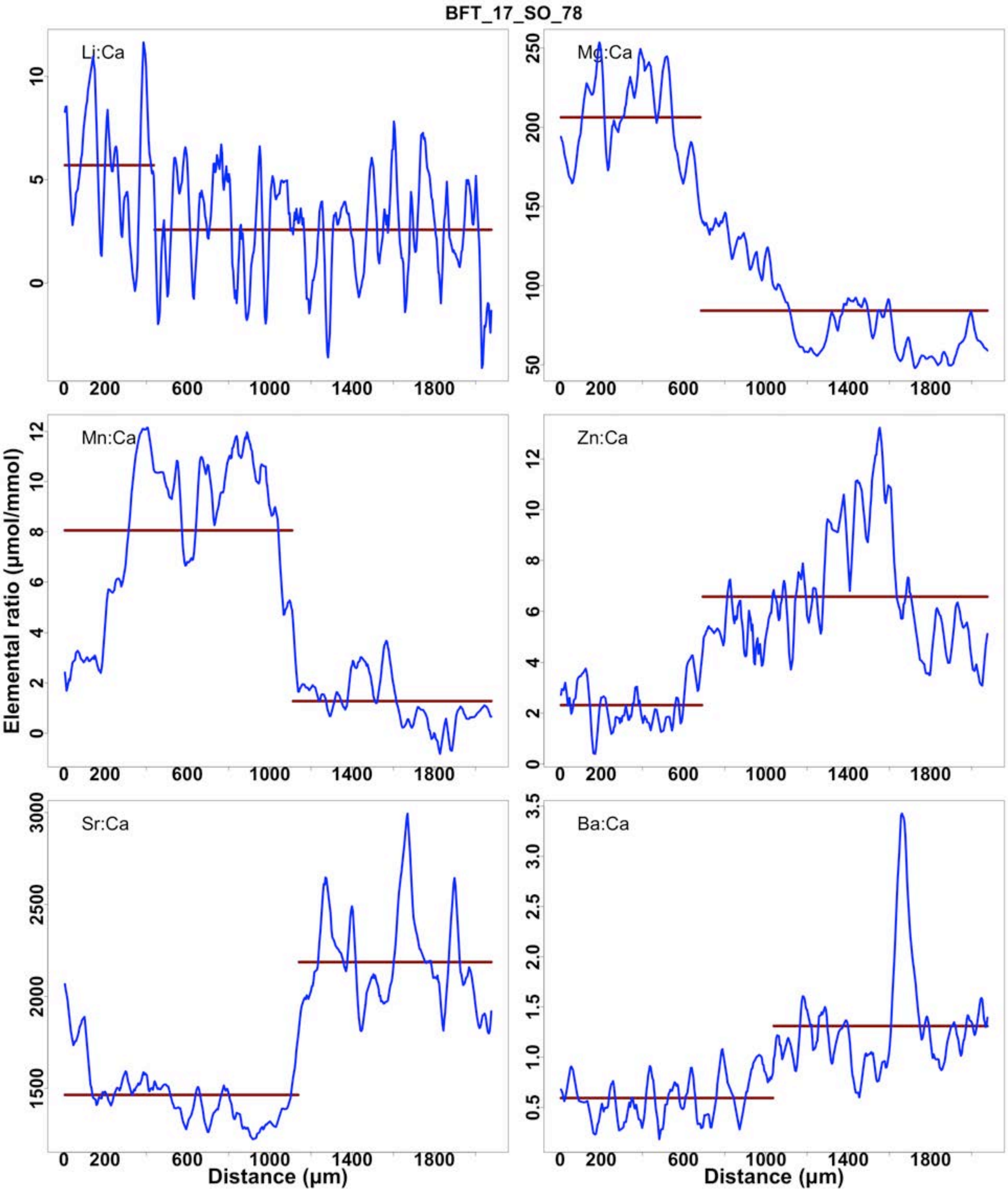
BFT_17_SO_78
Li:Ca
Mg:Ca
Mn:Ca
Zn:Ca
Sr:Ca
Ba:Ca
Elemental ratio (µmol/mmol)
Distance (µm)
Distance (µm)

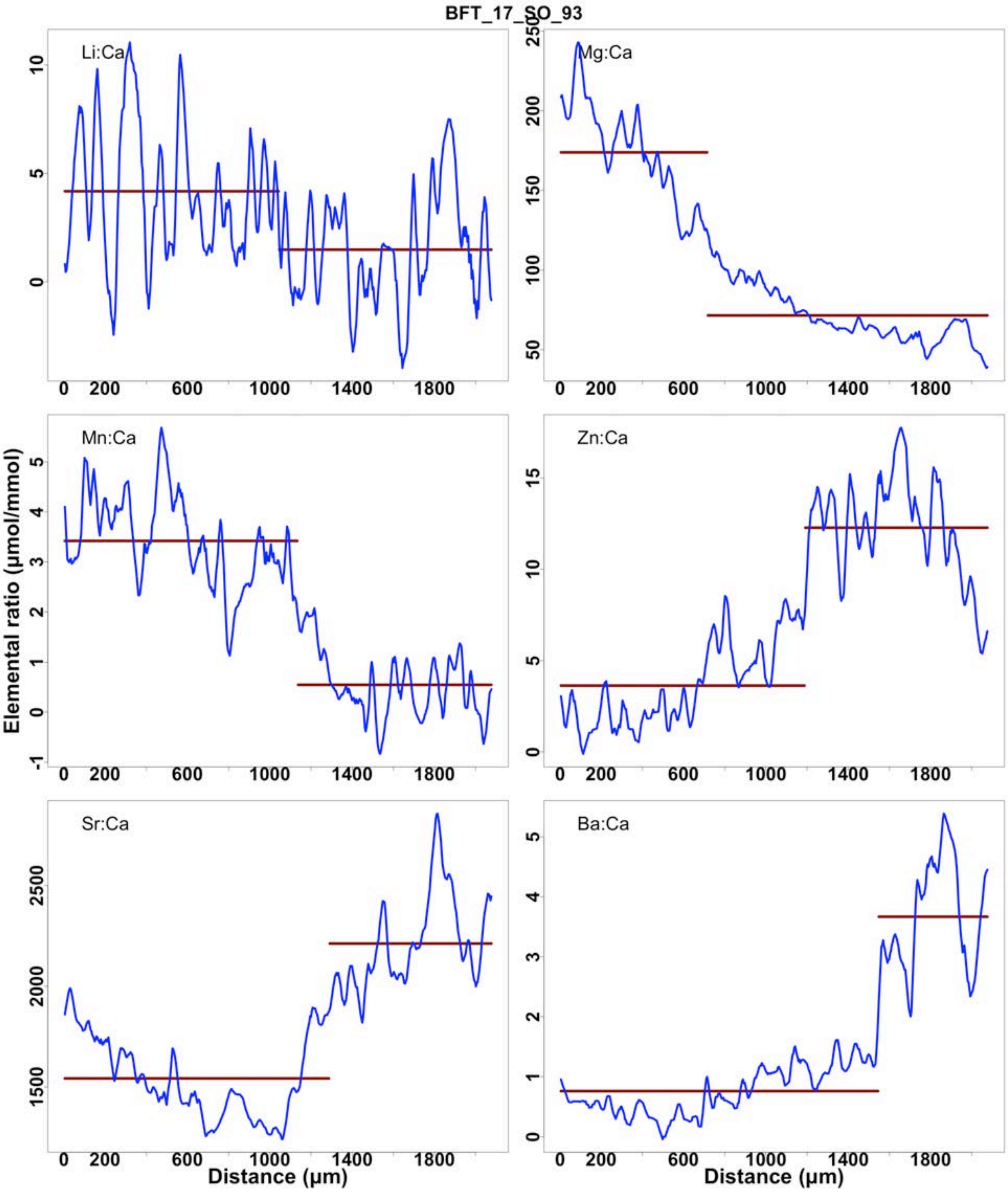

BFT_17_SO_93
Li:Ca
Mg:Ca
Mn:Ca
Zn:Ca
Sr:Ca
Ba:Ca
Elemental ratio (µmol/mmol)
Distance (µm)
Distance (µm)

BFT_17_SO_103

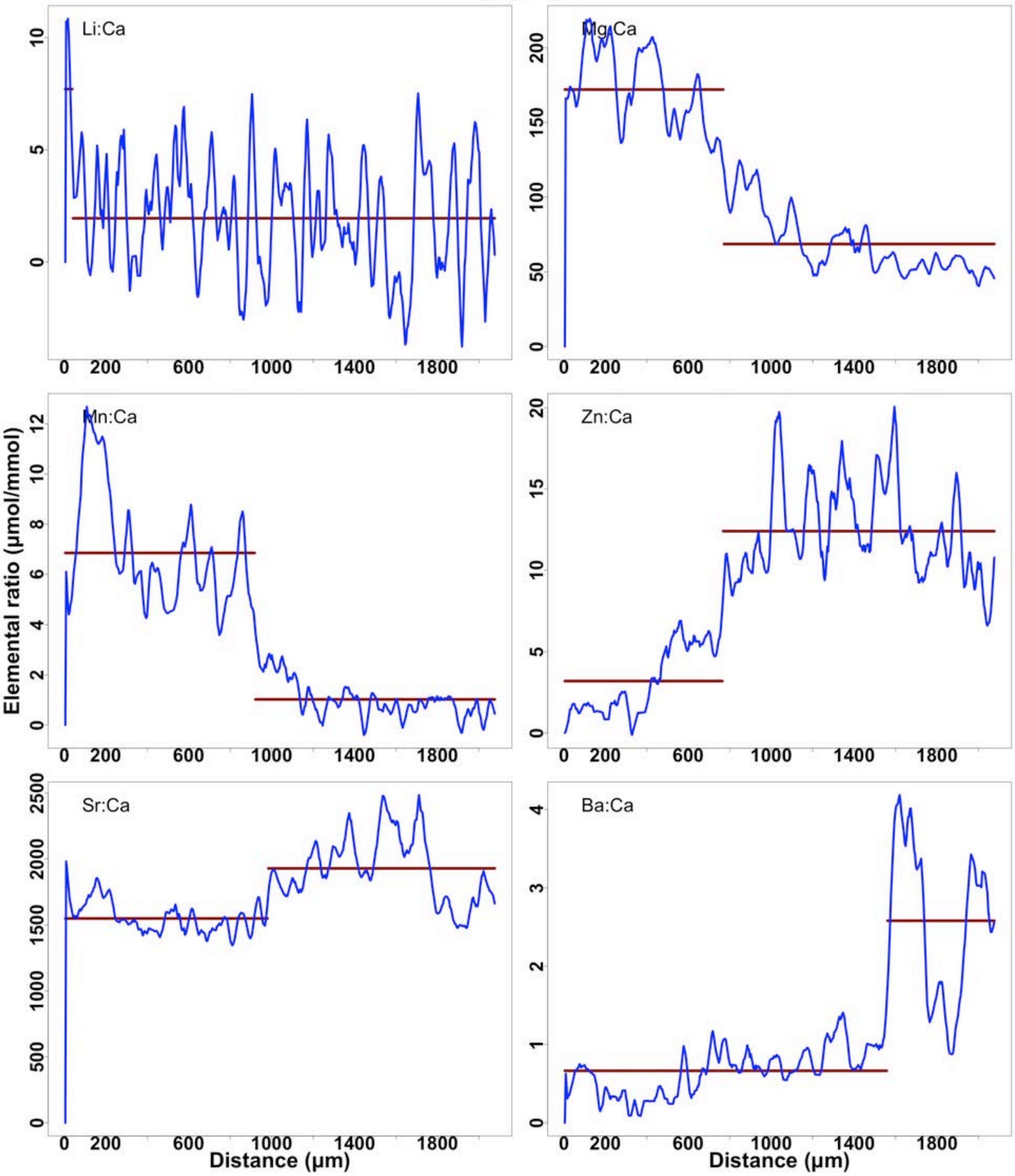

Li:Ca
Mg:Ca
Mn:Ca
Zn:Ca
Sr:Ca
Ba:Ca
Elemental ratio (µmol/mmol)
Distance (µm)
Distance (µm)

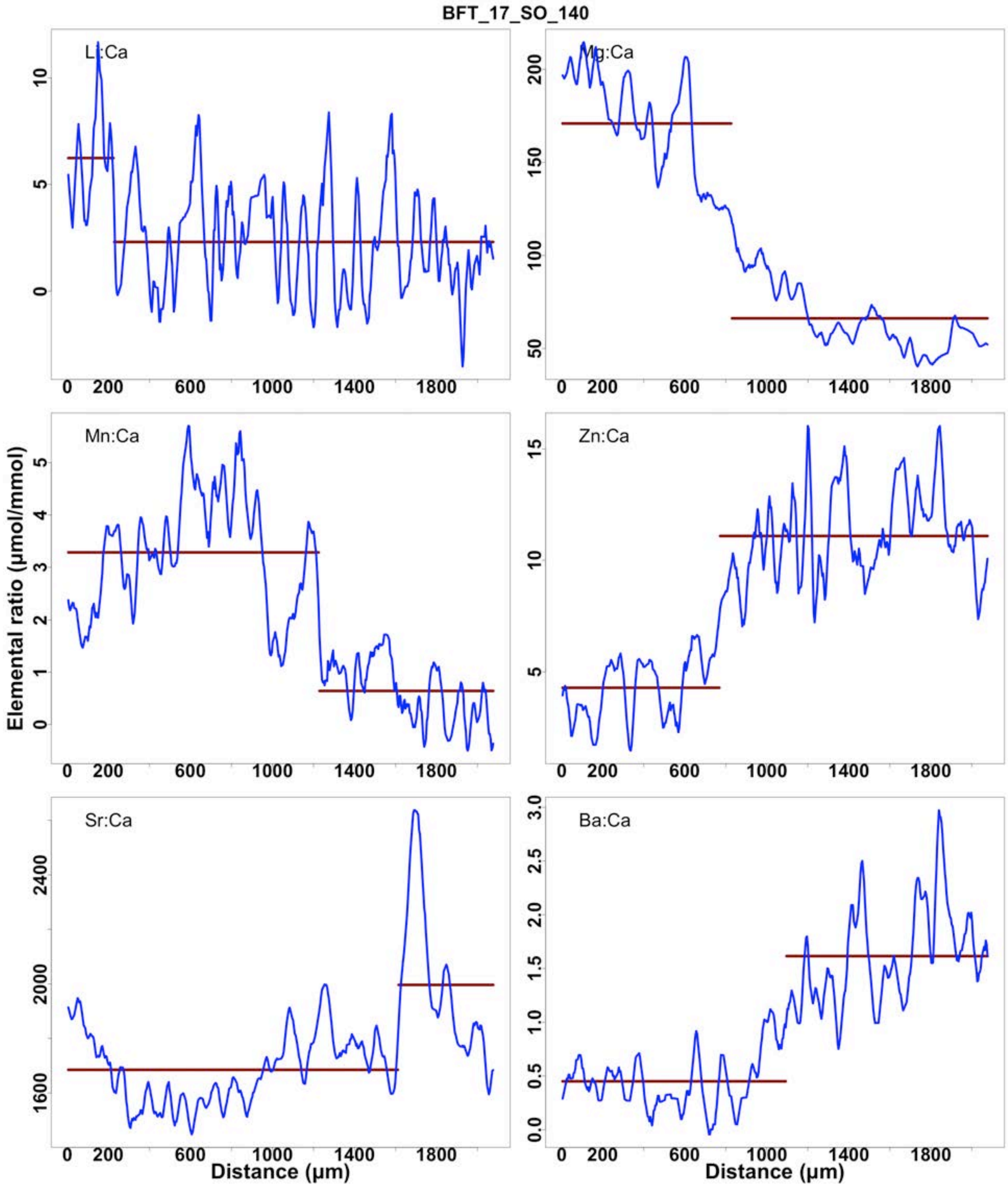

BFT_17_SO_140
Li:Ca
Mg:Ca
Mn:Ca
Zn:Ca
Sr:Ca
Ba:Ca
Elemental ratio (µmol/mmol)
Distance (µm)
Distance (µm)

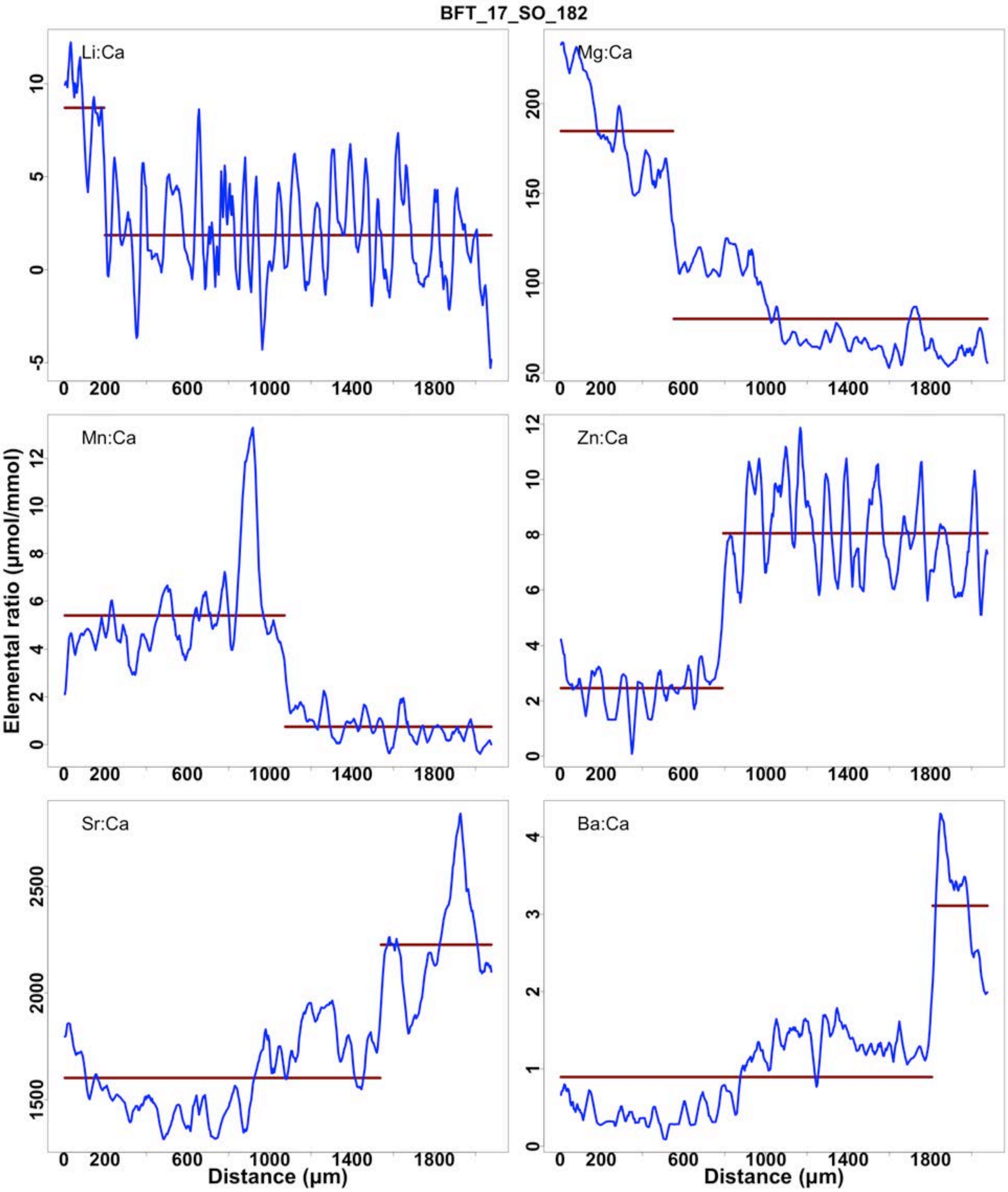
BFT_17_SO_182
Li:Ca
Mg:Ca
Mn:Ca
Zn:Ca
Sr:Ca
Ba:Ca
Elemental ratio (µmol/mmol)
Distance (µm)
Distance (µm)

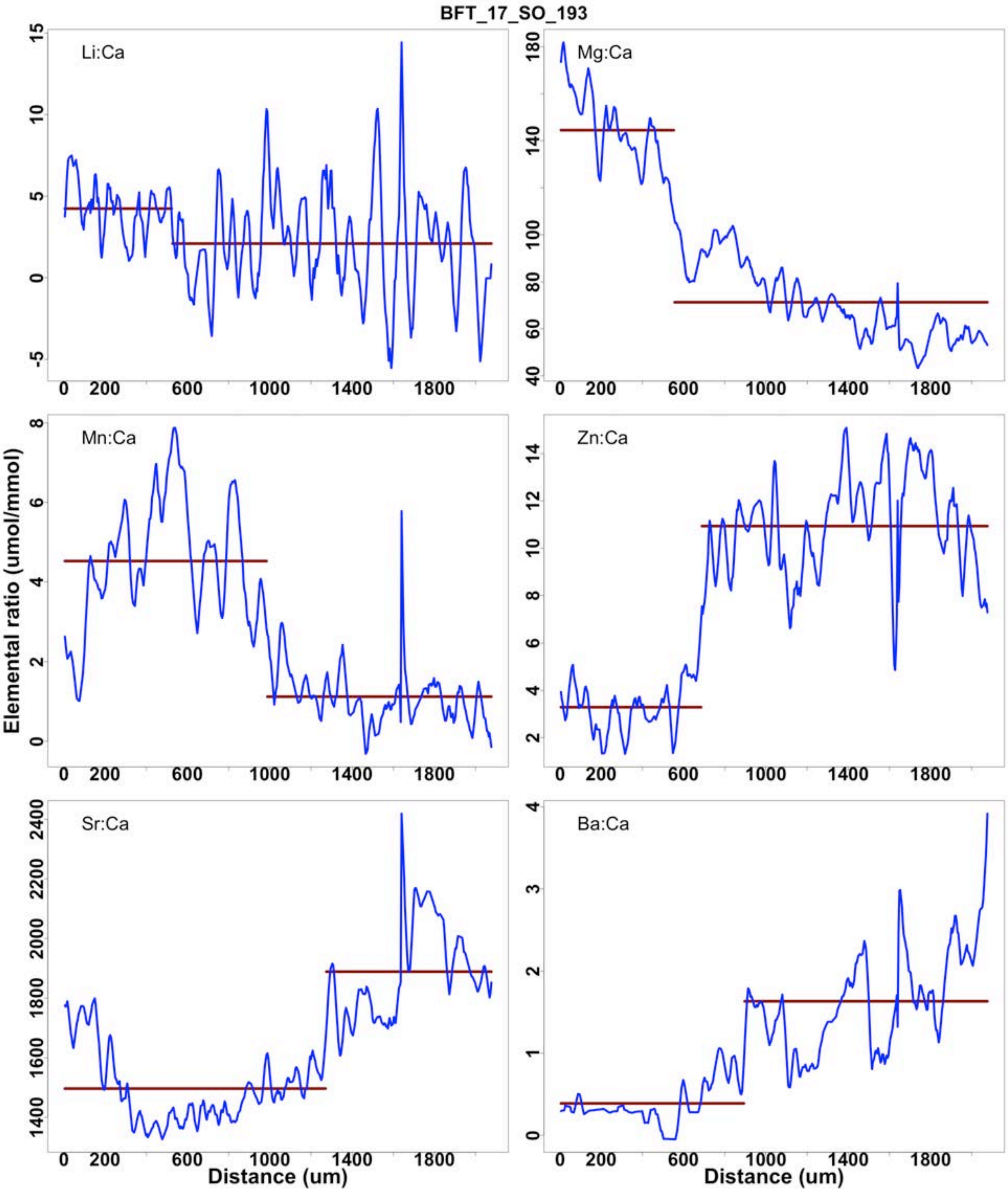
BFT_17_SO_193
Li:Ca
Mg:Ca
Mn:Ca
Zn:Ca
Sr:Ca
Ba:Ca
Elemental ratio (umol/mmol)
Distance (um)
Distance (um)

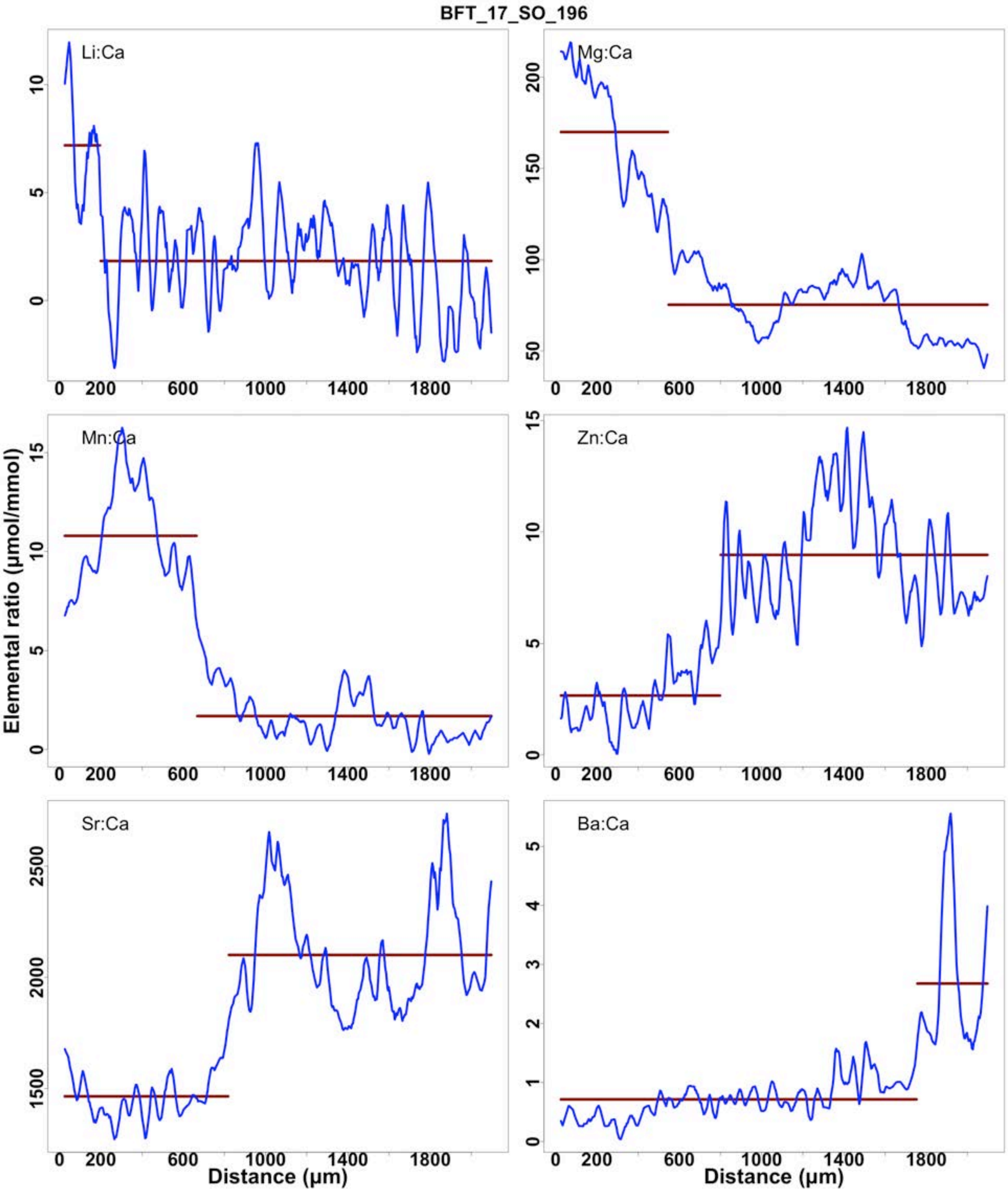
BFT_17_SO_196
Li:Ca
Mg:Ca
Mn:Ca
Zn:Ca
Sr:Ca
Ba:Ca
Elemental ratio (µmol/mmol)
Distance (µm)
Distance (µm)

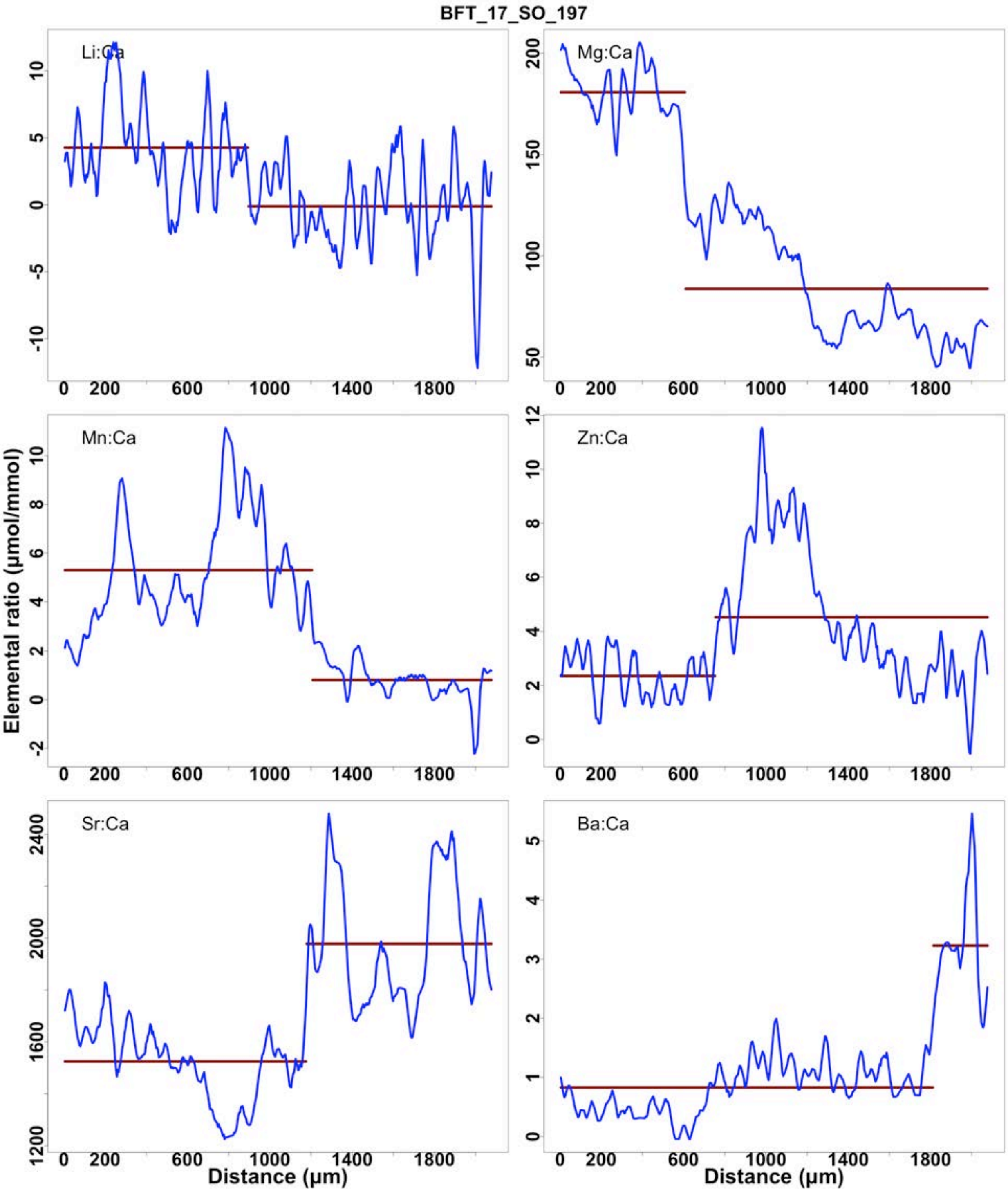
BFT_17_SO_197
Li:Ca
Mg:Ca
Mn:Ca
Zn:Ca
Sr:Ca
Ba:Ca
Elemental ratio (µmol/mmol)
Distance (µm)
Distance (µm)

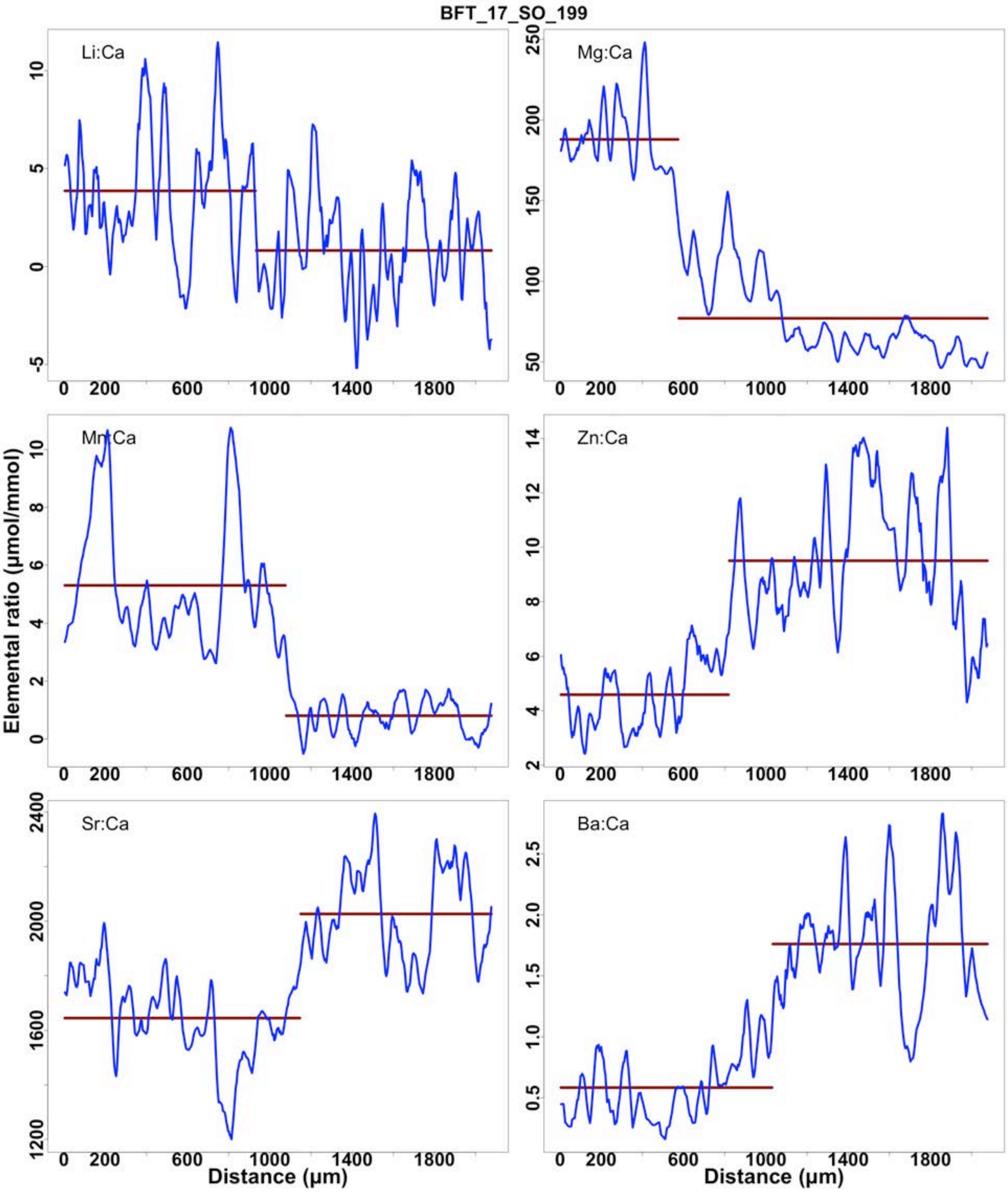
BFT_17_SO_199
Li:Ca
Mg:Ca
Mn:Ca
Zn:Ca
Sr:Ca
Ba:Ca
Elemental ratio (µmol/mmol)
Distance (µm)
0
200
600
1000
1400
1800

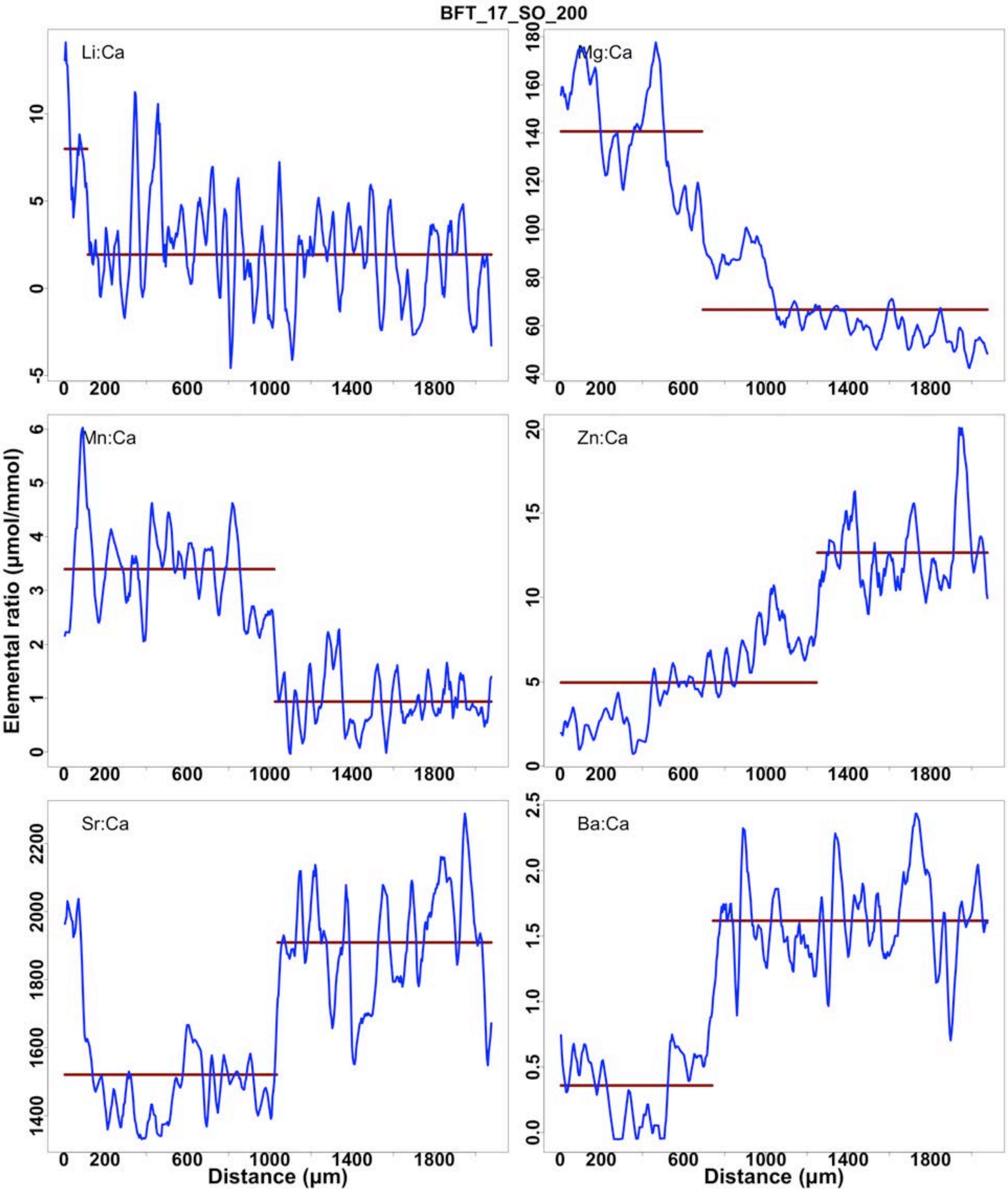

BFT_17_SO_200
Li:Ca
Mg:Ca
Mn:Ca
Zn:Ca
Sr:Ca
Ba:Ca
Elemental ratio (µmol/mmol)
Distance (µm)
Distance (µm)

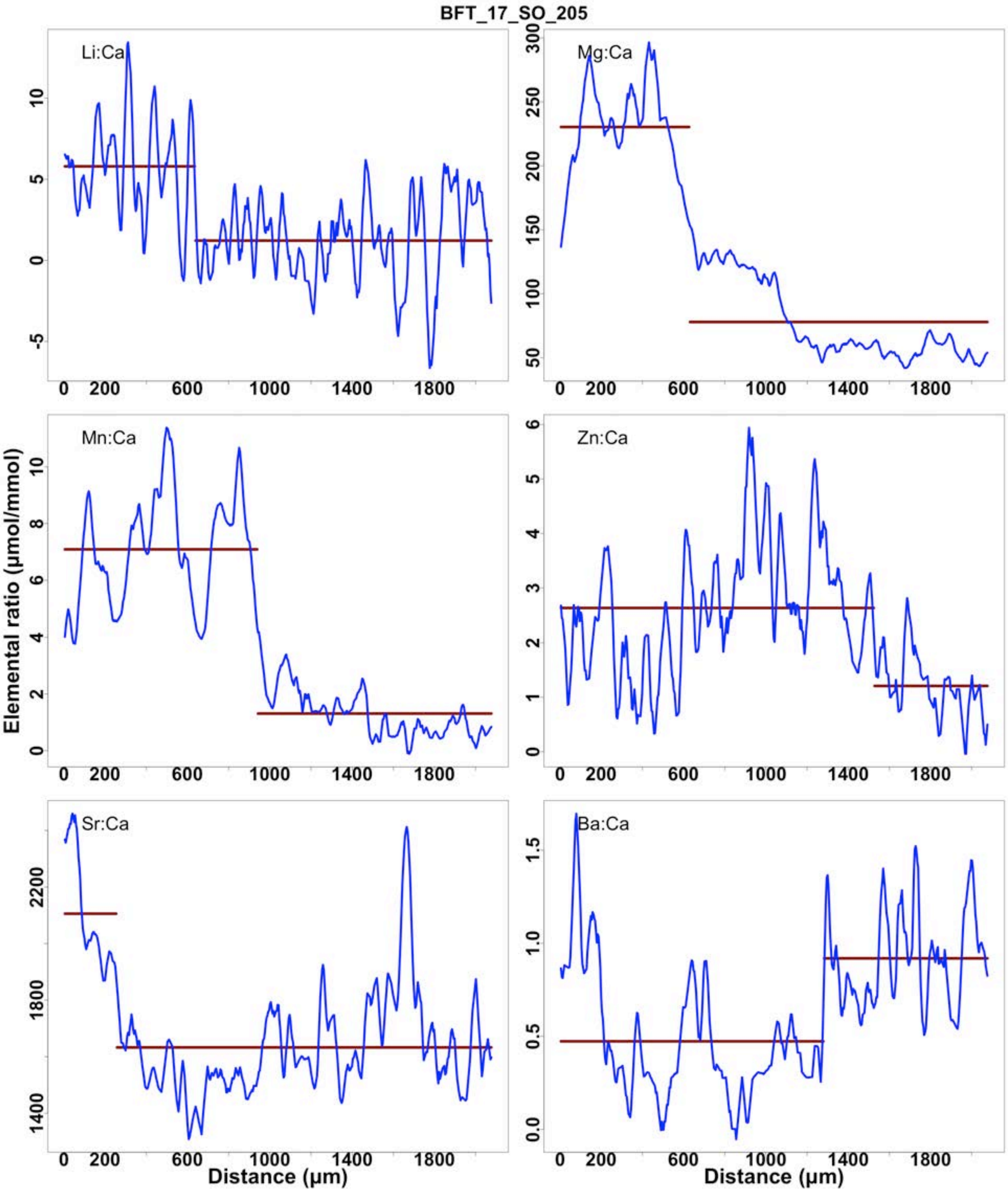

BFT_17_SO_205
Li:Ca
Mg:Ca
Mn:Ca
Zn:Ca
Sr:Ca
Ba:Ca
Elemental ratio (µmol/mmol)
Distance (µm)
Distance (µm)

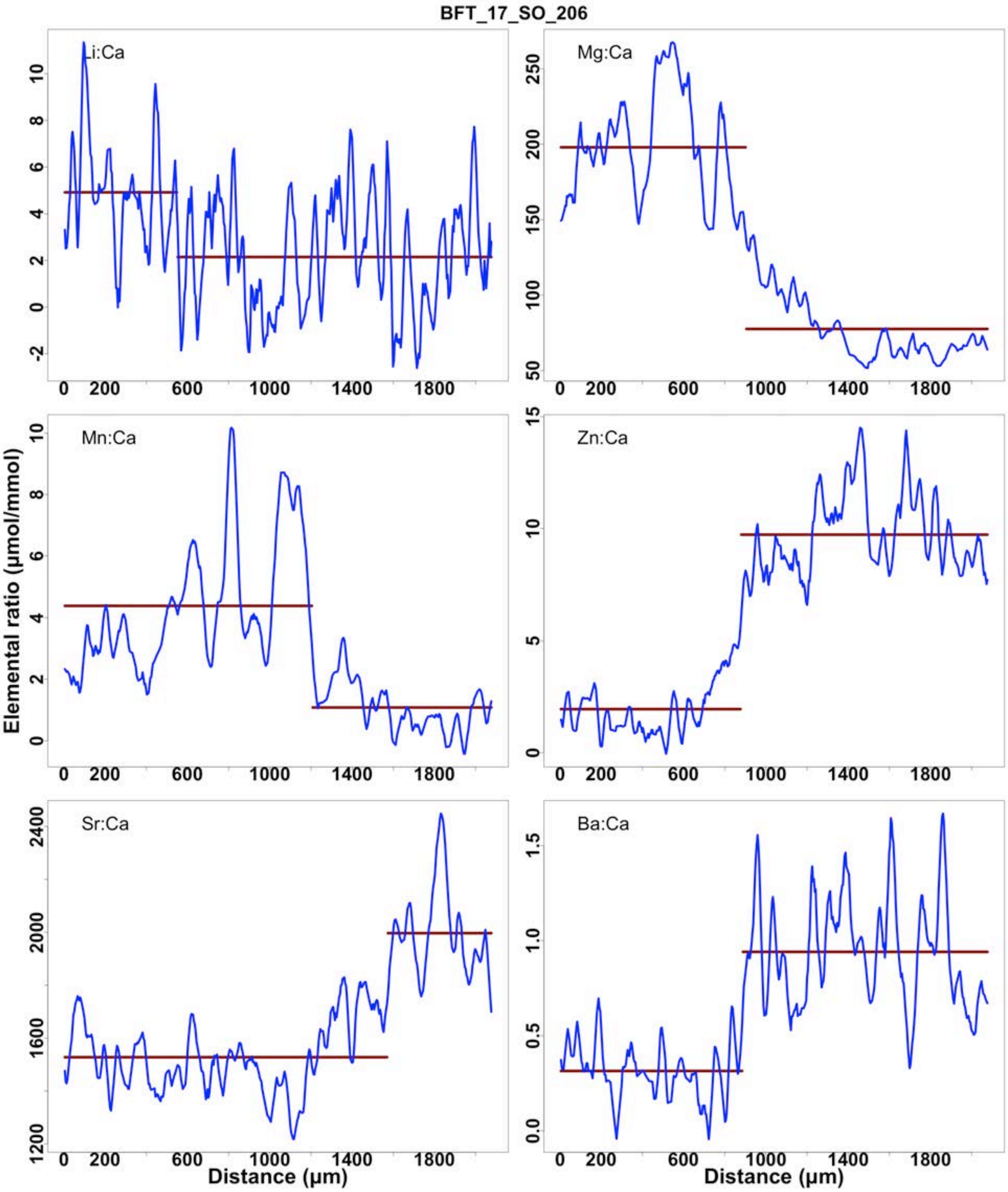
BFT_17_SO_206
Li:Ca
Mg:Ca
Mn:Ca
Zn:Ca
Sr:Ca
Ba:Ca
Elemental ratio (µmol/mmol)
Distance (µm)
Distance (µm)

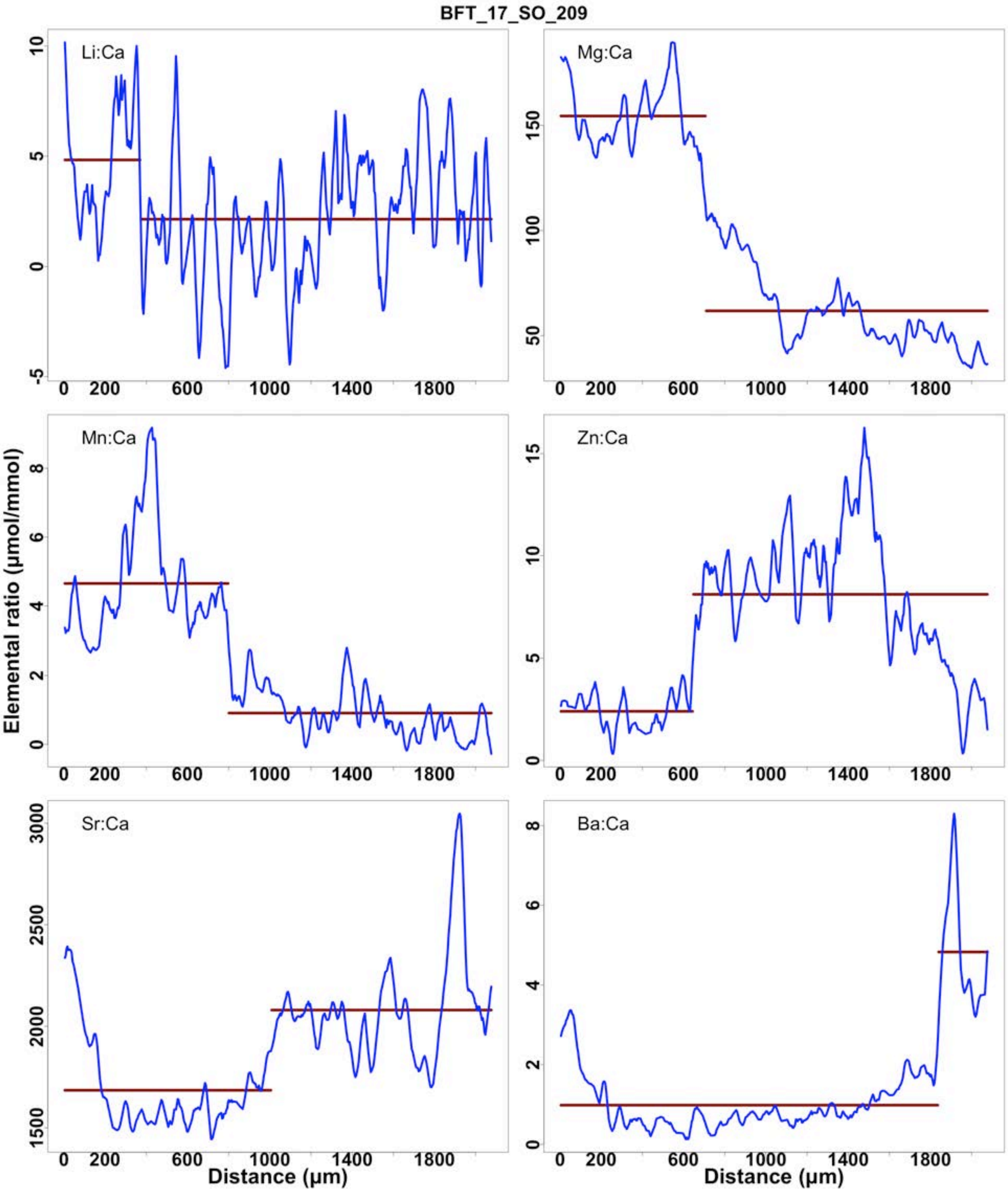
BFT_17_SO_209
Li:Ca
Mg:Ca
Mn:Ca
Zn:Ca
Sr:Ca
Ba:Ca
Elemental ratio (µmol/mmol)
Distance (µm)
Distance (µm)

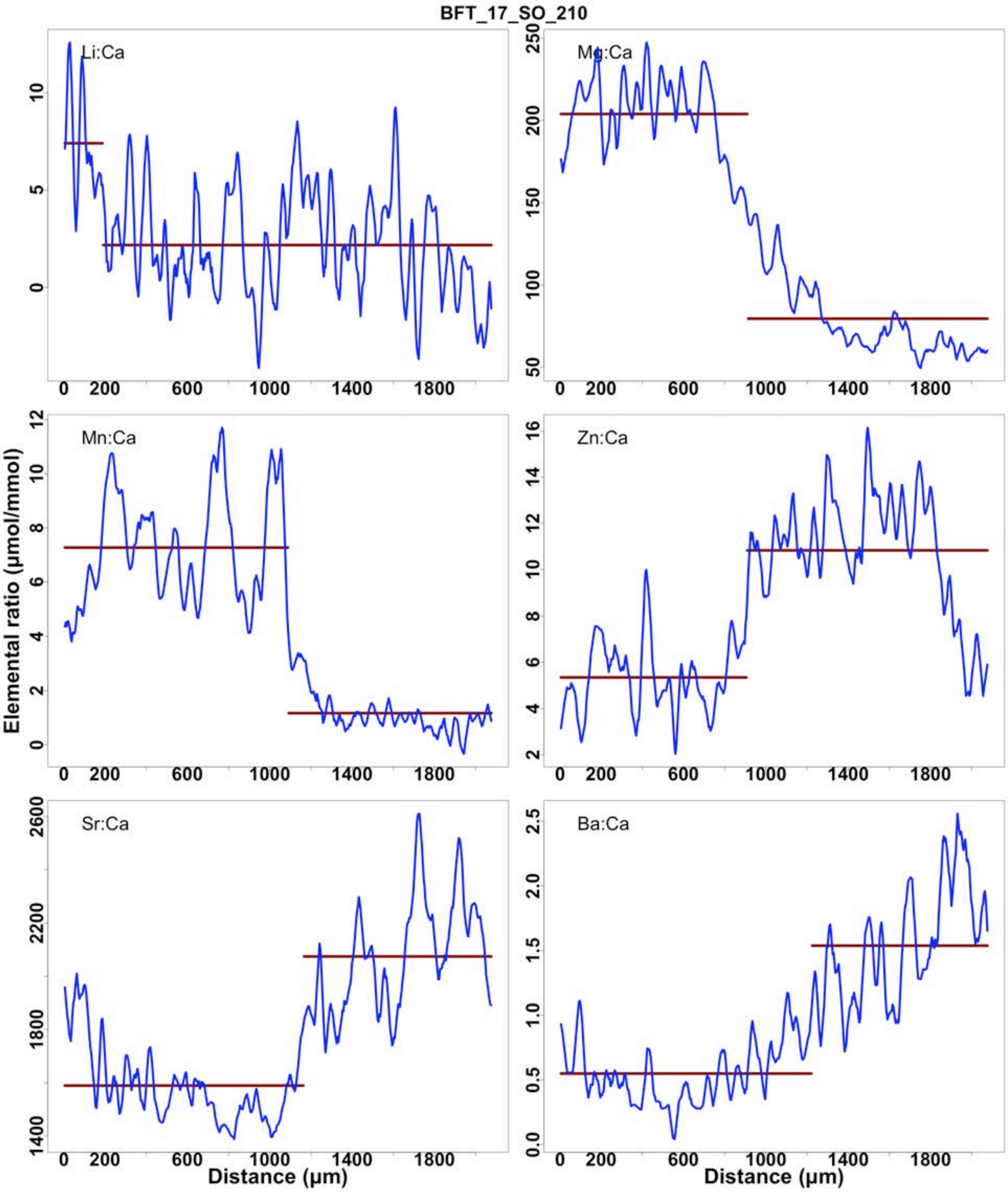
BFT_17_SO_210
Li:Ca
Mg:Ca
Mn:Ca
Zn:Ca
Sr:Ca
Ba:Ca
Elemental ratio (µmol/mmol)
Distance (µm)
Distance (µm)

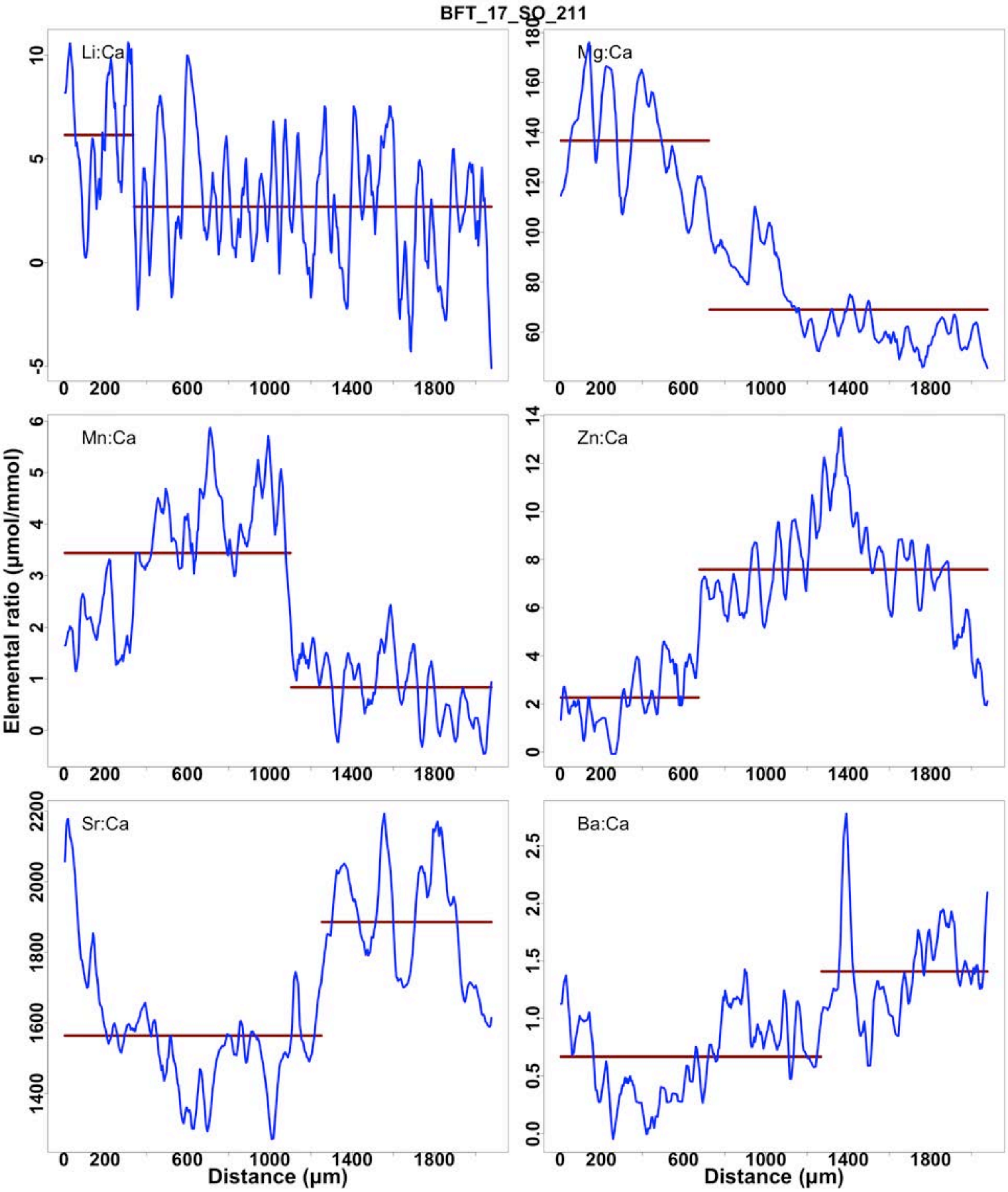

BFT_17_SO_211
Li:Ca
Mg:Ca
Mn:Ca
Zn:Ca
Sr:Ca
Ba:Ca
Elemental ratio (µmol/mmol)
Distance (µm)
Distance (µm)

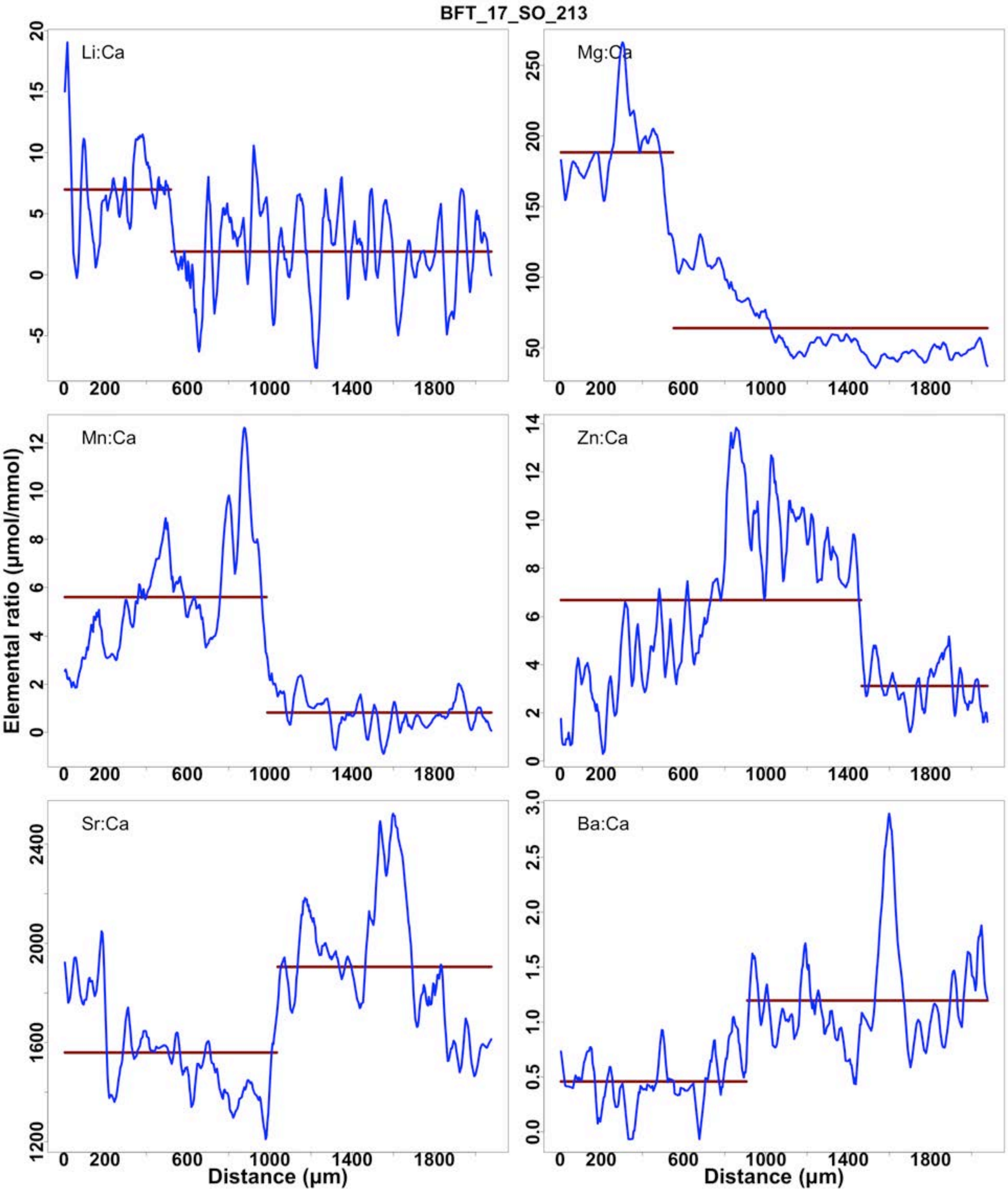

BFT_17_SO_213
Li:Ca
Mg:Ca
Mn:Ca
Zn:Ca
Sr:Ca
Ba:Ca
Elemental ratio (µmol/mmol)
Distance (µm)
Distance (µm)

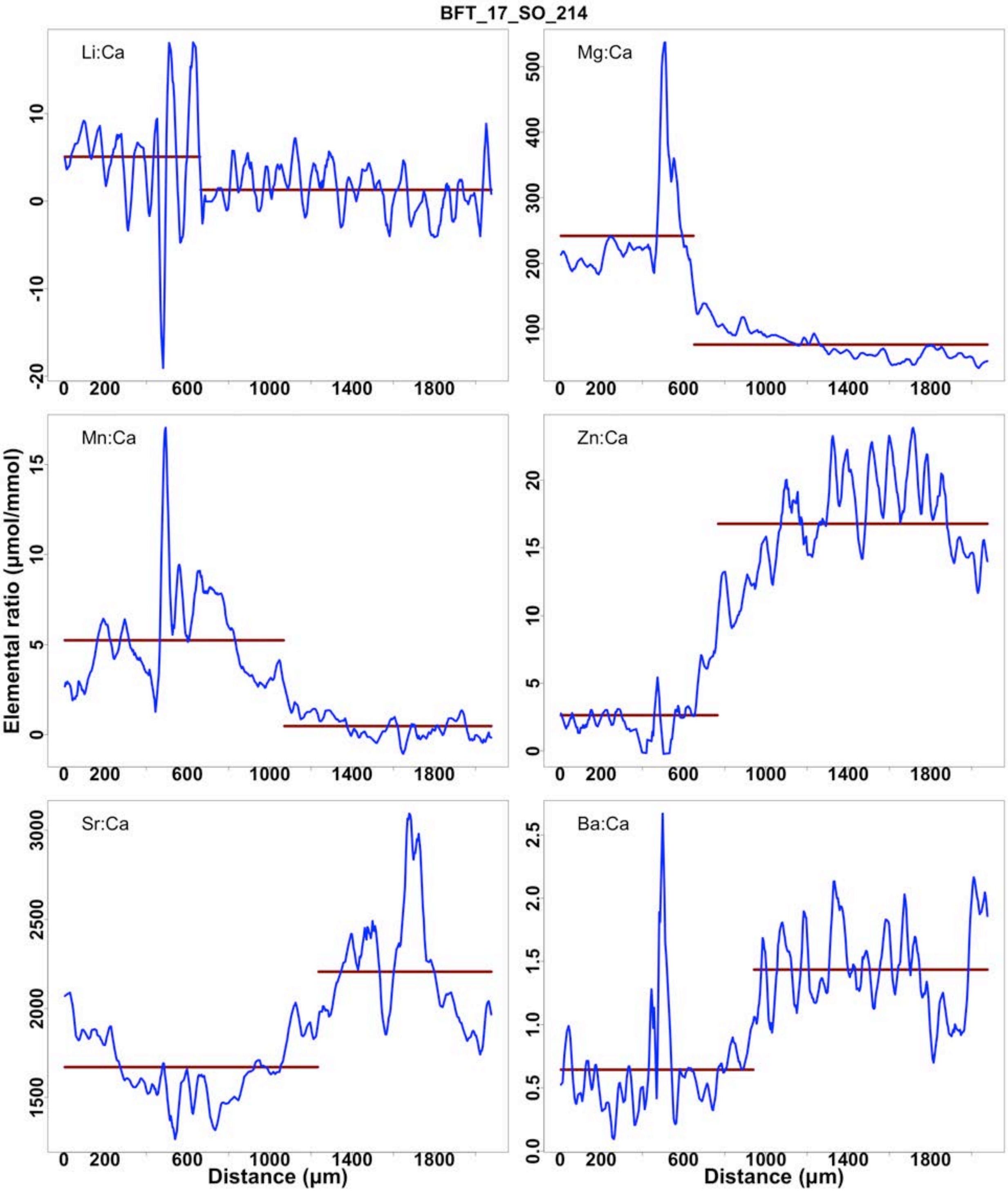
BFT_17_SO_214
Li:Ca
Mg:Ca
Mn:Ca
Zn:Ca
Sr:Ca
Ba:Ca
Elemental ratio (µmol/mmol)
Distance (µm)
Distance (µm)

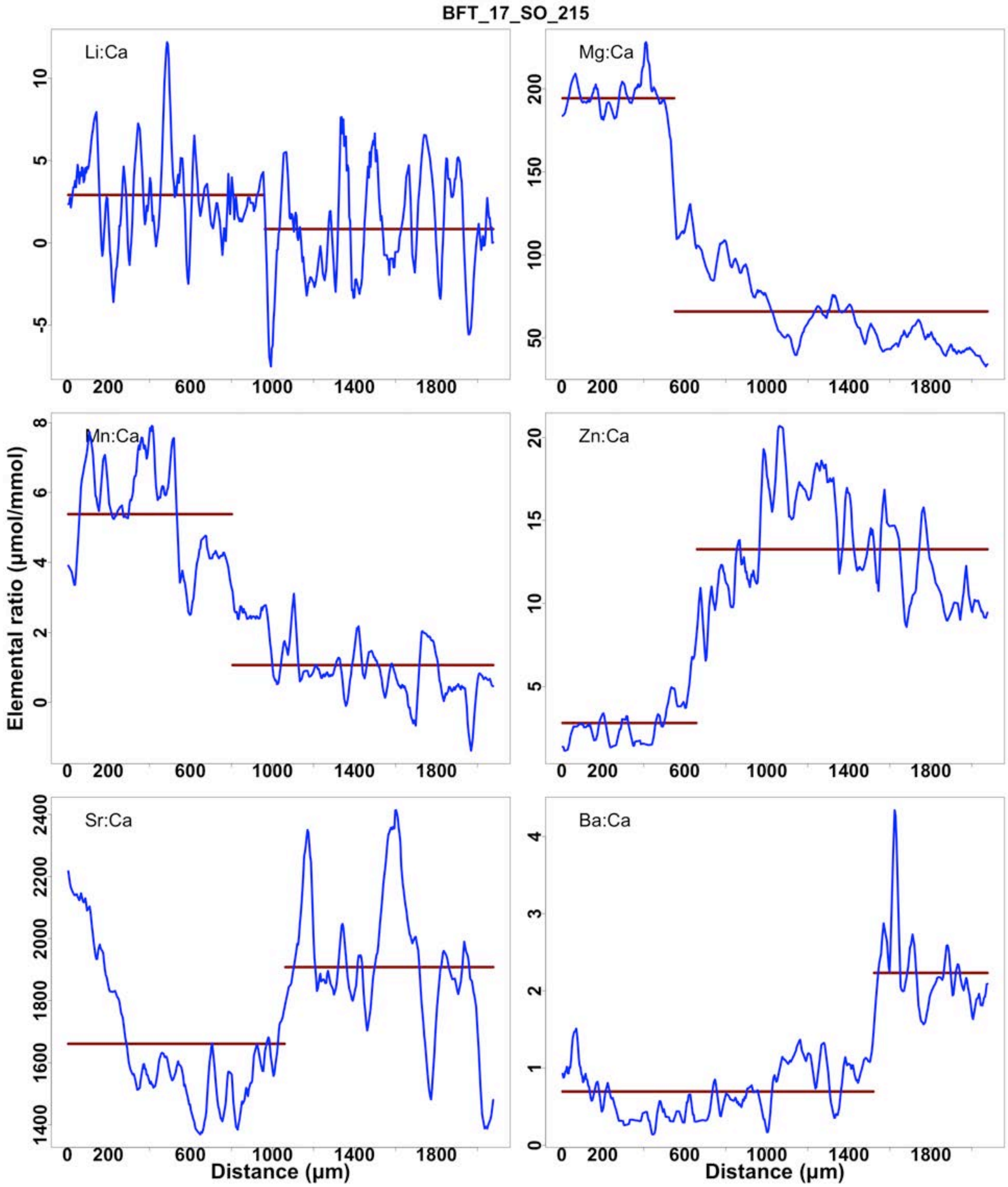
BFT_17_SO_215
Li:Ca
Mg:Ca
Mn:Ca
Zn:Ca
Sr:Ca
Ba:Ca
Elemental ratio (µmol/mmol)
Distance (µm)
Distance (µm)

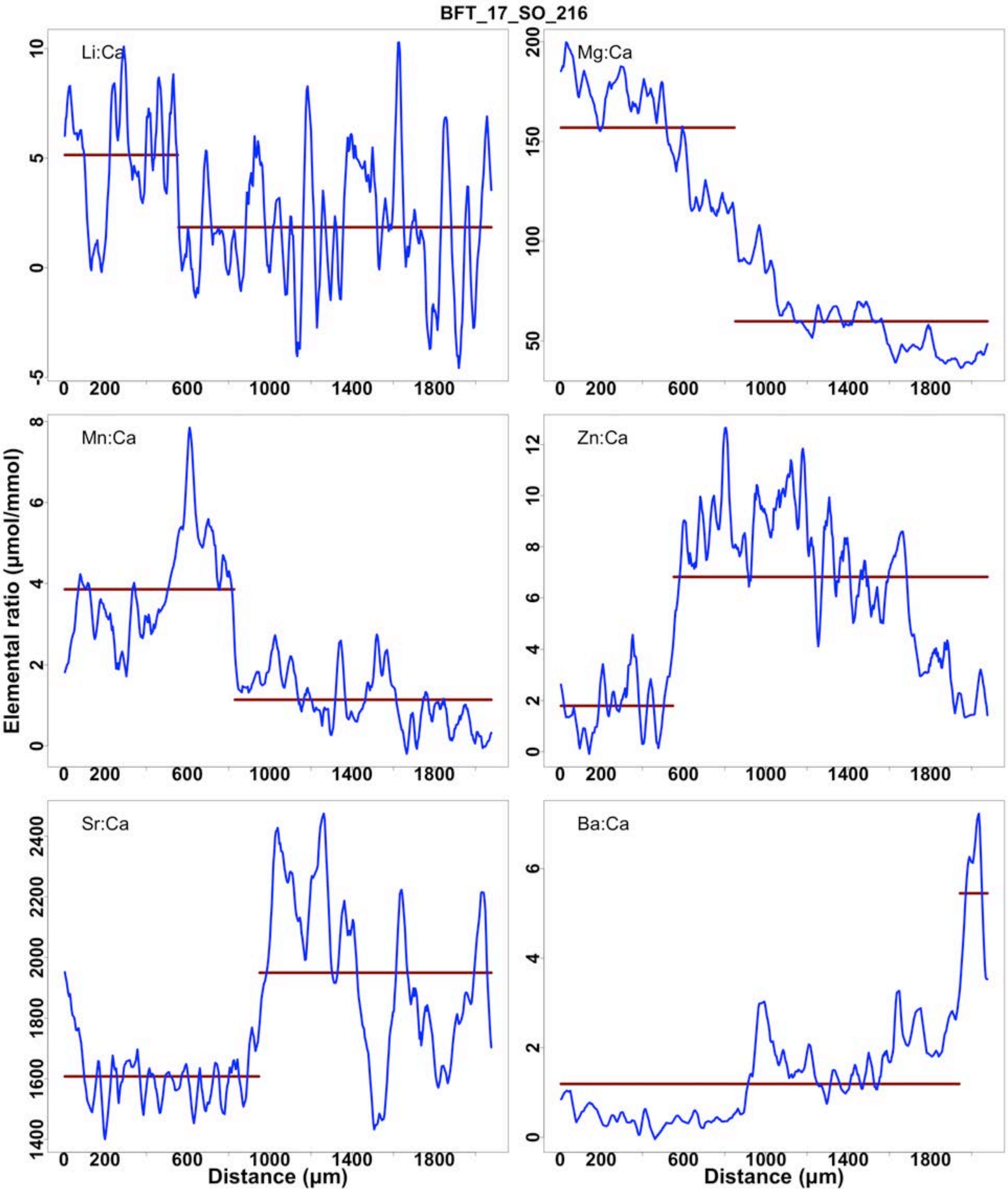

BFT_17_SO_216
Li:Ca
Mg:Ca
Mn:Ca
Zn:Ca
Sr:Ca
Ba:Ca
Elemental ratio (µmol/mmol)
Distance (µm)
Distance (µm)

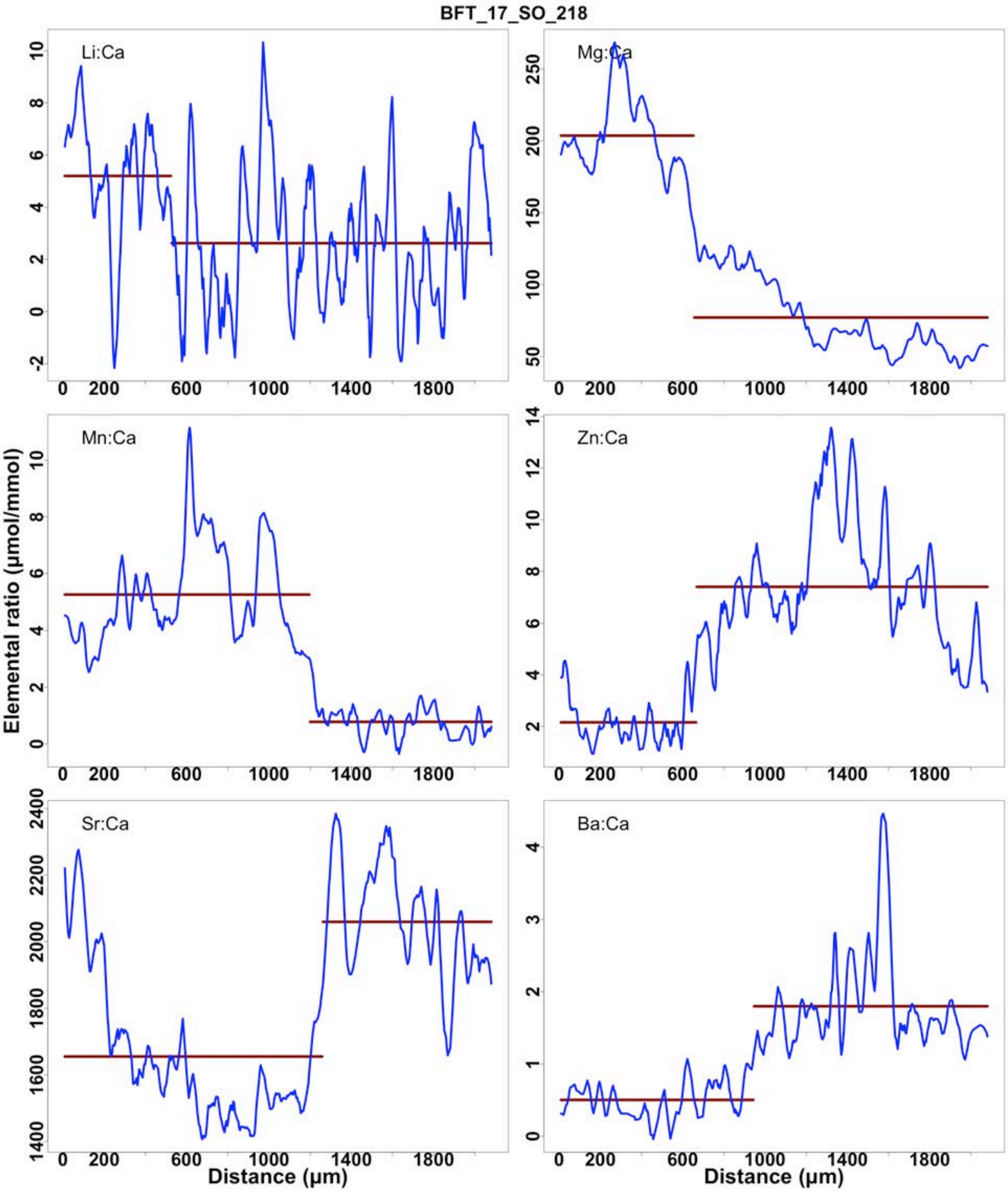
BFT_17_SO_218
Li:Ca
Mg:Ca
Mn:Ca
Zn:Ca
Sr:Ca
Ba:Ca
Elemental ratio (µmol/mmol)
Distance (µm)
Distance (µm)

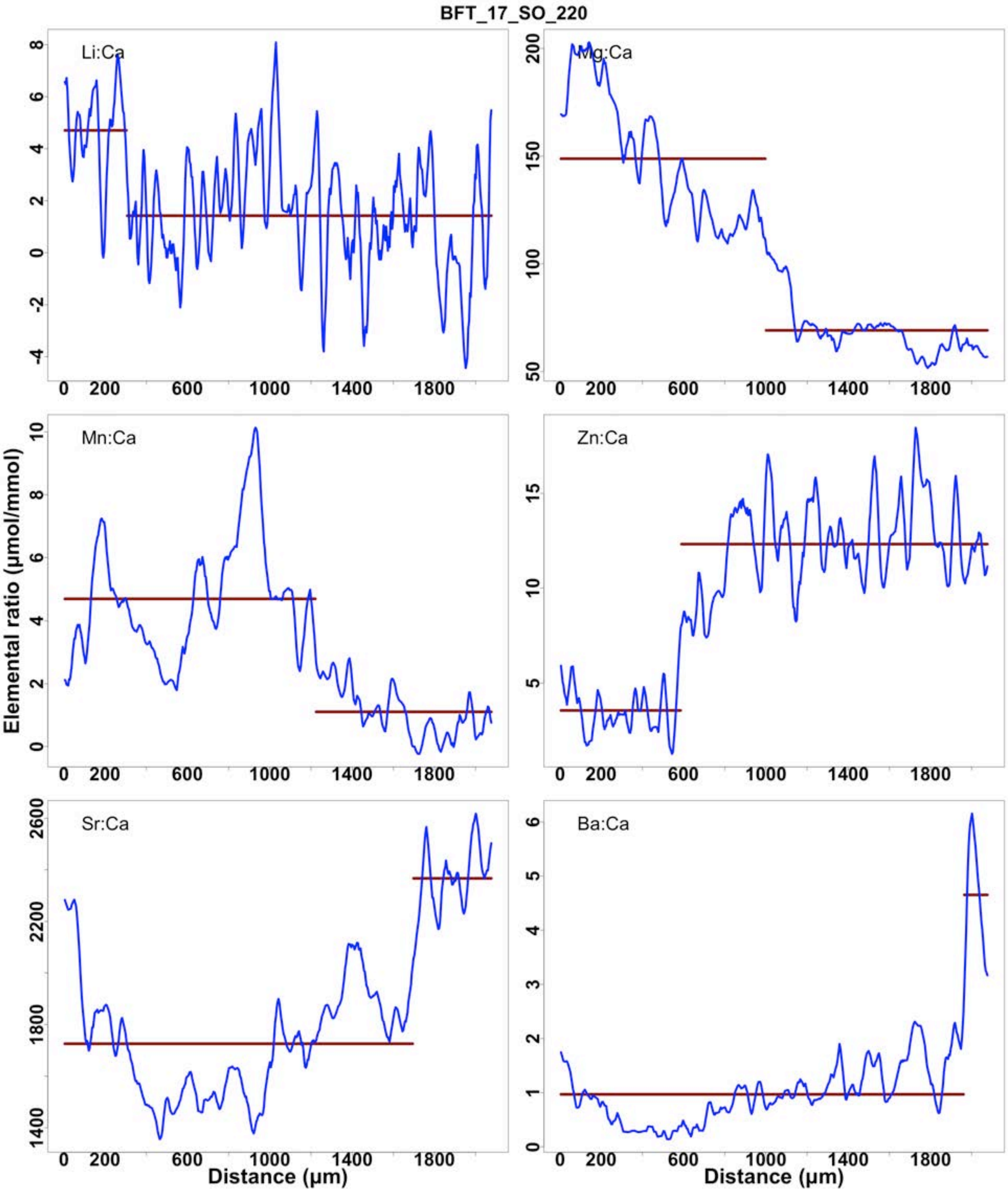

BFT_17_SO_220
Li:Ca
Mg:Ca
Mn:Ca
Zn:Ca
Sr:Ca
Ba:Ca
Elemental ratio (µmol/mmol)
Distance (µm)
Distance (µm)

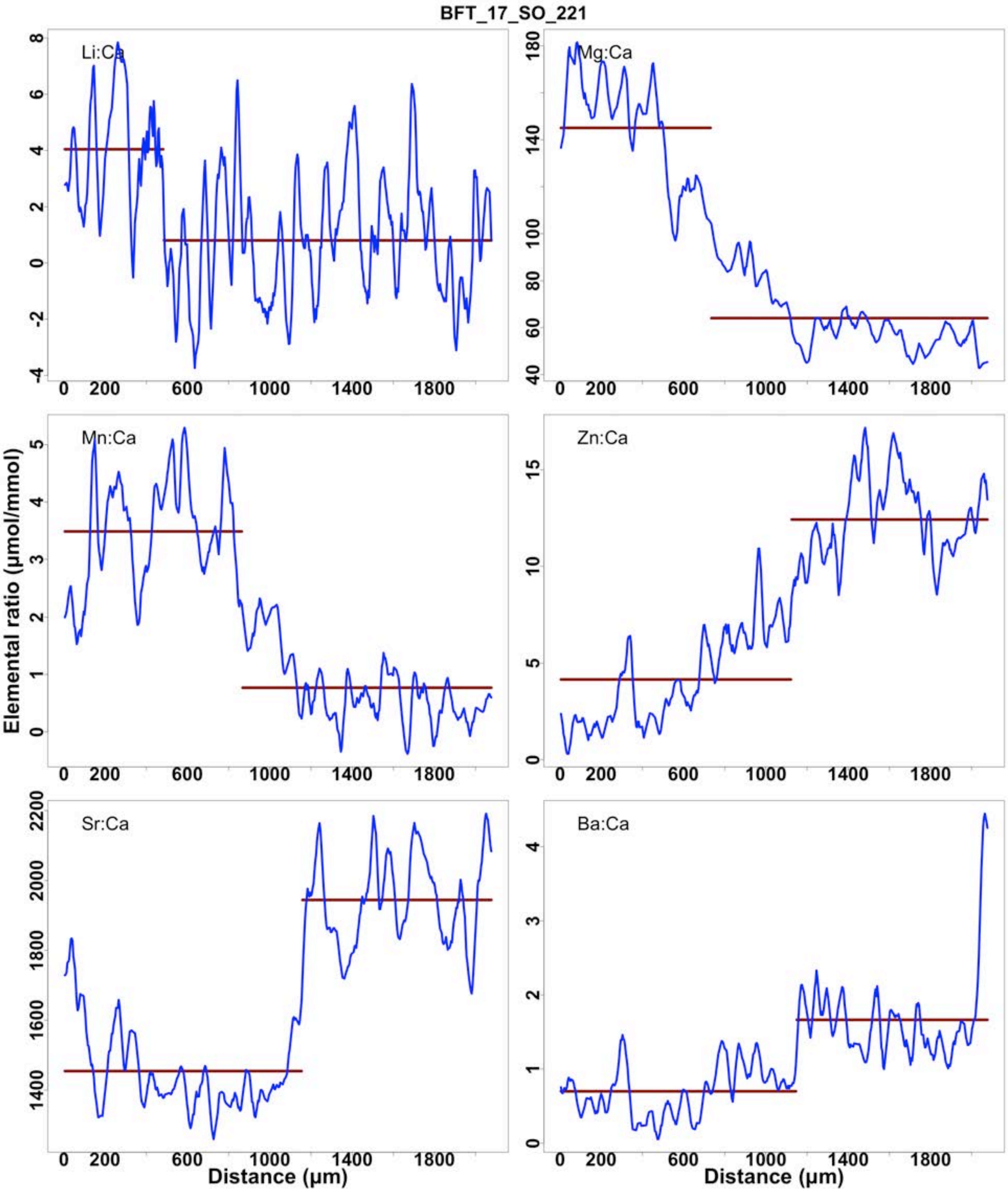
BFT_17_SO_221
Li:Ca
Mg:Ca
Mn:Ca
Zn:Ca
Sr:Ca
Ba:Ca
Elemental ratio (µmol/mmol)
Distance (µm)
Distance (µm)

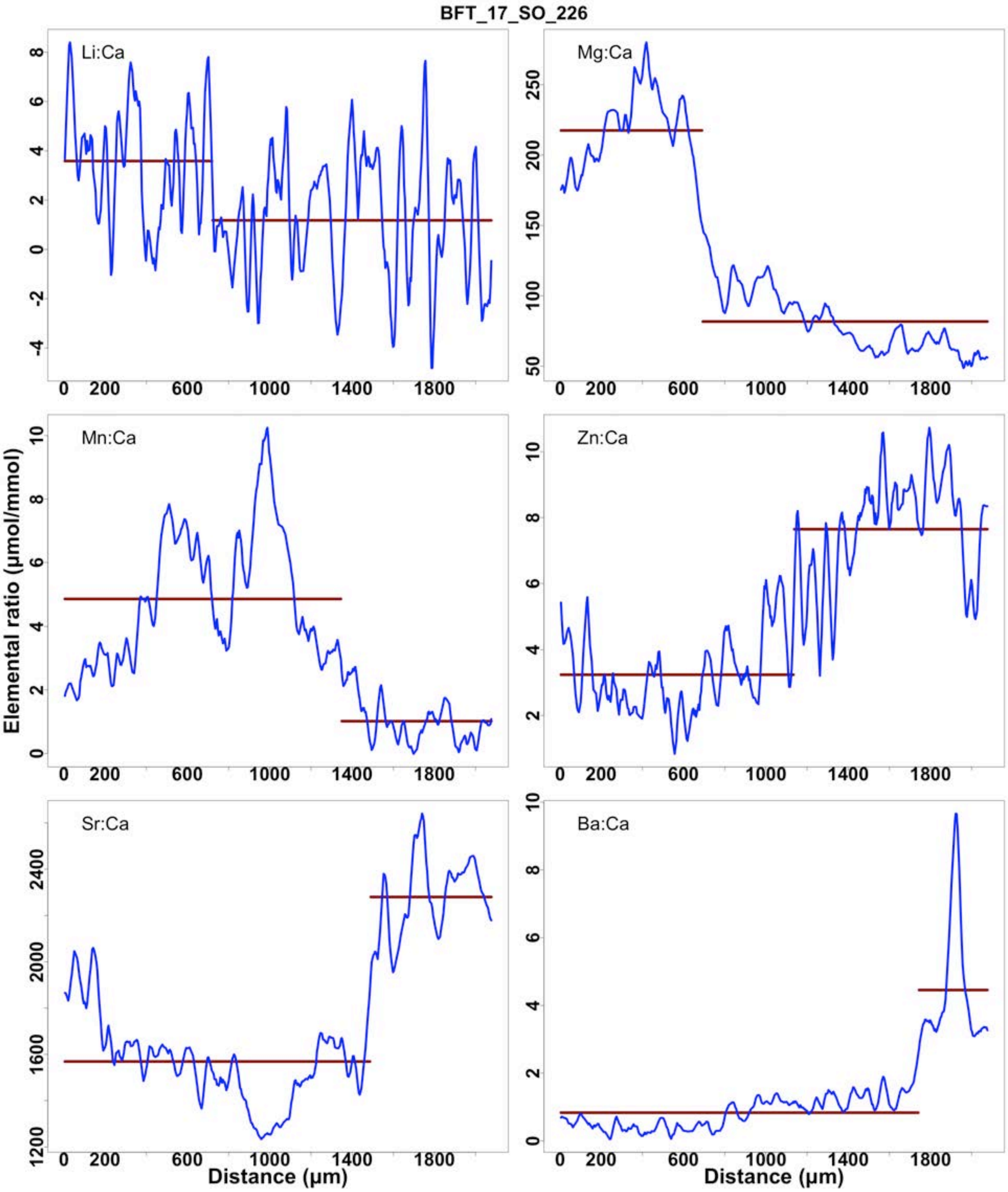

BFT_17_SO_226
Li:Ca
Mg:Ca
Mn:Ca
Zn:Ca
Sr:Ca
Ba:Ca
Elemental ratio (µmol/mmol)
Distance (µm)
Distance (µm)

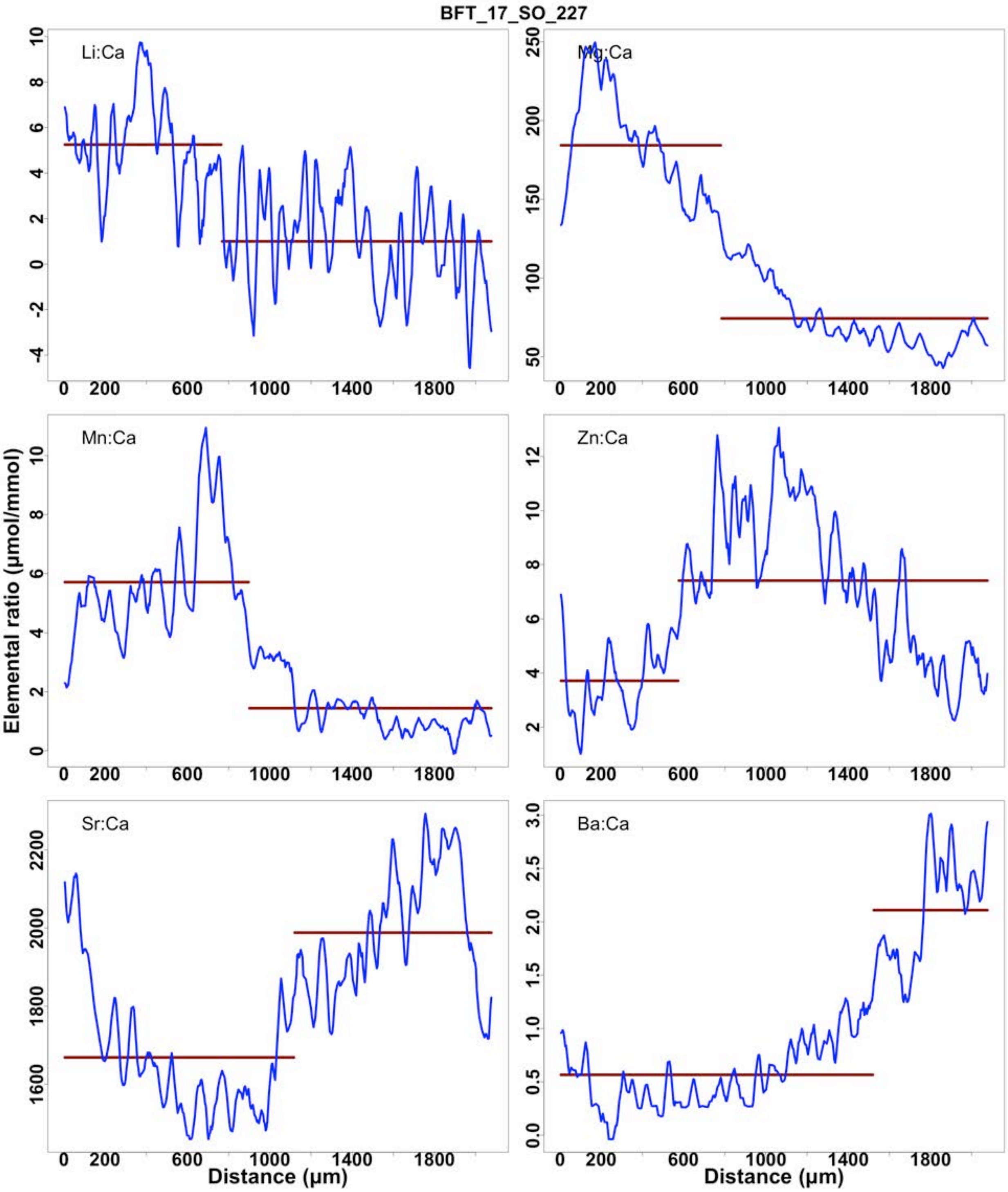
BFT_17_SO_227
Li:Ca
Mg:Ca
Mn:Ca
Zn:Ca
Sr:Ca
Ba:Ca
Elemental ratio (µmol/mmol)
Distance (µm)
Distance (µm)

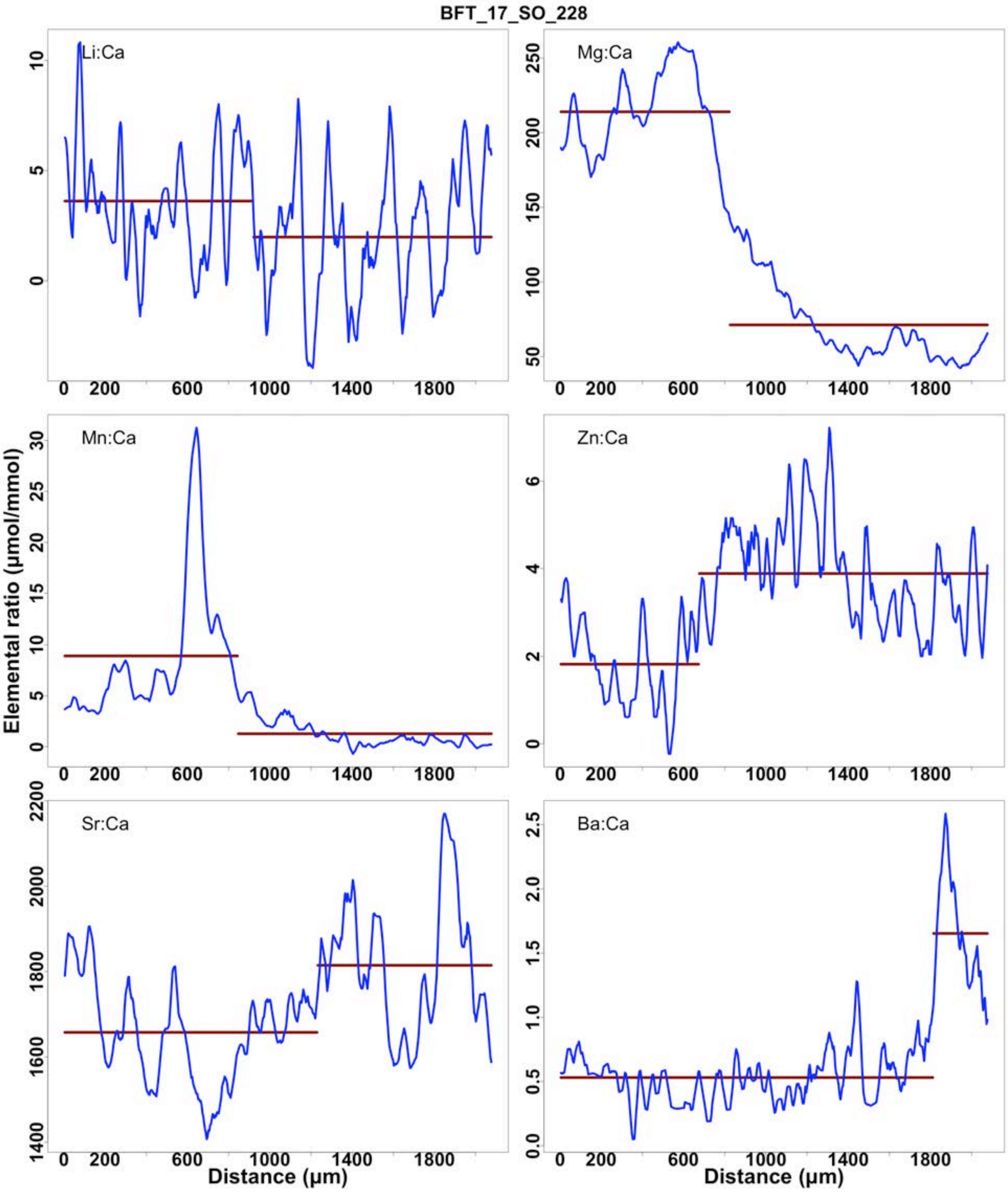
BFT_17_SO_228
Li:Ca
Mg:Ca
Mn:Ca
Zn:Ca
Sr:Ca
Ba:Ca
Elemental ratio (µmol/mmol)
Distance (µm)
Distance (µm)
0 200 600 1000 1400 1800

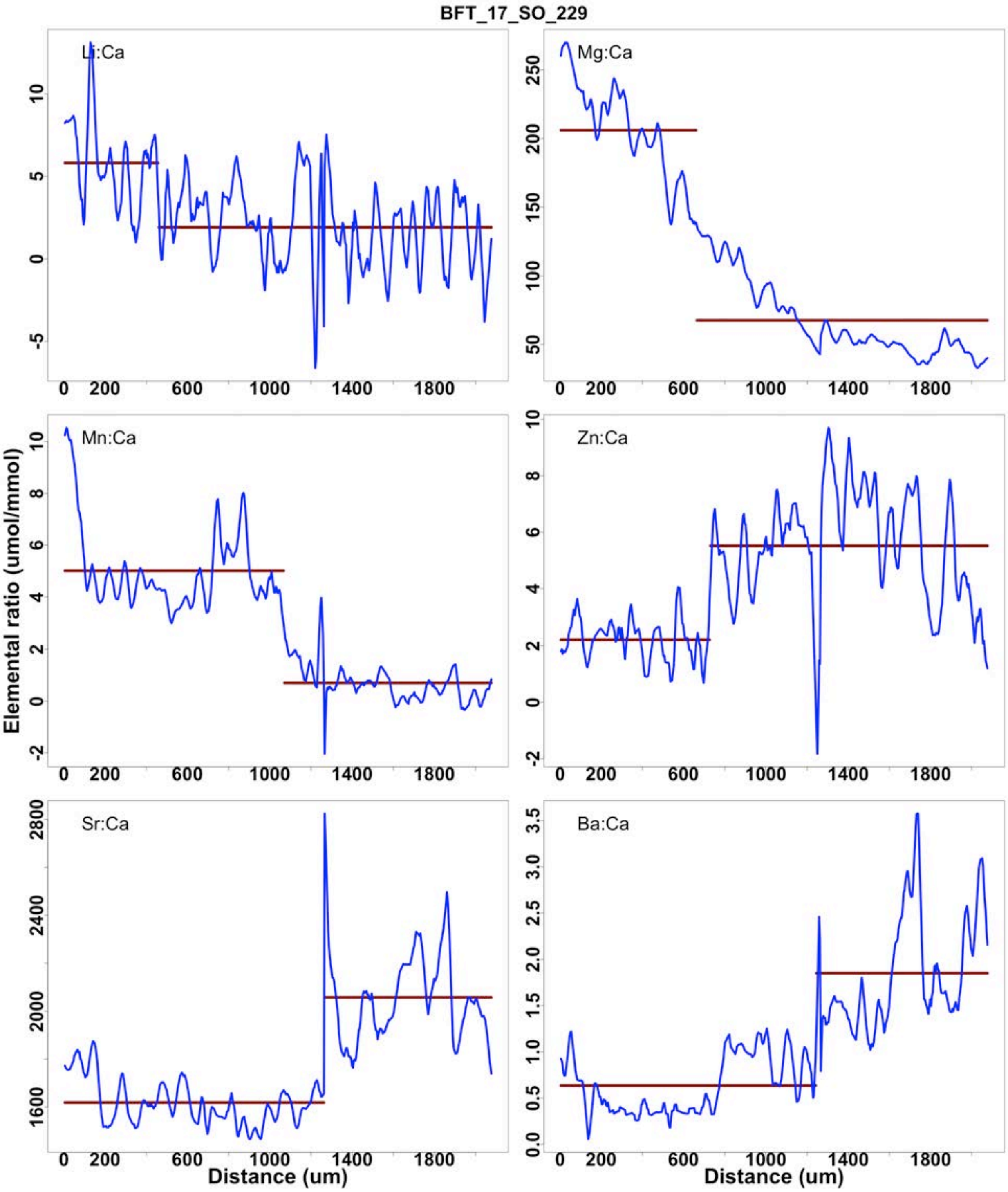
BFT_17_SO_229
Li:Ca
Mg:Ca
Mn:Ca
Zn:Ca
Sr:Ca
Ba:Ca
Elemental ratio (umol/mmol)
Distance (um)
Distance (um)

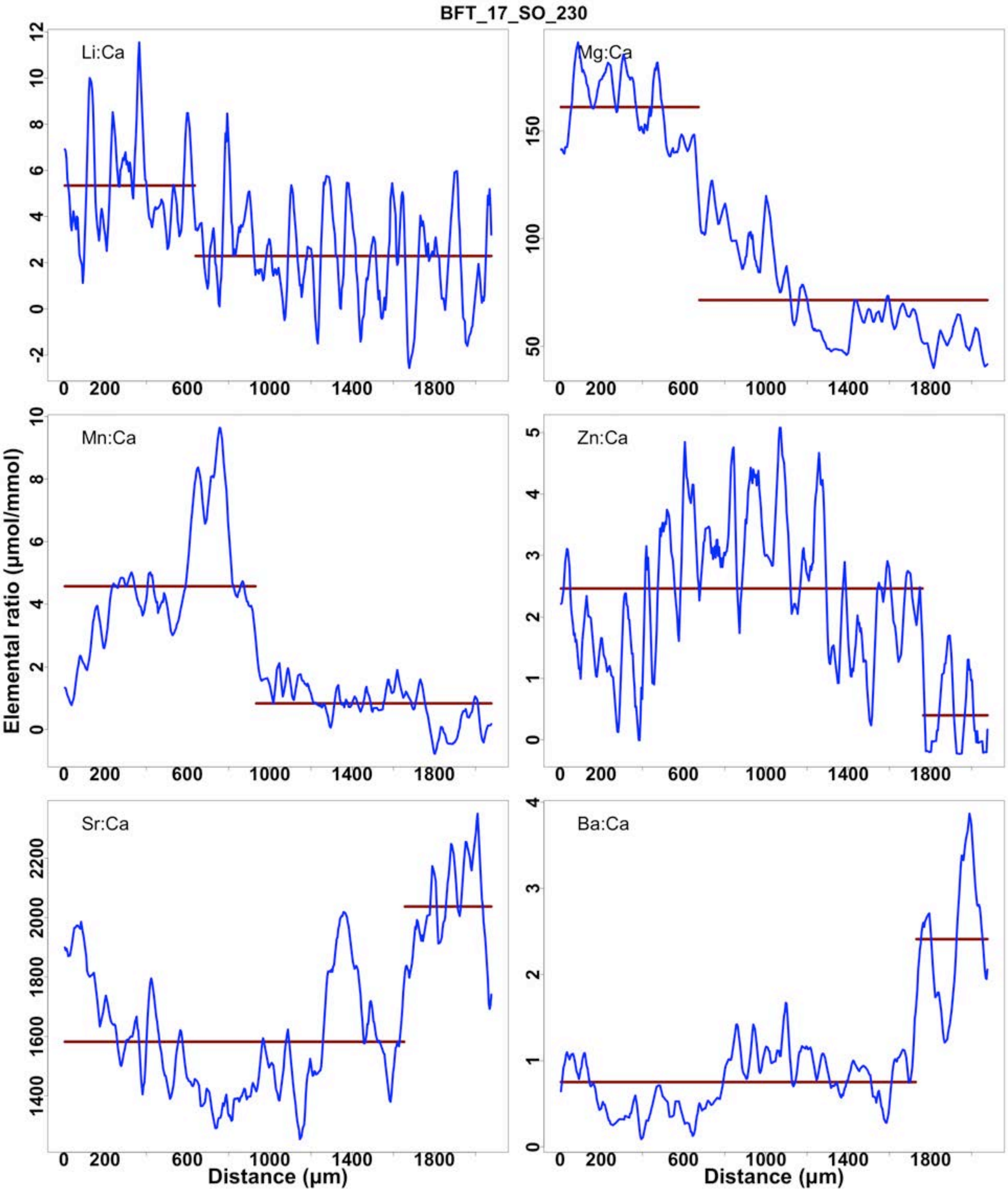

BFT_17_SO_230
Li:Ca
Mg:Ca
Mn:Ca
Zn:Ca
Sr:Ca
Ba:Ca
Elemental ratio (µmol/mmol)
Distance (µm)
Distance (µm)

BFT_17_SO_233

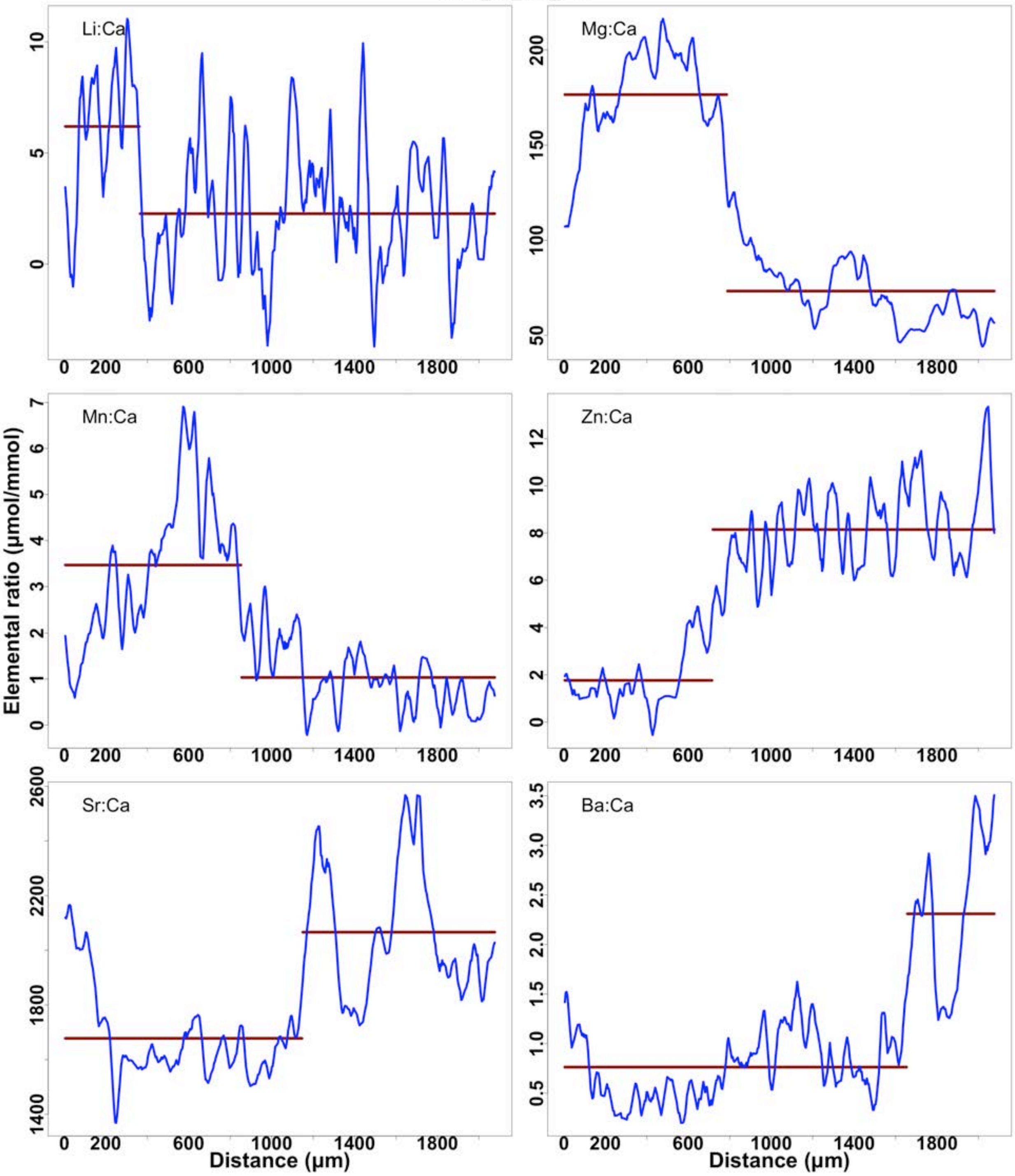

Li:Ca
Mg:Ca
Mn:Ca
Zn:Ca
Sr:Ca
Ba:Ca
Elemental ratio (µmol/mmol)
Distance (µm)
Distance (µm)

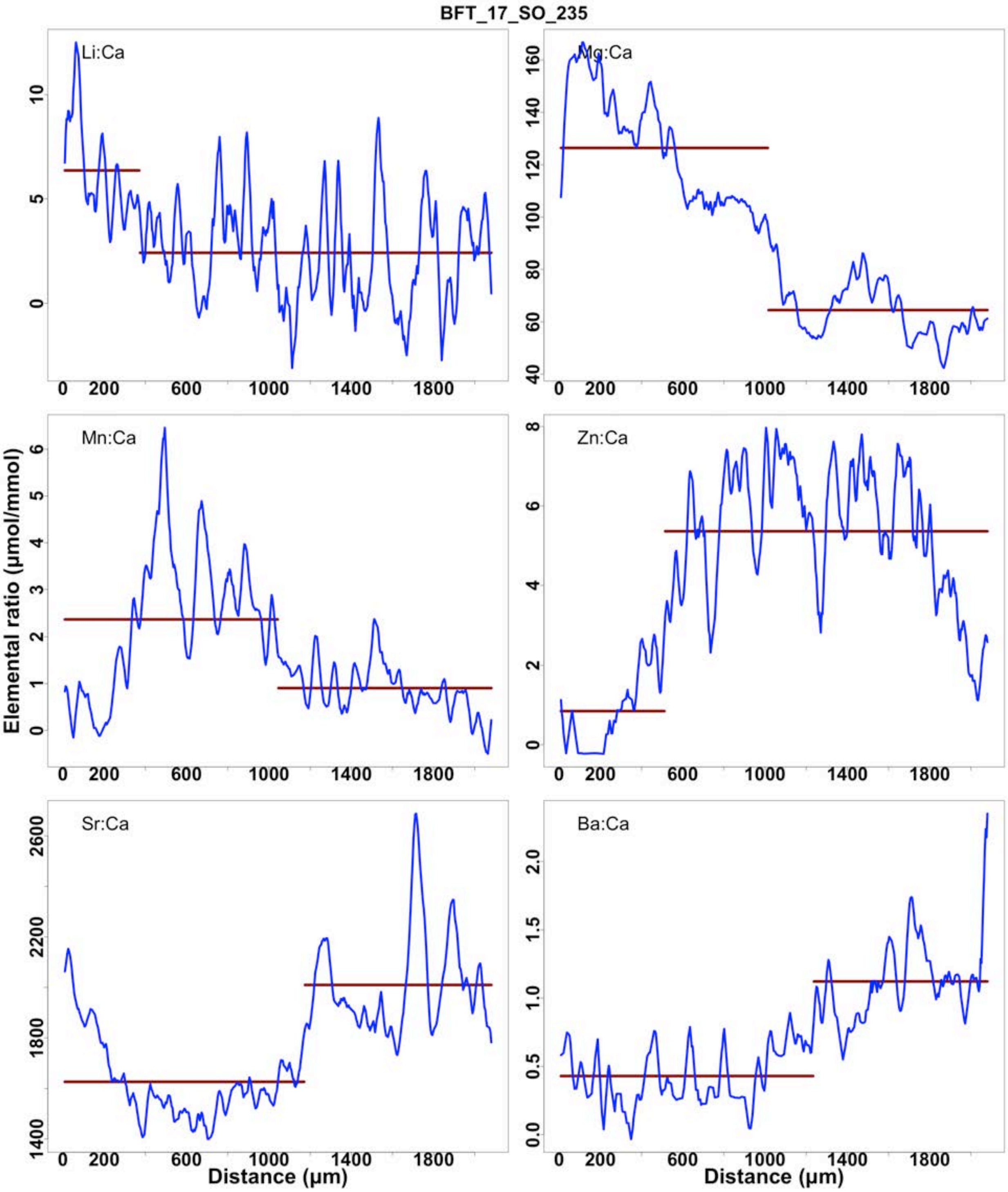

BFT_17_SO_235
Li:Ca
Mg:Ca
Mn:Ca
Zn:Ca
Sr:Ca
Ba:Ca
Elemental ratio (µmol/mmol)
Distance (µm)
Distance (µm)

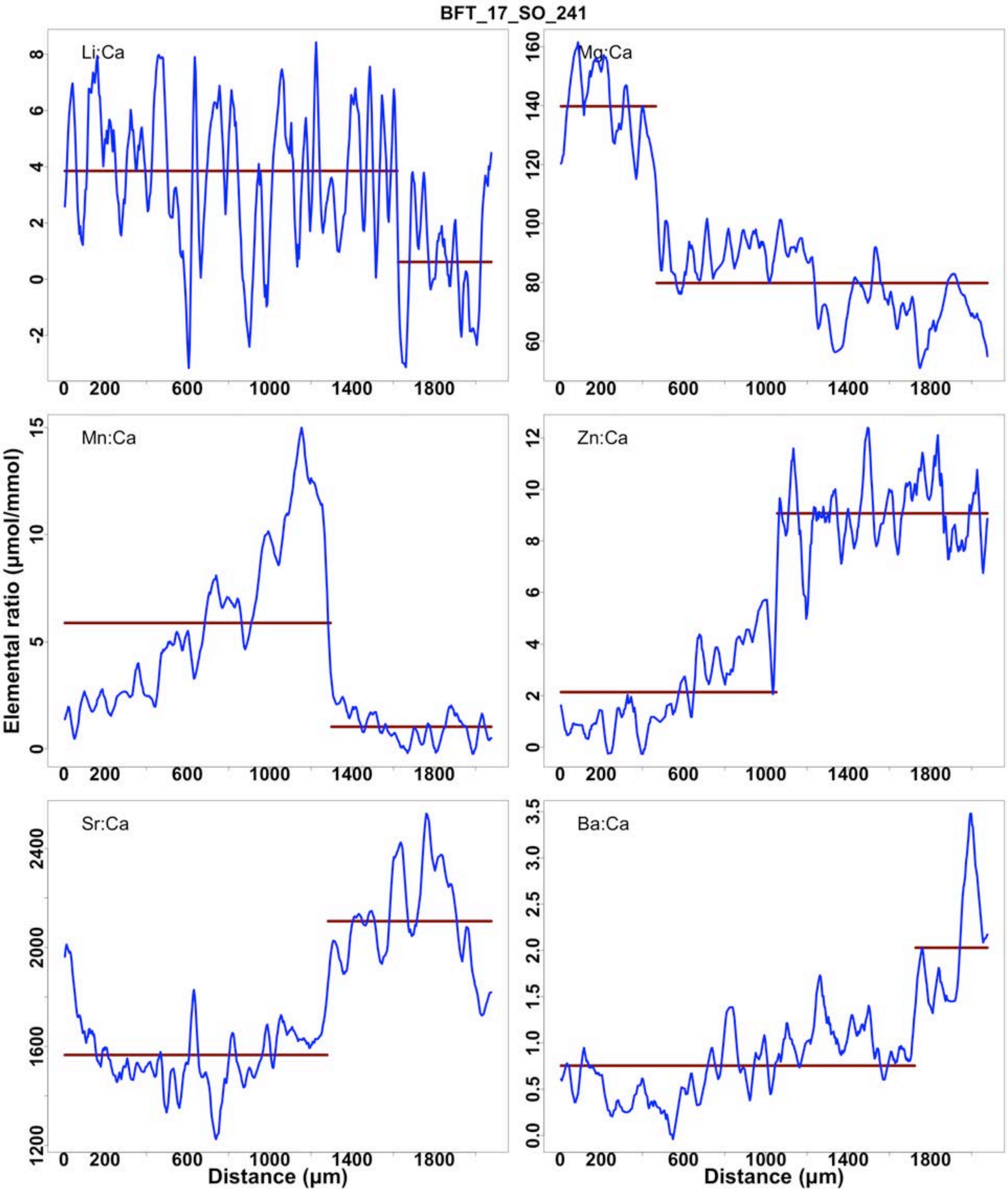
BFT_17_SO_241
Li:Ca
Mg:Ca
Mn:Ca
Zn:Ca
Sr:Ca
Ba:Ca
Elemental ratio (µmol/mmol)
Distance (µm)
Distance (µm)

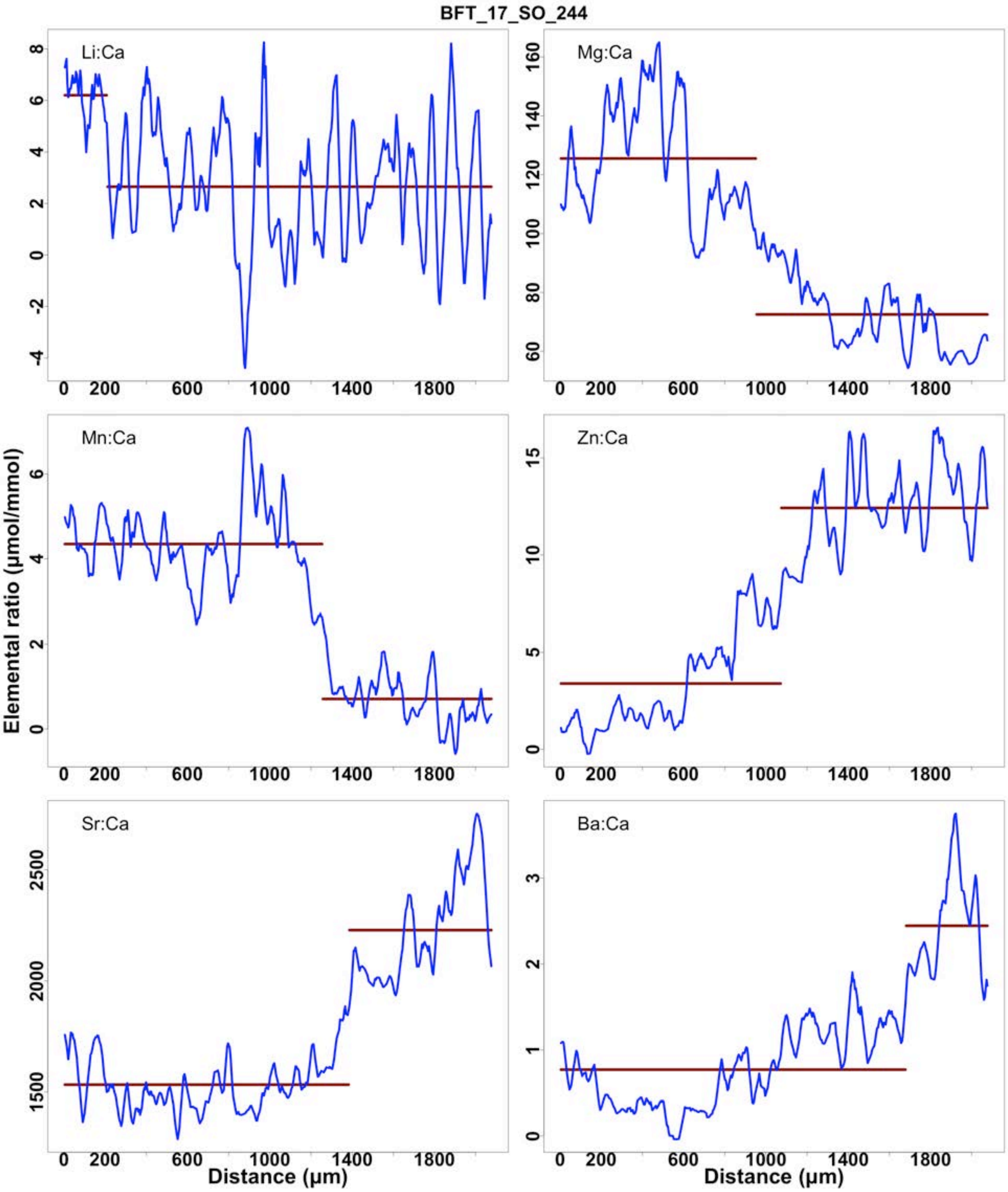

BFT_17_SO_244
Li:Ca
Mg:Ca
Mn:Ca
Zn:Ca
Sr:Ca
Ba:Ca
Elemental ratio (μmol/mmol)
Distance (μm)
Distance (μm)

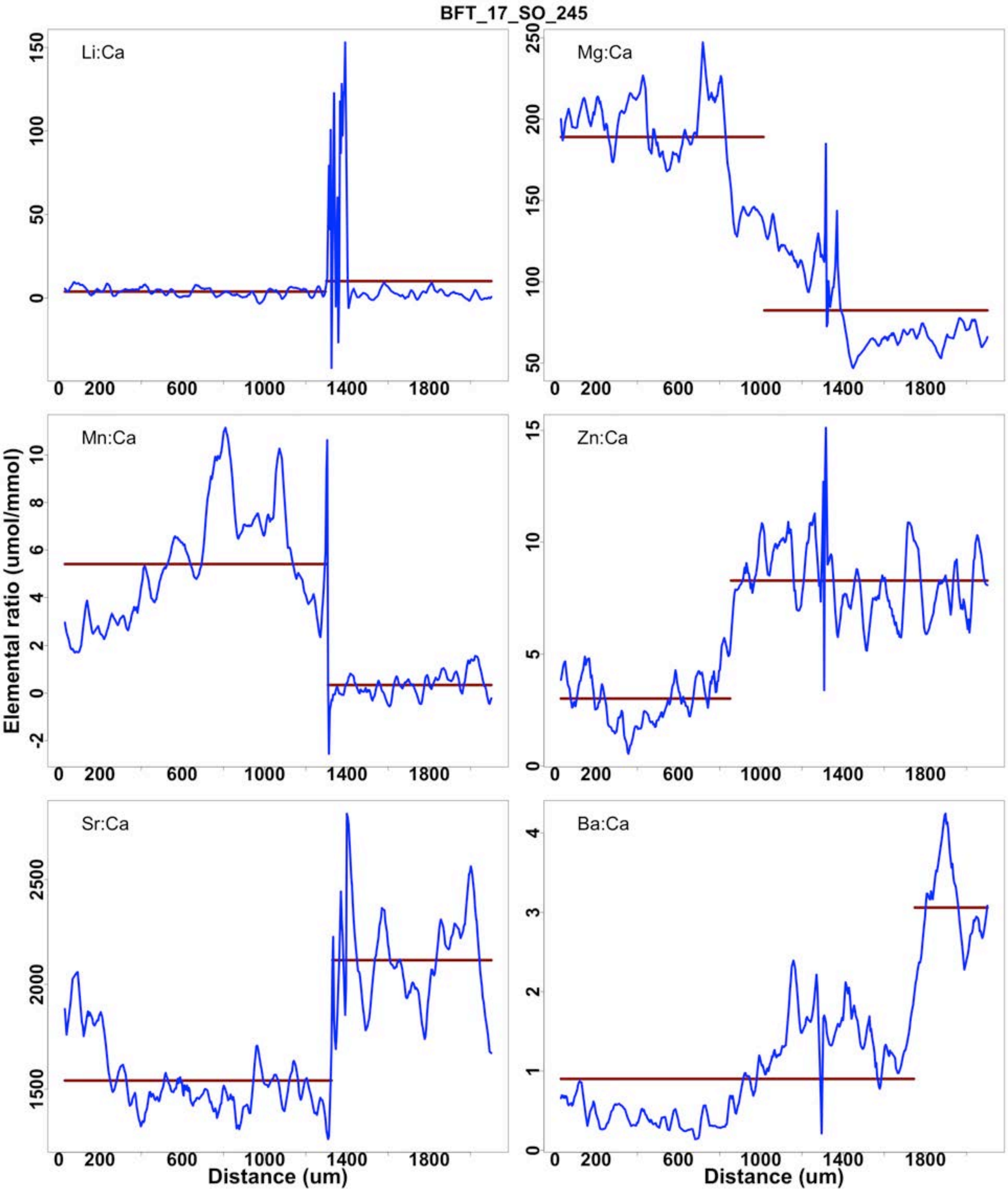
BFT_17_SO_245
Li:Ca
Mg:Ca
Mn:Ca
Zn:Ca
Sr:Ca
Ba:Ca
Elemental ratio (umol/mmol)
Distance (um)
Distance (um)

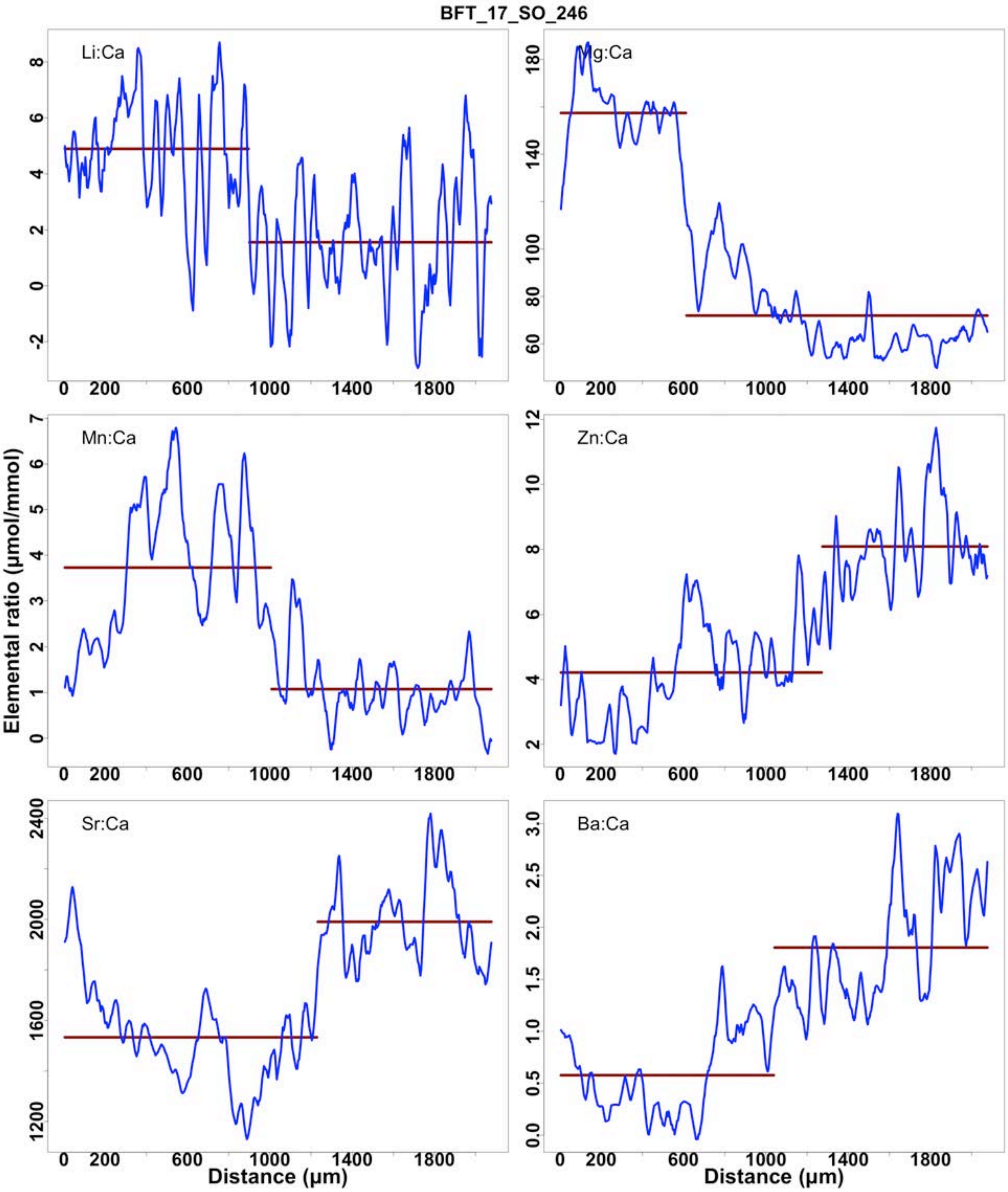

BFT_17_SO_246
Li:Ca
Mg:Ca
Mn:Ca
Zn:Ca
Sr:Ca
Ba:Ca
Elemental ratio (µmol/mmol)
Distance (µm)
Distance (µm)

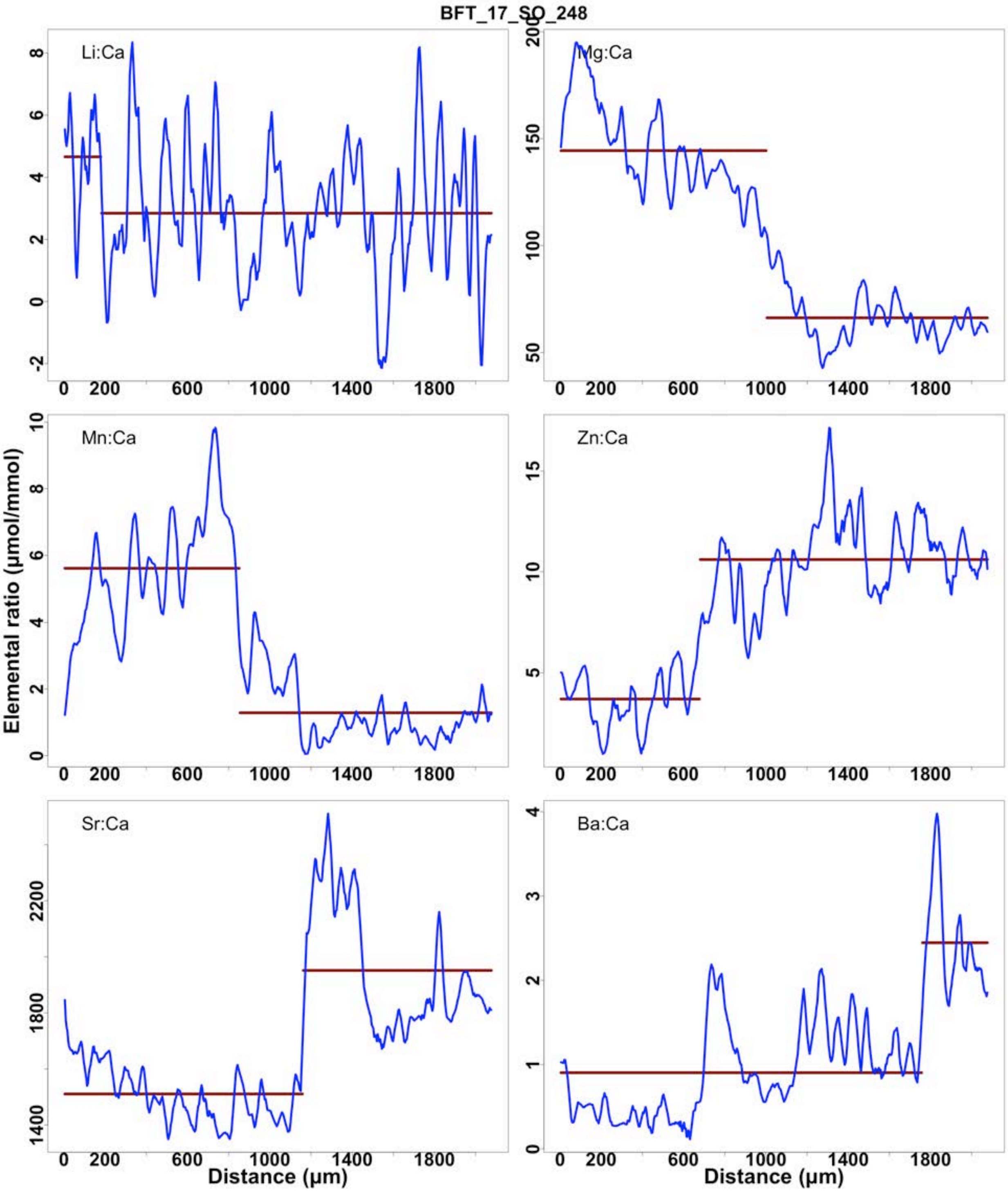
BFT_17_SO_248
Li:Ca
Mg:Ca
Mn:Ca
Zn:Ca
Sr:Ca
Ba:Ca
Elemental ratio (µmol/mmol)
Distance (µm)
Distance (µm)

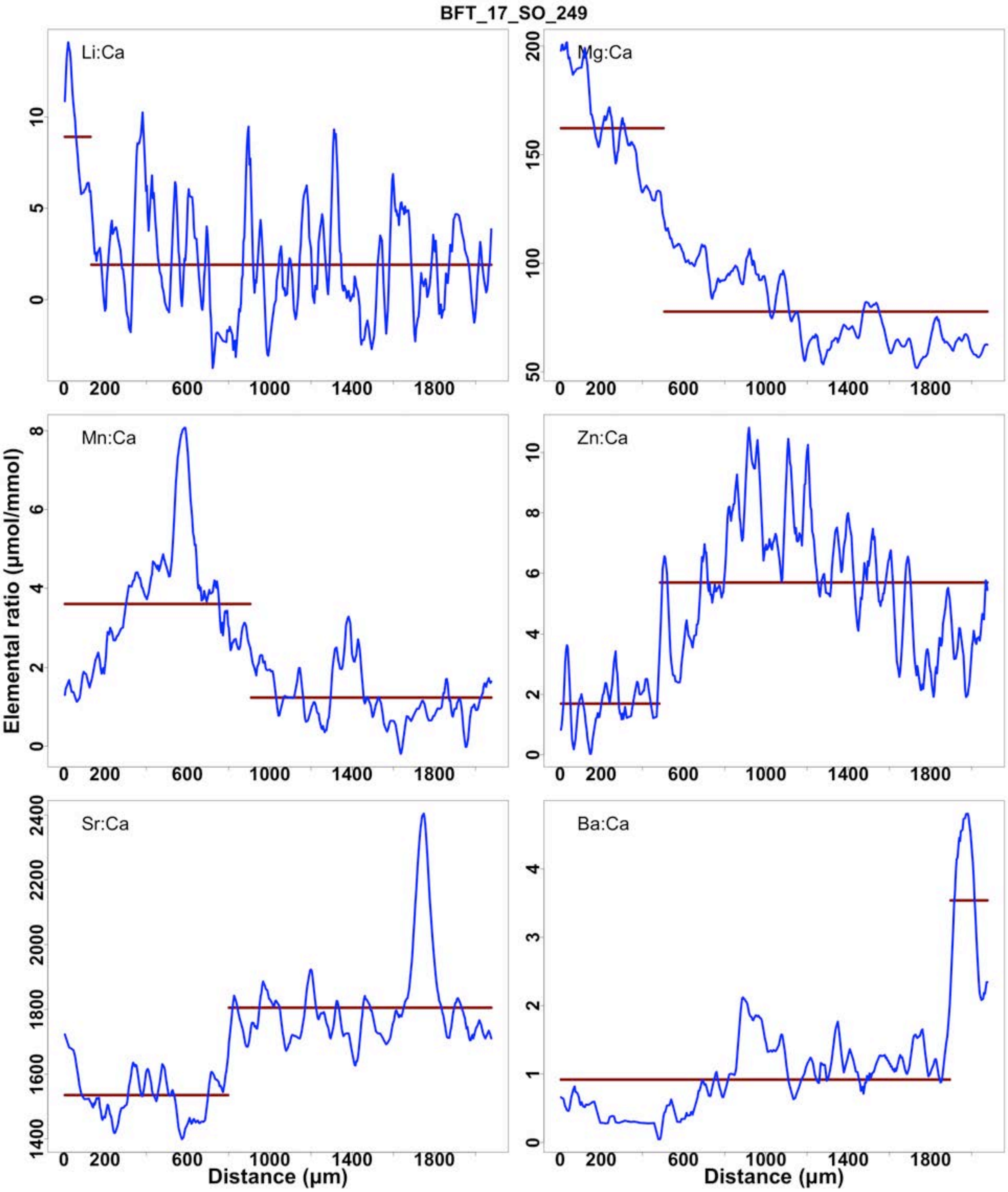
BFT_17_SO_249
Li:Ca
Mg:Ca
Mn:Ca
Zn:Ca
Sr:Ca
Ba:Ca
Elemental ratio (µmol/mmol)
Distance (µm)
Distance (µm)

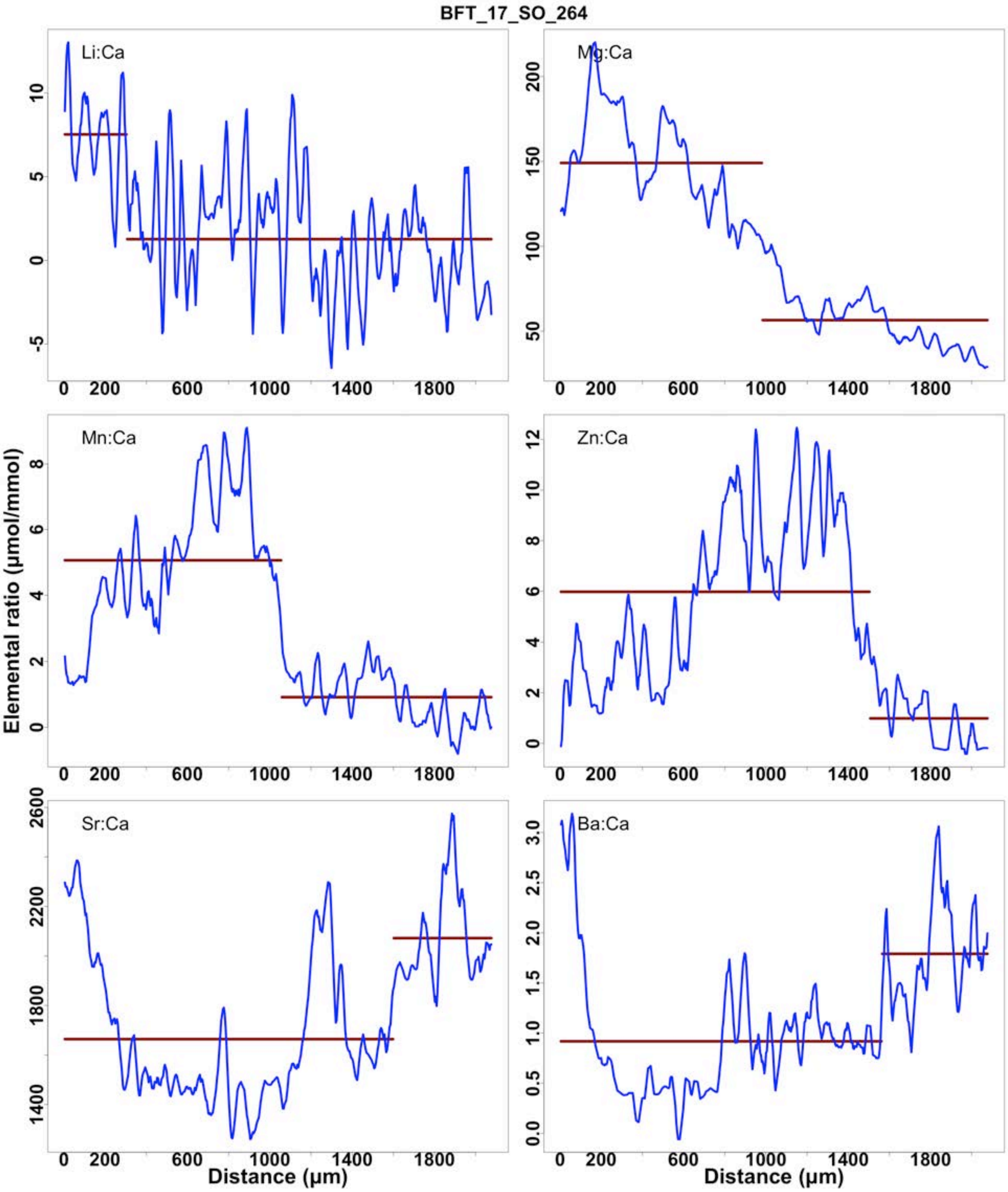
BFT_17_SO_264
Li:Ca
Mg:Ca
Mn:Ca
Zn:Ca
Sr:Ca
Ba:Ca
Elemental ratio (µmol/mmol)
Distance (µm)
Distance (µm)

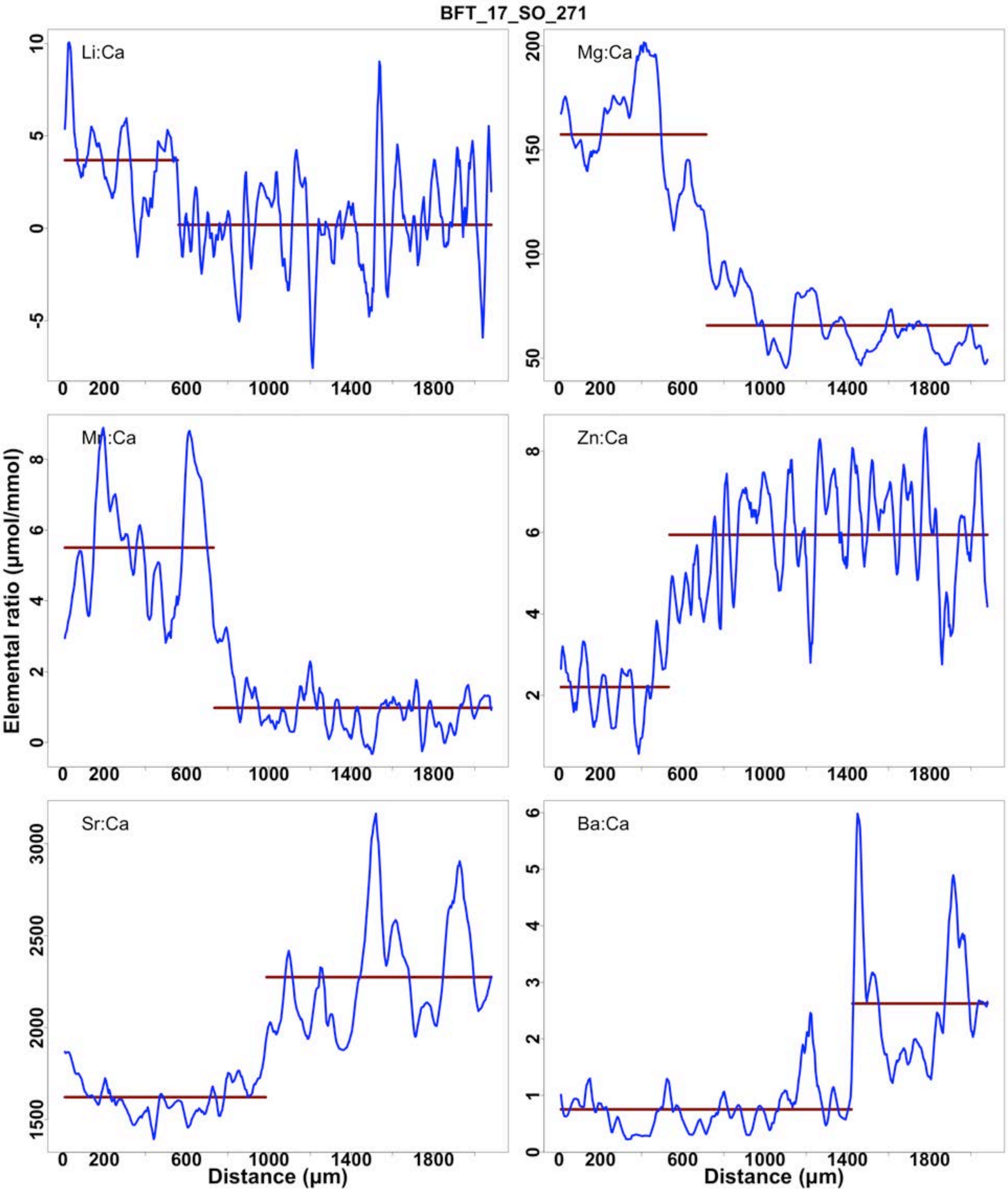
BFT_17_SO_271
Li:Ca
Mg:Ca
Mn:Ca
Zn:Ca
Sr:Ca
Ba:Ca
Elemental ratio (µmol/mmol)
Distance (µm)
Distance (µm)

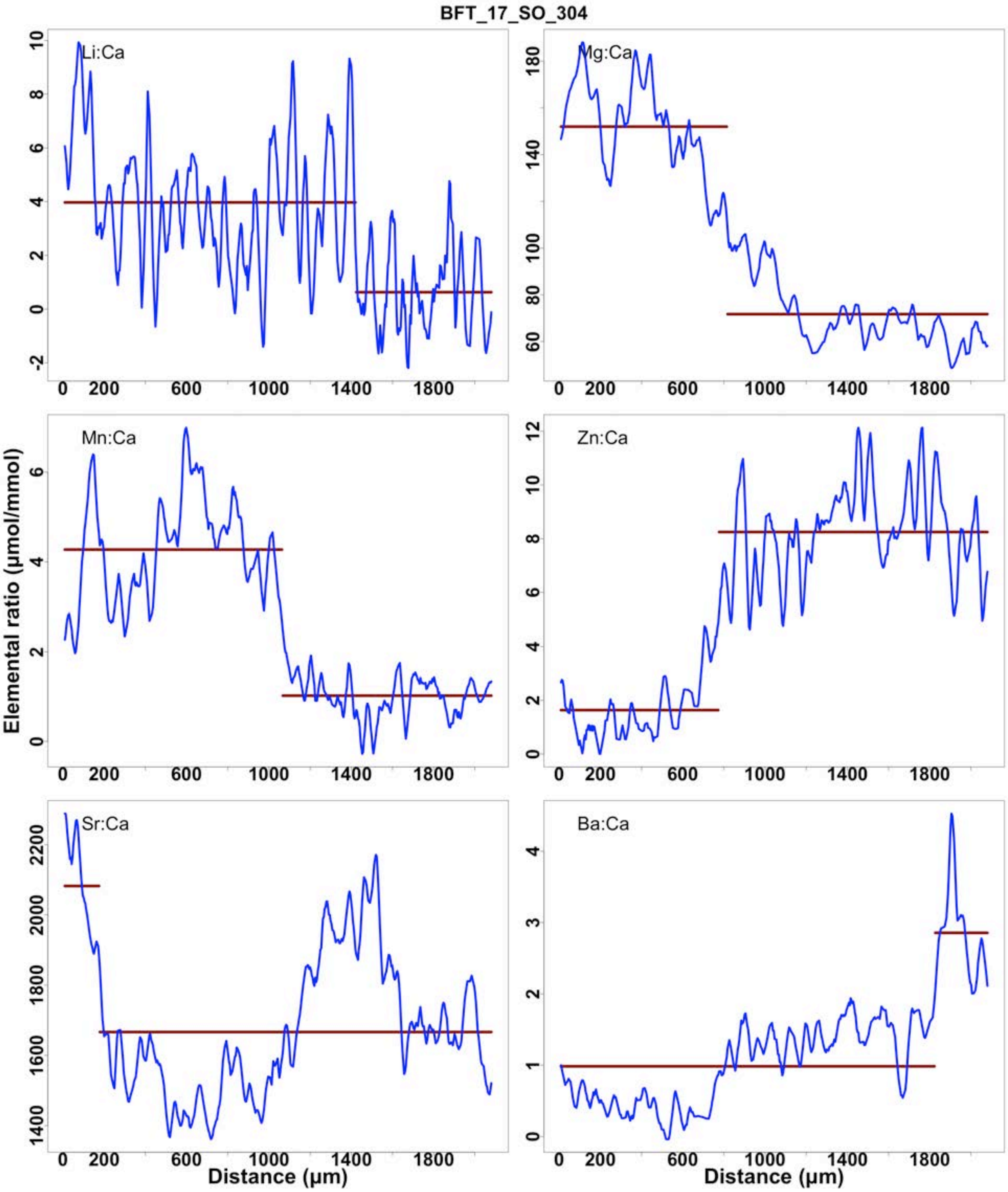

BFT_17_SO_304
Li:Ca
Mg:Ca
Mn:Ca
Zn:Ca
Sr:Ca
Ba:Ca
Elemental ratio (µmol/mmol)
Distance (µm)
Distance (µm)

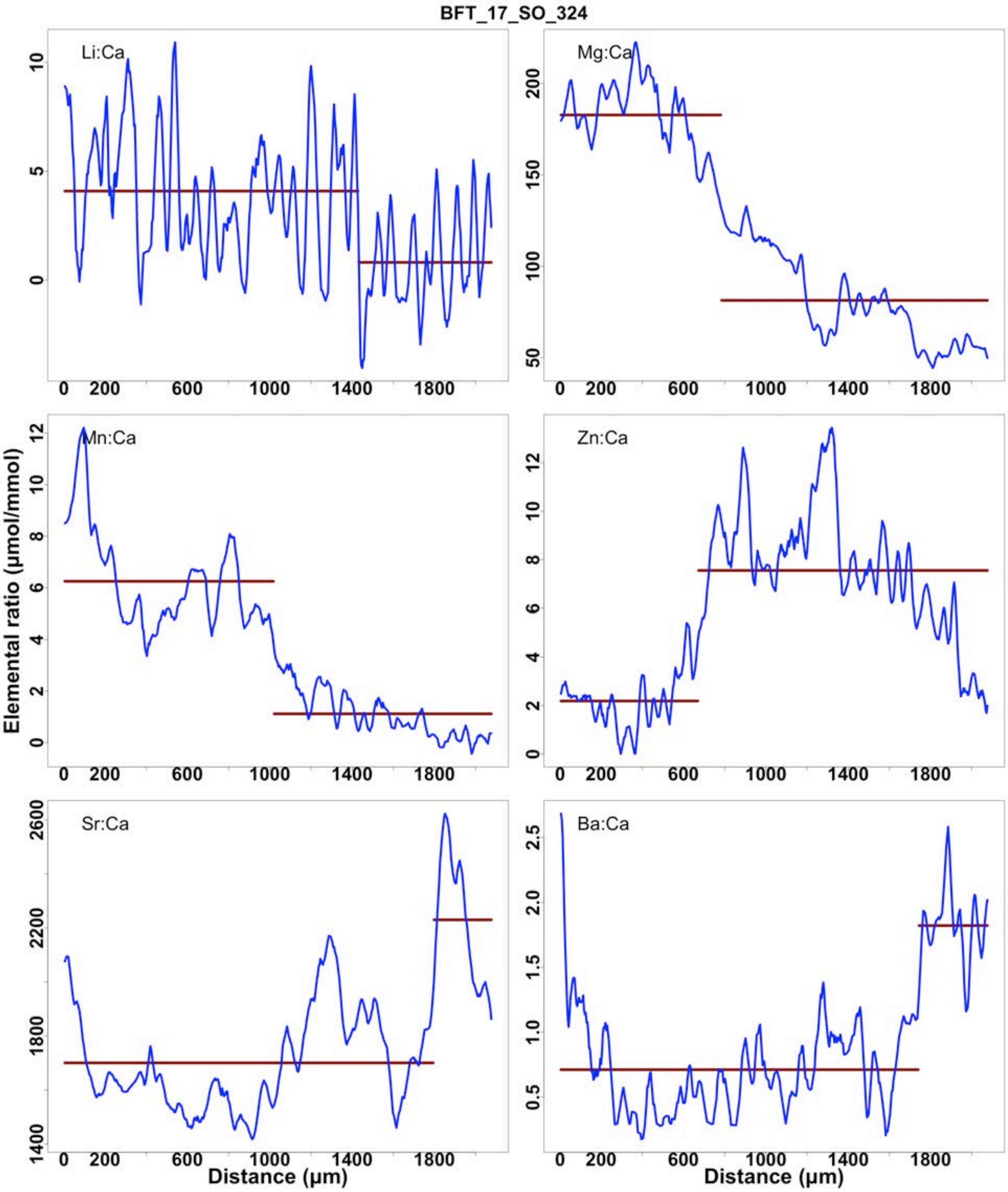

BFT_17_SO_324
Li:Ca
Mg:Ca
Mn:Ca
Zn:Ca
Sr:Ca
Ba:Ca
Elemental ratio (µmol/mmol)
Distance (µm)
Distance (µm)

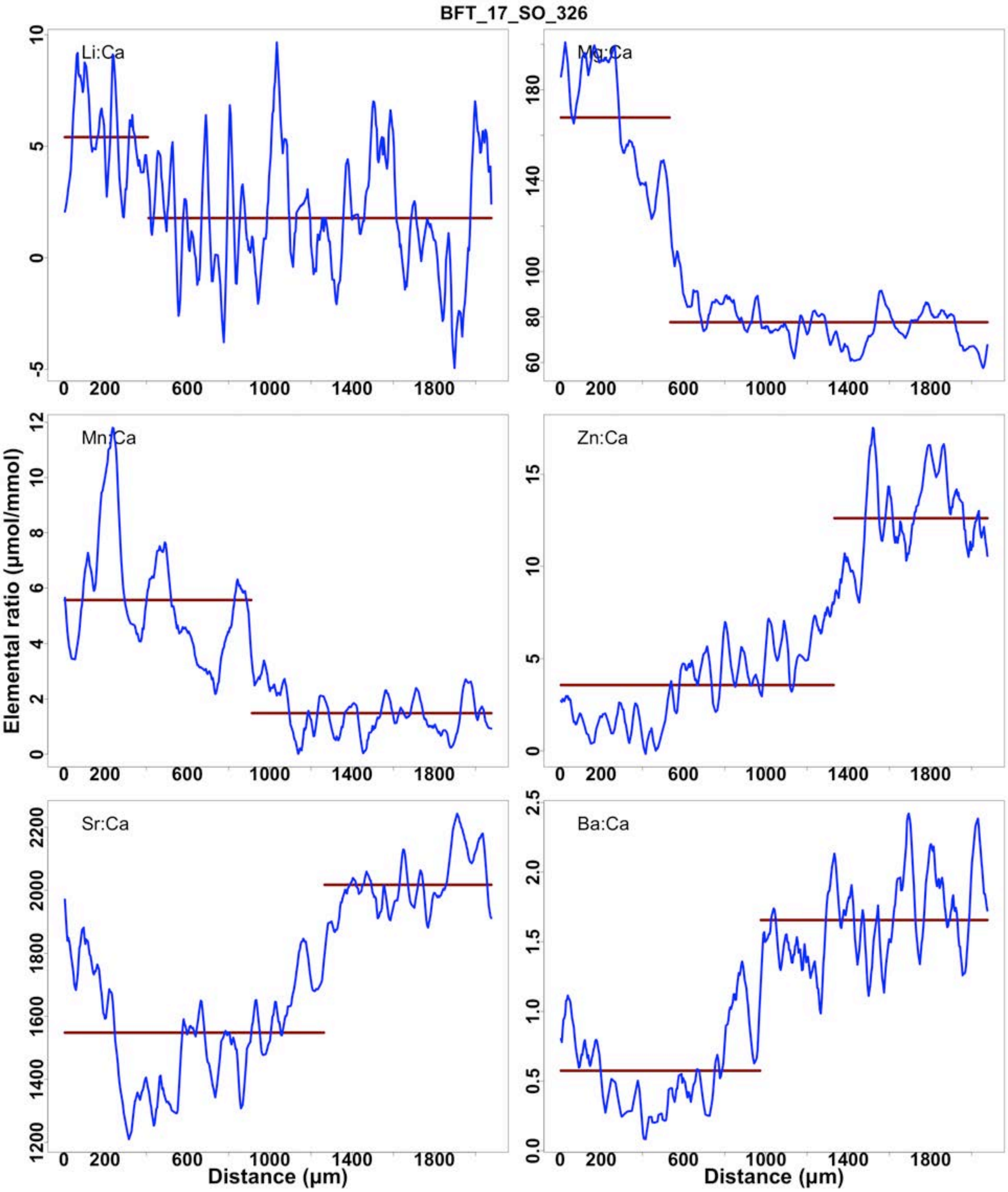

BFT_17_SO_326
Li:Ca
Mg:Ca
Mn:Ca
Zn:Ca
Sr:Ca
Ba:Ca
Elemental ratio (µmol/mmol)
Distance (µm)
Distance (µm)

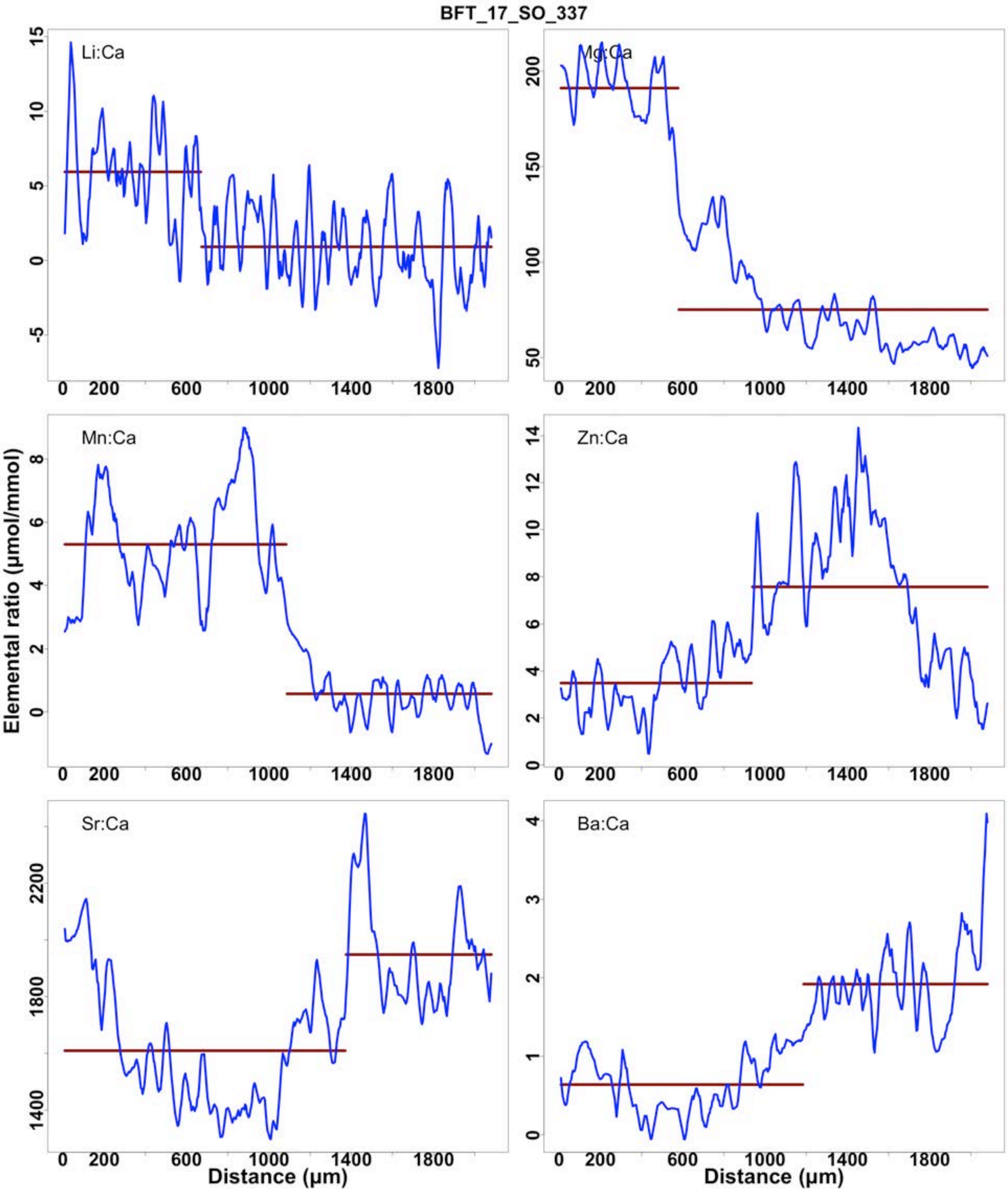
BFT_17_SO_337
Li:Ca
Mg:Ca
Mn:Ca
Zn:Ca
Sr:Ca
Ba:Ca
Elemental ratio (µmol/mmol)
Distance (µm)
Distance (µm)

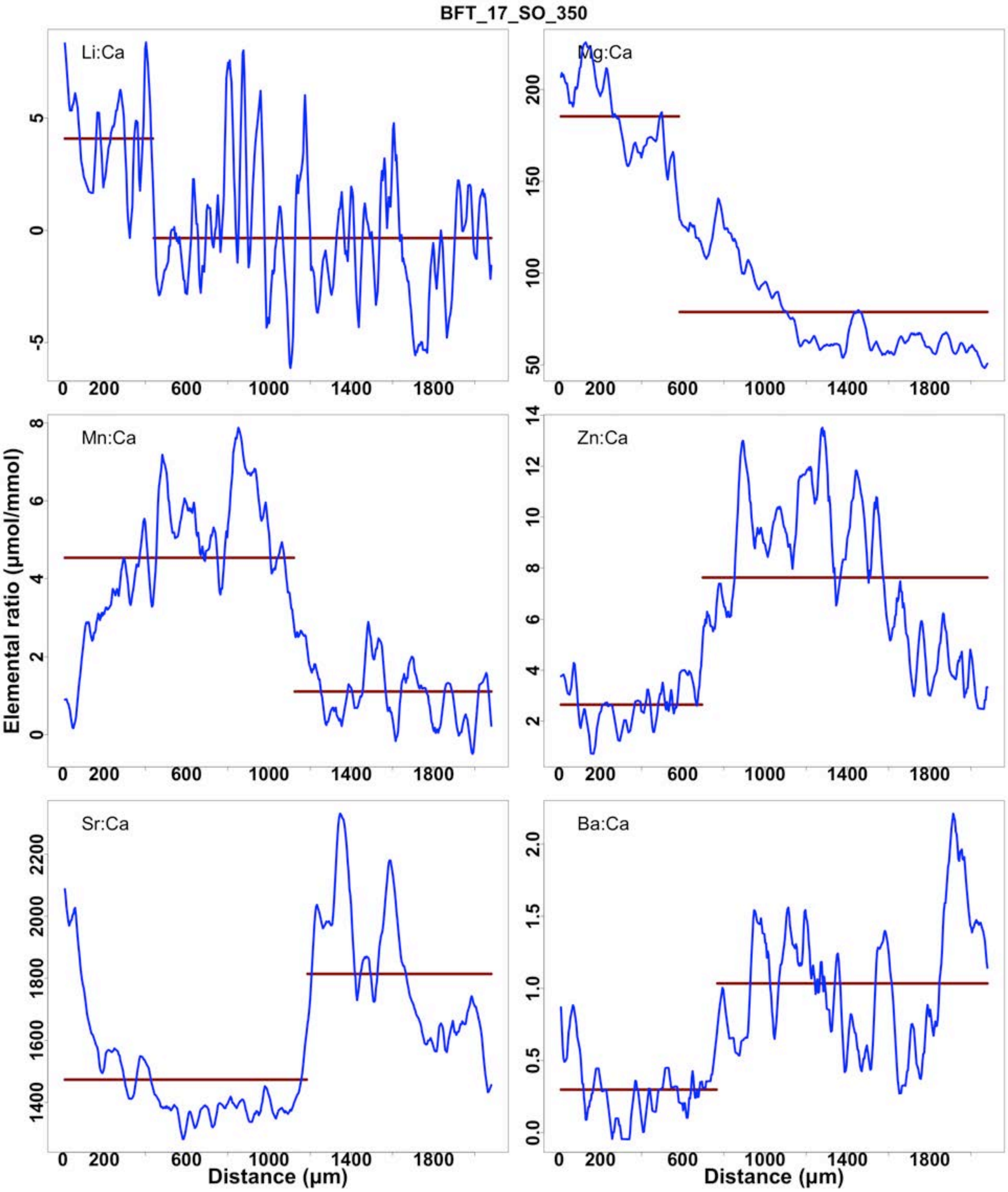
BFT_17_SO_350
Li:Ca
Mg:Ca
Mn:Ca
Zn:Ca
Sr:Ca
Ba:Ca
Elemental ratio (µmol/mmol)
Distance (µm)
Distance (µm)

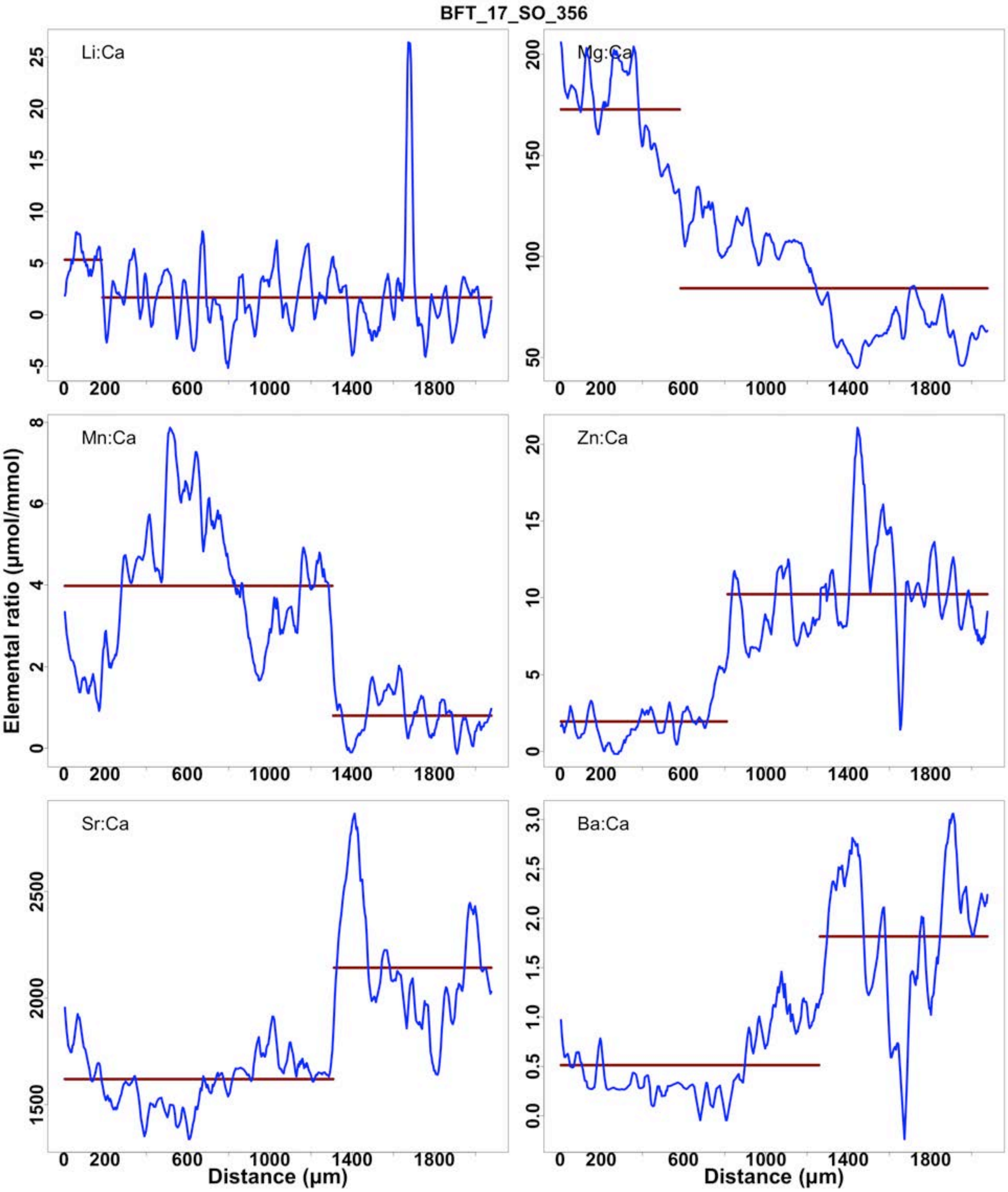
BFT_17_SO_356
Li:Ca
Mg:Ca
Mn:Ca
Zn:Ca
Sr:Ca
Ba:Ca
Elemental ratio (µmol/mmol)
Distance (µm)
Distance (µm)
0
200
600
1000
1400
1800

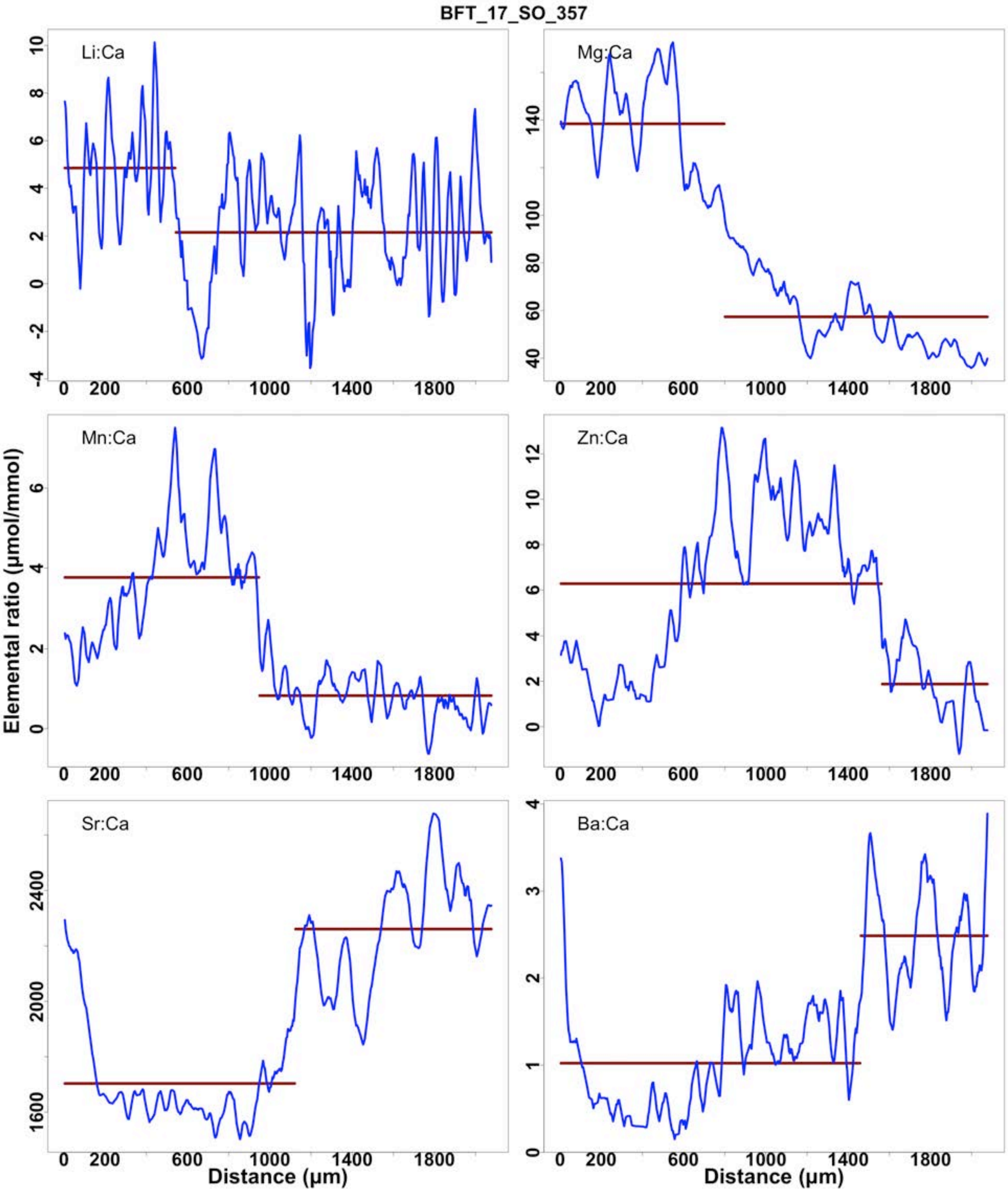
BFT_17_SO_357
Li:Ca
Mg:Ca
Mn:Ca
Zn:Ca
Sr:Ca
Ba:Ca
Elemental ratio (µmol/mmol)
Distance (µm)
Distance (µm)

BFT_17_SO_383

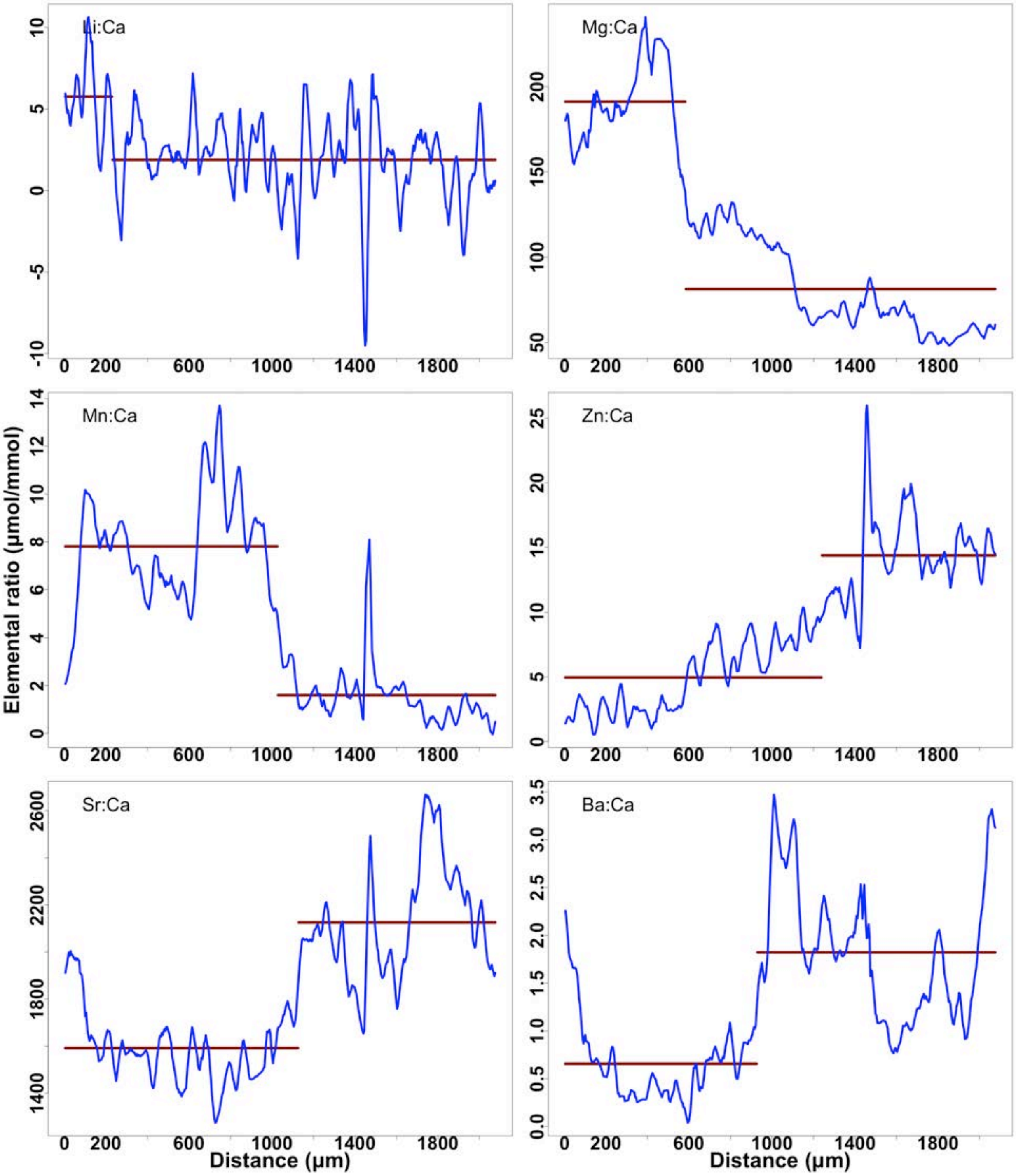

Li:Ca
Mg:Ca
Mn:Ca
Zn:Ca
Sr:Ca
Ba:Ca
Elemental ratio (µmol/mmol)
Distance (µm)
Distance (µm)

BFT_17_SO_386

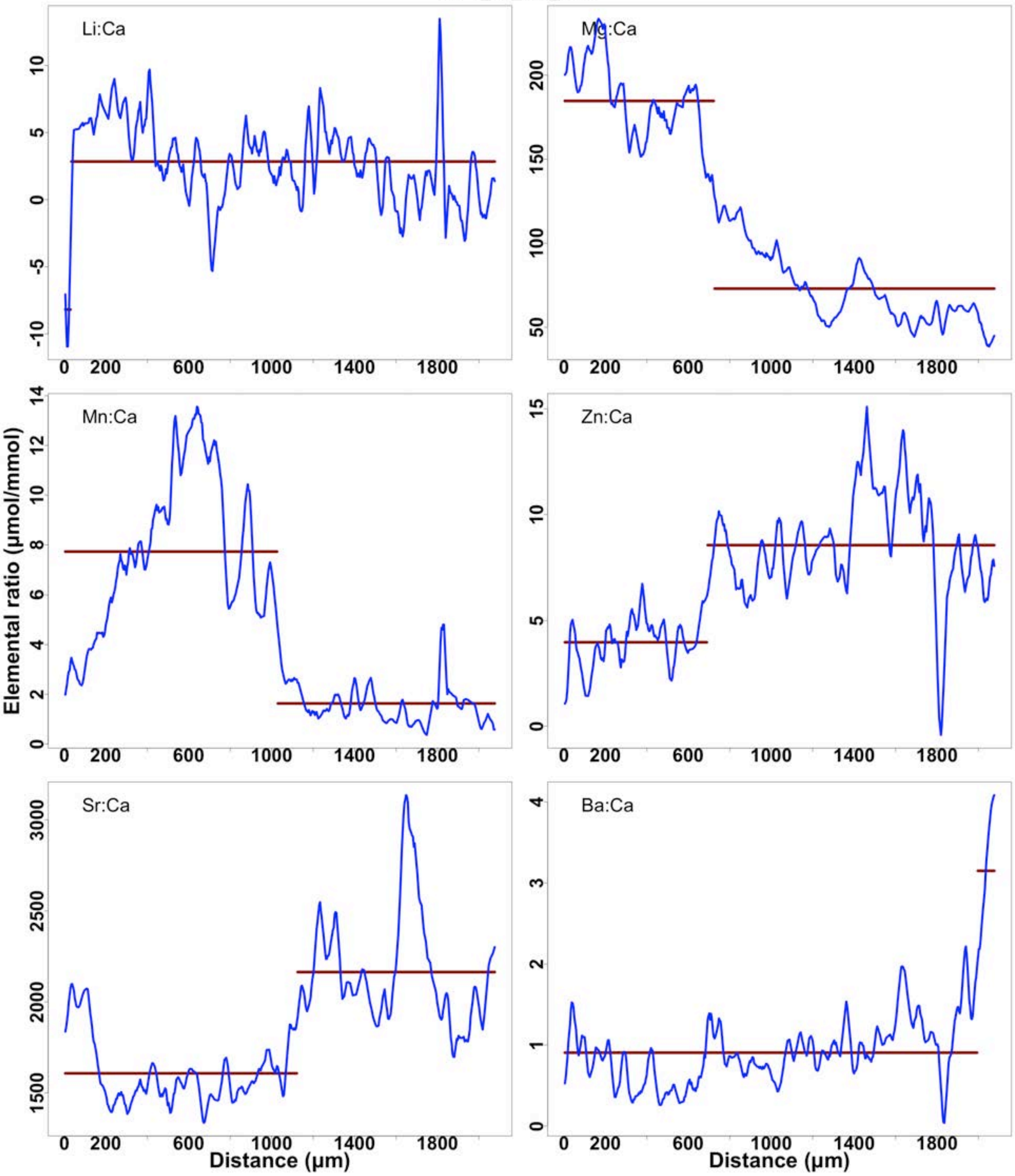

Li:Ca
Mg:Ca
Mn:Ca
Zn:Ca
Sr:Ca
Ba:Ca
Elemental ratio (µmol/mmol)
Distance (µm)
Distance (µm)

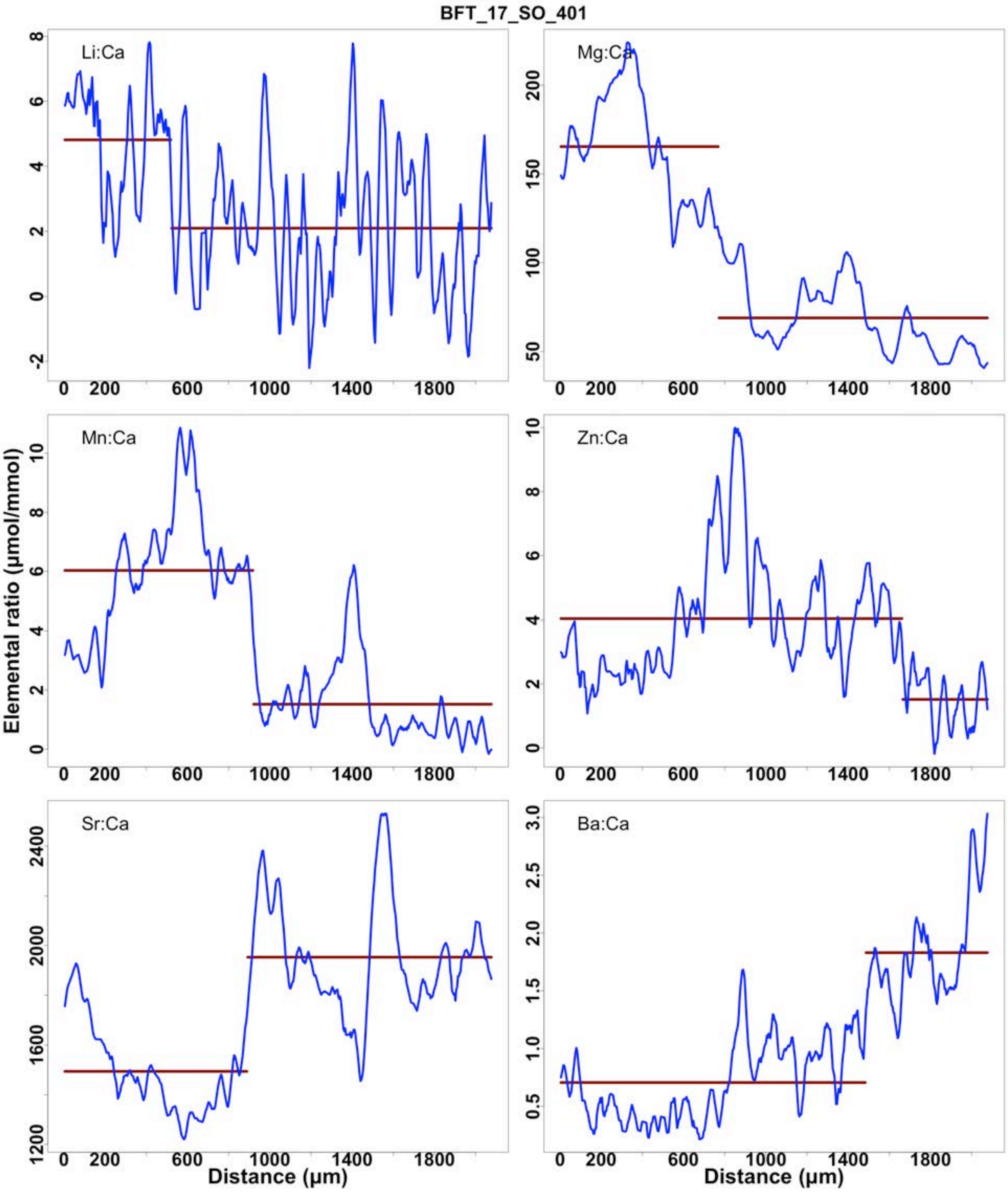
BFT_17_SO_401
Li:Ca
Mg:Ca
Mn:Ca
Zn:Ca
Sr:Ca
Ba:Ca
Elemental ratio (µmol/mmol)
Distance (µm)
Distance (µm)

BFT_17_SO_405

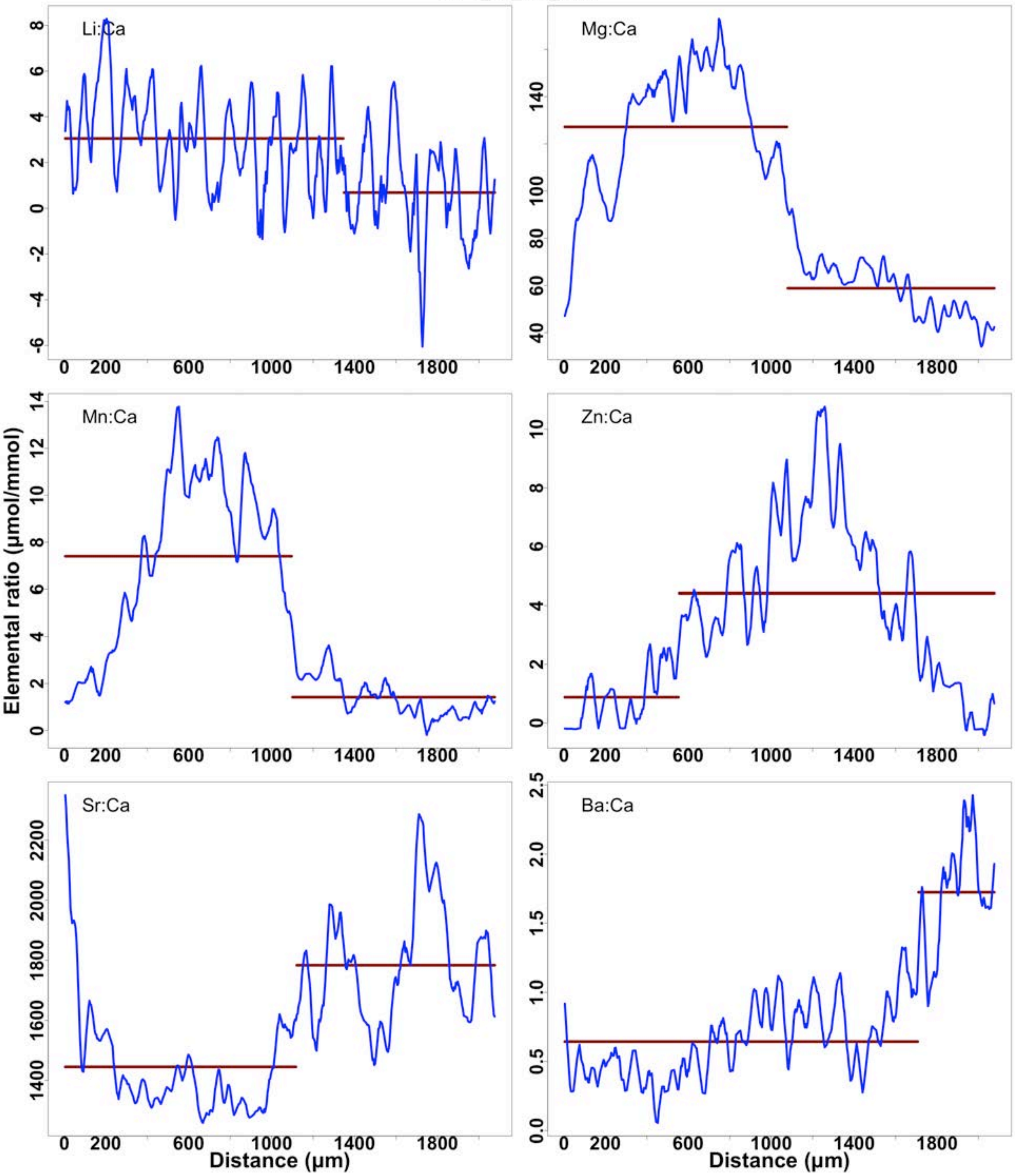

Li:Ca
Mg:Ca
Mn:Ca
Zn:Ca
Sr:Ca
Ba:Ca
Elemental ratio (µmol/mmol)
Distance (µm)
Distance (µm)

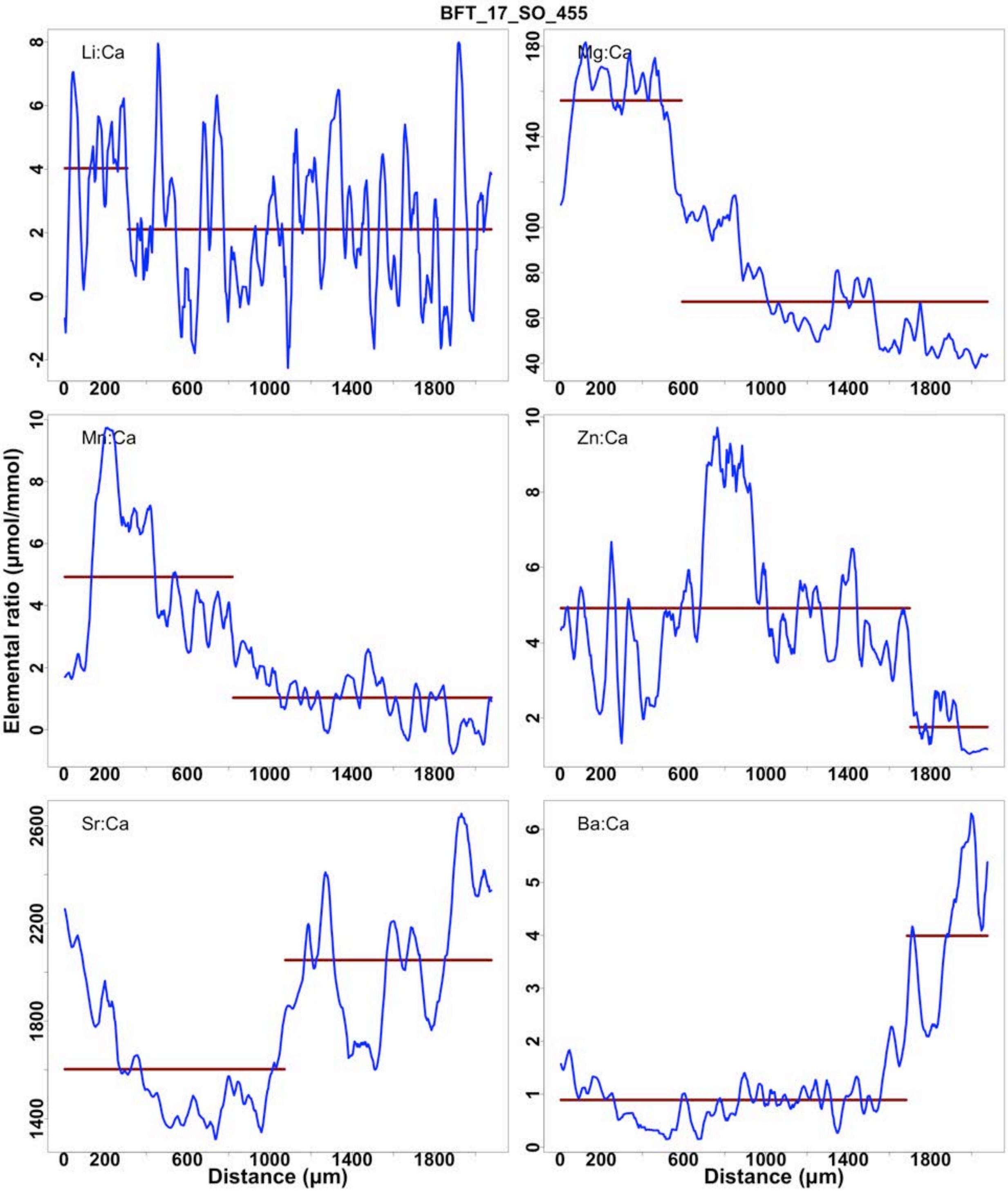

BFT_17_SO_455
Li:Ca
Mg:Ca
Mn:Ca
Zn:Ca
Sr:Ca
Ba:Ca
Elemental ratio (µmol/mmol)
Distance (µm)
Distance (µm)

BFT_17_SO_463

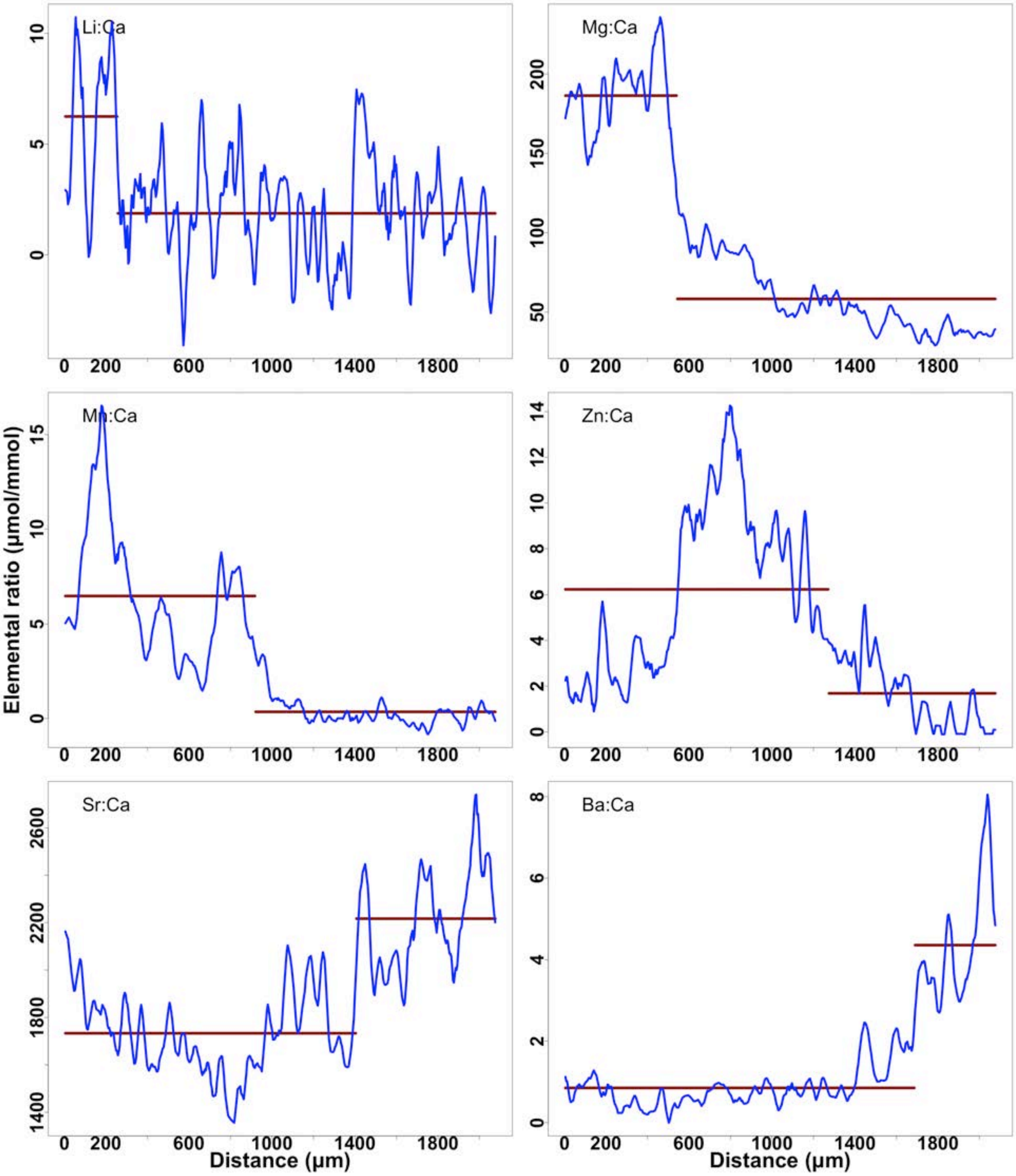
Li:Ca
Mg:Ca
Mn:Ca
Zn:Ca
Sr:Ca
Ba:Ca
Elemental ratio (µmol/mmol)
Distance (µm)
Distance (µm)

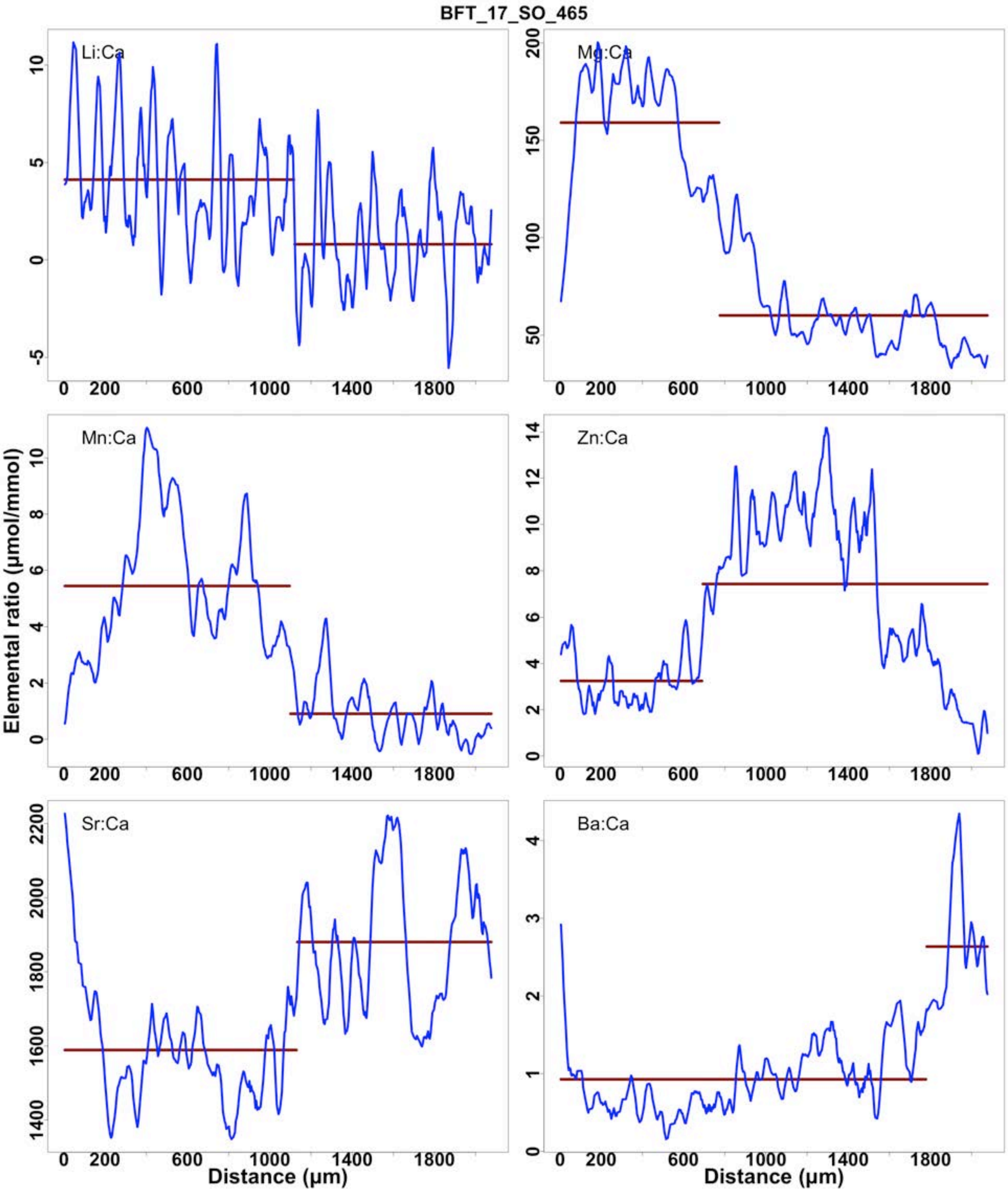
BFT_17_SO_465
Li:Ca
Mg:Ca
Mn:Ca
Zn:Ca
Sr:Ca
Ba:Ca
Elemental ratio (µmol/mmol)
Distance (µm)
Distance (µm)

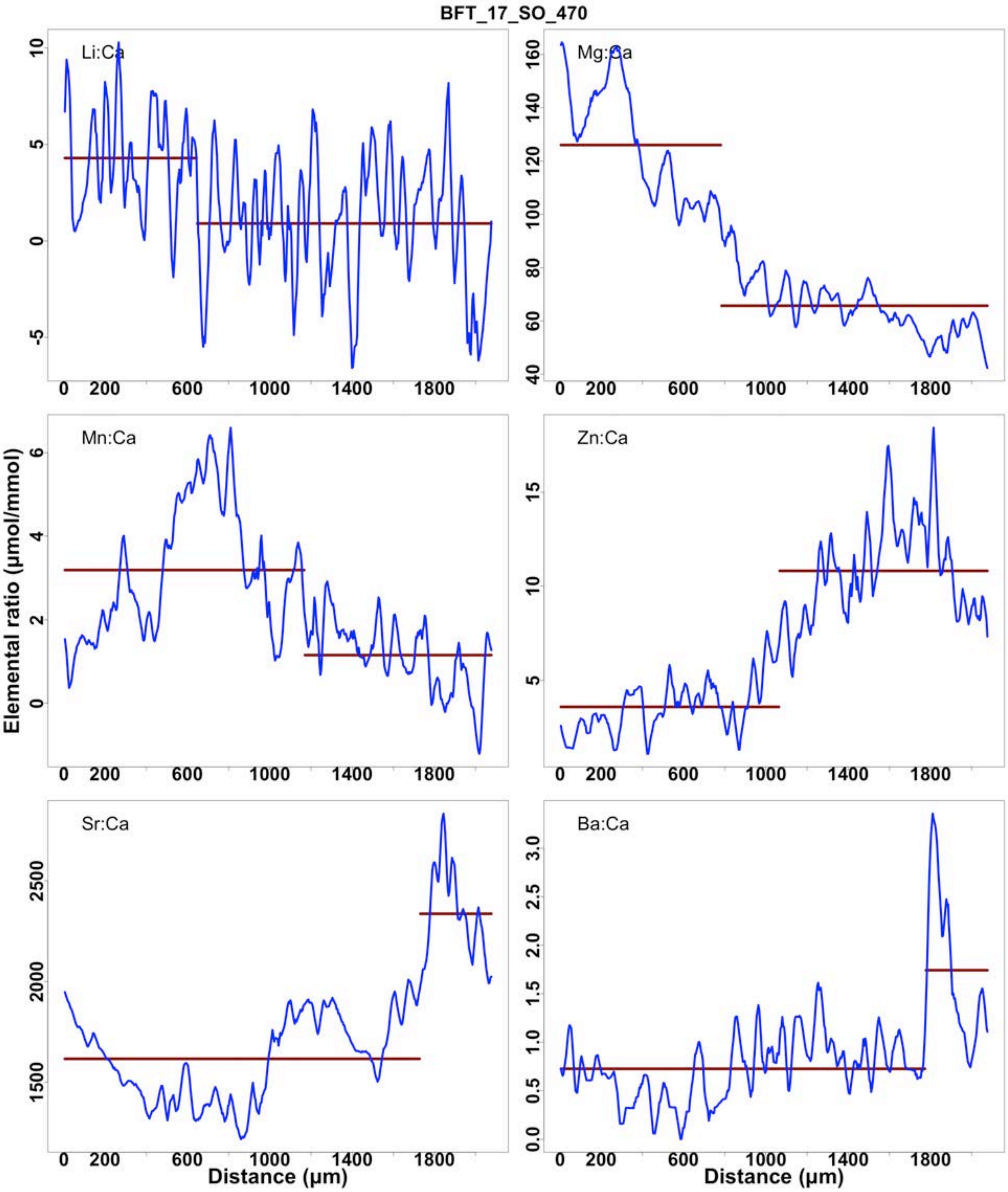
BFT_17_SO_470
Li:Ca
Mg:Ca
Mn:Ca
Zn:Ca
Sr:Ca
Ba:Ca
Elemental ratio (µmol/mmol)
Distance (µm)
Distance (µm)
