# Supplementary material for: Natal origin and age-specific egress of Pacific bluefin tuna from coastal nurseries revealed with geochemical markers
Source: Sci Rep. 2021 Jul 9;11:14216. doi: 10.1038/s41598-021-93298-2 (PMC8270904; doi:10.1038/s41598-021-93298-2)
Supplement: Supplementary file 2 — Supplementary Information 2. [file 41598_2021_93298_MOESM2_ESM.docx]

**Natal origin and age-specific egress of Pacific bluefin tuna from coastal nurseries revealed with geochemical markers**

Jay R. Rooker^1,2*^, R. J. David Wells^1,2^, Barbara A. Block^3^, Hui Liu^1^, Hannes Baumann^4^, Wei-Chuan Chiang^5^, Michelle Sluis Zapp^1^, Nathaniel R. Miller^6^, John A. Mohan^1^, Seiji Ohshimo^7^, Yosuke Tanaka^8^, Michael A. Dance^9^, Heidi Dewar^10^, Owyn E. Snodgrass^10^, Jen-Chieh Shiao^11^

^1^Department of Marine Biology, Texas A&M University at Galveston, 200 Seawolf Parkway, Galveston, TX 77553, USA

^2^Department of Ecology and Conservation Biology, Texas A&M University, College Station, TX 77843-2258

^3^Hopkins Marine Station, Stanford University, 120 Oceanview Boulevard, Pacific Grove, CA 93950 USA

^4^Department of Marine Sciences, University of Connecticut, 1080 Shennecossett Road, Groton, CT 06340-6048, USA

^5^Council of Agriculture, Fisheries Research Institute TW, 199 Hou-Ih Road, Keelung, 20246, Taiwan

^6^Jackson School of Geosciences, The University of Texas at Austin, 2275 Speedway Stop C9000, Austin, TX 78712, USA

^7^Pelagic Fish Resources Division, Fisheries Stock Assessment Center,  Fisheries Resources Institute, Japan Fisheries Research and Education Agency 1551-8, Taira-machi, Nagasaki, 851-2213 Japan

^8^Highly Migratory Resources Division, Fisheries Stock Assessment Center, Fisheries Resources Institute, Japan Fisheries Research and Education Agency 5-7-1, Orido, Shimizu, Shizuoka, 424-8633, Japan

^9^Department of Oceanography and Coastal Sciences, Louisiana State University, 2255 Energy, Coast and Environment Building Baton Rouge, Louisiana 70803, USA

^10^Southwest Fisheries Science Center, National Marine Fisheries Service, 8901 La Jolla Shores Drive, La Jolla, CA 92037, USA

^11^Institute of Oceanography, National Taiwan University, Taipei, No. 1, Sec. 4, Roosevelt Rd, Taipei 10617, Taiwan

**Table S1**. Summary statistics of age-0 and adult Pacific bluefin tuna (PBT) analyzed from the East China Sea and Sea of Japan. Sample size (N), collection year and month(s), mean fork length (cm FL ± 1 SD) shown.

| **Age Class** | **Location** | **N** | **Year** | **Month** | **Mean FL (cm)** | **1 SD** |
| --- | --- | --- | --- | --- | --- | --- |
| Age-0 | East China Sea | 10 | 2011 | Nov | 42.5 | 6.3 |
| Age-0 | East China Sea | 10 | 2012 | Nov-Dec | 45.5 | 1.9 |
| Age-0 | Sea of Japan | 10 | 2011 | Sep-Oct | 27.8 | 1.5 |
| Age-0 | Sea of Japan | 10 | 2012 | Jun-Oct | 32.6 | 6.6 |
|  |  |  |  |  |  |  |
| Adult | East China Sea | 56 | 2017 | May-Jun | 227.9 | 21.2 |

**Table S2**. Summary statistics for the 56 adult Pacific bluefin tuna used to develop geochemical chronologies for changepoint analysis. Collection date, length (cm FL), weight (kg), latitude and longitude of capture location, gender, and estimate age shown.

| **Otolith number** | **Date** | **Length(cm)** | **Weight(kg)** | **LAT** | **LON** | **Gender** | ***Age** |
| --- | --- | --- | --- | --- | --- | --- | --- |
| 2017-SO-008 | 5/1/17 | 244.0 | 287.0 | 25.10 | 123.29 | F | ≥15 |
| 2017-SO-019 | 5/5/17 | 229.0 | 224.0 | 25.12 | 123.57 | M | 14 |
| 2017-SO-027 | 5/6/17 | 202.0 | 149.0 | 24.56 | 123.27 | F | 8 |
| 2017-SO-036 | 5/8/17 | 226.0 | 246.0 | 25.18 | 124.24 | F | 12 |
| 2017-SO-078 | 5/13/17 | 273.0 | 388.0 | 25.40 | 125.39 | F | >15 |
| 2017-SO-093 | 5/15/17 | 201.0 | 157.0 | 25.42 | 125.20 | M | 8 |
| 2017-SO-103 | 5/16/17 | 244.0 | 265.0 | 25.44 | 125.28 | F | ≥15 |
| 2017-SO-140 | 5/17/17 | 208.0 | 168.0 | 25.40 | 125.22 | F | 10 |
| 2017-SO-182 | 5/22/17 | 179.0 | 112.0 | 23.52 | 122.48 | F | 7 |
| 2017-SO-193 | 5/24/17 | 220.0 | 182.0 | 25.70 | 123.49 | F | 12 |
| 2017-SO-196 | 5/25/17 | 197.0 | 141.0 | 25.21 | 124.70 | F | 8 |
| 2017-SO-197 | 5/25/17 | 217.0 | 190.0 | 25.50 | 125.90 | M | 11 |
| 2017-SO-199 | 5/25/17 | 203.0 | 192.0 | 25.24 | 124.33 | F | 9 |
| 2017-SO-200 | 5/25/17 | 212.0 | 172.0 | 25.30 | 123.41 | F | 10 |
| 2017-SO-205 | 5/26/17 | 230.0 | 195.0 | 25.19 | 124.10 | F | ≥15 |
| 2017-SO-206 | 5/26/17 | 223.0 | 175.0 | 25.37 | 124.48 | F | 12 |
| 2017-SO-209 | 5/26/17 | 243.0 | 338.0 | 25.34 | 124.53 | M | ≥15 |
| 2017-SO-210 | 5/26/17 | 209.0 | 194.0 | 25.35 | 124.53 | F | 10 |
| 2017-SO-211 | 5/26/17 | 202.0 | 178.0 | 25.30 | 124.56 | F | 8 |
| 2017-SO-213 | 5/27/17 | 214.0 | 181.0 | 24.59 | 123.51 | F | 10 |
| 2017-SO-214 | 5/27/17 | 241.0 | 240.0 | 24.59 | 123.51 | M | ≥15 |
| 2017-SO-215 | 5/27/17 | 213.0 | 166.0 | 25.40 | 123.60 | F | 10 |
| 2017-SO-216 | 5/27/17 | 233.0 | 241.0 | 23.23 | 122.13 | F | ≥15 |
| 2017-SO-218 | 5/29/17 | 226.0 | 185.0 | 25.30 | 124.48 | F | 12 |
| 2017-SO-220 | 5/29/17 | 254.0 | 301.0 | 25.30 | 123.44 | F | ≥15 |
| 2017-SO-221 | 5/29/17 | 214.0 | 182.0 | 25.29 | 125.70 | M | 10 |
| 2017-SO-226 | 5/29/17 | 221.0 | 184.0 | 25.27 | 124.36 | F | 12 |
| 2017-SO-227 | 5/29/17 | 一 | 231.0 | 25.00 | 123.40 | F | 一 |
| 2017-SO-228 | 5/29/17 | 213.0 | 206.0 | 24.57 | 123.40 | M | 10 |
| 2017-SO-229 | 5/29/17 | 213.0 | 191.0 | 25.32 | 125.80 | F | 10 |
| 2017-SO-230 | 5/29/17 | 236.0 | 268.0 | 23.00 | 122.00 | M | ≥15 |
| 2017-SO-233 | 6/1/17 | 205.0 | 162.0 | 25.45 | 125.47 | F | 9 |
| 2017-SO-235 | 6/1/17 | 214.0 | 200.0 | 25.13 | 123.30 | F | 10 |
| 2017-SO-241 | 6/1/17 | 210.0 | 172.0 | 24.58 | 123.40 | F | 9 |
| 2017-SO-244 | 6/1/17 | 201.0 | 149.0 | 25.80 | 123.41 | F | 8 |
| 2017-SO-245 | 6/1/17 | 215.0 | 167.0 | 24.54 | 123.15 | F | 11 |
| 2017-SO-246 | 6/1/17 | 220.0 | 170.0 | 25.50 | 123.42 | F | 12 |
| 2017-SO-248 | 6/1/17 | 216.0 | 226.0 | 25.11 | 123.50 | M | 12 |
| 2017-SO-249 | 6/1/17 | 212.0 | 172.0 | 24.58 | 123.80 | F | 10 |
| 2017-SO-264 | 6/2/17 | 263.0 | 348.0 | 25.28 | 124.43 | M | ≥15 |
| 2017-SO-271 | 6/2/17 | 251.0 | 325.0 | 25.27 | 124.42 | M | ≥15 |
| 2017-SO-304 | 6/6/17 | 222.0 | 198.0 | 25.42 | 125.41 | M | 12 |
| 2017-SO-324 | 6/7/17 | 246.0 | 306.0 | 25.42 | 125.55 | F | ≥15 |
| 2017-SO-326 | 6/7/17 | 210.0 | 176.0 | 25.31 | 125.70 | M | 10 |
| 2017-SO-337 | 6/8/17 | 255.0 | 370.0 | 25.46 | 125.52 | M | ≥15 |
| 2017-SO-350 | 6/8/17 | 247.0 | 309.0 | 25.43 | 125.49 | M | ≥15 |
| 2017-SO-356 | 6/10/17 | 253.0 | 323.0 | 25.14 | 124.22 | M | ≥15 |
| 2017-SO-357 | 6/10/17 | 266.0 | 330.0 | 25.14 | 124.22 | M | ≥15 |
| 2017-SO-383 | 6/13/17 | 230.0 | 238.0 | 25.29 | 125.30 | F | 14 |
| 2017-SO-386 | 6/14/17 | 256.0 | 308.0 | 25.37 | 124.20 | F | ≥15 |
| 2017-SO-401 | 6/15/17 | 241.0 | 256.0 | 25.40 | 125.45 | F | ≥15 |
| 2017-SO-405 | 6/15/17 | 252.0 | 340.0 | 25.55 | 125.46 | M | ≥15 |
| 2017-SO-455 | 6/20/17 | 246.0 | 264.0 | 25.45 | 125.00 | F | ≥15 |
| 2017-SO-463 | 6/20/17 | 247.0 | 282.0 | 25.47 | 125.41 | F | ≥15 |
| 2017-SO-465 | 6/22/17 | 253.0 | 296.0 | 25.48 | 125.19 | M | ≥15 |
| 2017-SO-470 | 6/23/17 | 264.0 | 306.0 | 25.41 | 125.45 | M | ≥15 |
|  |  |  |  |  |  |  |  |

*Estimated from Shimose et al. 2009
